# A comparative study of microRNAs in different stages of *Eimeria tenella*

**DOI:** 10.3389/fvets.2022.954725

**Published:** 2022-07-22

**Authors:** Lei Zhang, Linlin Chen, Hongtao Zhang, Hongbin Si, Xianyong Liu, Xun Suo, Dandan Hu

**Affiliations:** ^1^College of Animal Science and Technology, Guangxi University, Nanning, China; ^2^Key Laboratory of Animal Epidemiology and Zoonosis of Ministry of Agriculture, National Animal Protozoa Laboratory, College of Veterinary Medicine, China Agricultural University, Beijing, China

**Keywords:** *Eimeria tenella*, miRNA, dicer, Argonaute, gene expression

## Abstract

Apicomplexan parasites have divergent biogenesis machinery for small RNA generation. Analysis has shown that parasites in *Plasmodium* and *Cryptosporidium* as well as many species in *Leishmania* or *Trypanosoma* do not have a complete machinery in small RNA biogenesis. Recently, the miRNA-generating system of *Toxoplasma* has been identified as plant/fungal-like and its miRNAome has been elucidated. However, the microRNA (miRNA) expression profiles and their potential regulatory functions in different stages of *Eimeria tenella* remain largely unknown. In this study, we characterized the RNA silencing machinery of *E. tenella* and investigated the miRNA population distribution at different life stages by high-throughput sequencing. We characterized the expression of miRNAs in the unsporulated oocyst, sporulated oocyst and schizogony stages, obtaining a total of 392 miRNAs. We identified 58 differentially expressed miRNAs between USO (unsporulated oocysts) and SO (sporulated oocysts) that were significantly enriched for their potential target genes in the regulation of gene expression and chromatin binding, suggesting an epigenetic modulation of sporulating by these miRNAs. In comparing miRNA expression at endogenous and exogenous developmental stages, twenty-four miRNAs were identified differently expressed. Those were mainly associated with the regulation of genes with protein kinase activity, suggesting control of protein phosphorylation. This is the first study about the evolution of miRNA biogenesis system and miRNA control of gene expression in *Eimeria* species. Our data may lead to functional insights into of the regulation of gene expression during parasite life cycle in apicomplexan parasites.

## Introduction

Coccidiosis in chickens caused by apicomplexan parasite *Eimeria* is a widespread intestinal disease (1). According to the latest estimates, the global cost of coccidiosis to the poultry industry is about £10 billion annually (2). *Eimeria* parasites have a complex developmental life cycle, with an exogenous phase in the environment, where oocysts excreted from chickens undergo differentiation (sporulation) and become infective, and an endogenous phase in the intestinal epithelial cells consisting of 3–5 rounds of schizogony, resulting in successive generations of schizonts (containing several merozoites), followed by sexual development and shedding of unsporulated oocysts (3).

MiRNAs are a class of non-coding RNA (ncRNA) that interact with messenger RNAs (mRNAs), leading to mRNA degradation or translation inhibition. It plays critical roles in a variety of biological processes, including development, metabolism, and apoptosis (4–6). In the biogenesis of miRNAs, primary miRNAs (pri-miRNAs) are transcribed by RNA polymerase II and undergo nuclear and cytoplasmic processing events, as carried out by the endoribonucleases Drosha and Dicer, respectively (7, 8). The mature miRNA, associated with the Argonaute (Ago) protein, is loaded onto the RNA-induced silencing complex (RISC) to interact with target mRNAs and regulate gene expression (9, 10). A single miRNA can silence multiple genes, while a single gene can be targeted by multiple miRNAs (11).

Apicomplexan parasites have divergent biogenesis machinery for small RNA generation. Argonaute and Dicer homologs were not found in *Cryptosporidium* and *Plasmodium* by analysis of their genome sequences, speculating that they do not have a mechanism for miRNA biosynthesis (12–14). This speculation is also supported by previous study (15). Examination of the available *Trypanosoma* genome sequences revealed the non-existence of identifiable remnants of DCL1, DCL2, or AGO1 homologs in *T. cruzi*. It was also found that there was no Dicer homolog in the *Leishmania* species (*L. major, L. donovani*), but a severely impaired pseudogene at the AGO1 locus was presented (16, 17). However, TbAGO1, a member of the Argonaute protein family, and two Dicer-like homologs, TbDCL1 and TbDCL2, were found in *T. brucei*. Meanwhile, endogenous small interfering RNAs or siRNAs were also identified (18). These findings also confirm the absence of a complete machinery underlying the generation of small RNA for many species of *Leishmania* or *Trypanosome*. In the study of *Toxoplasma*, the miRNA-generating system was identified as plant/fungal-like, and its miRNAome was elucidated (19). However, nothing is known about *Eimeria* miRNAs.

In this study, we characterized the RNA silencing machinery in *E. tenella* and investigated the distribution of miRNA population at different time periods by high-throughput sequencing. Different miRNAs and their potential target genes were analyzed between unsporulated oocysts, sporulated oocysts, and merozoites. A miRNA-mRNA interaction network was also constructed. This is the first study on *Eimeria* miRNAs, and our data will provide functional insights into parasite's lifecycle progression, and also basic knowledge for future studies on RNAi-dependent regulatory mechanisms in other apicomplexan parasites.

## Materials and Methods

### Ethical statement

The use of animals in this study was approved by the Administration Committee of Laboratory Animals in Guangxi University and was performed in accordance with the Institutional Animal Care and Use Committee guidelines (Approval Number: Gxu-2021-013).

### Parasites and animals

The *E. tenella* Houghton (ETH) strain was used throughout this work. Parasites were maintained and propagated by oral infection in 1-week-old broilers (Sanhuang chicken). Four-week-old AA broilers (Arbor Acres Poultry Breeding, Beijing, China) were used for the preparation of merozoites. Chickens were raised in a coccidia-free environment with *ad libitum* supply of filtered water and anticoccidial- and antibiotic-free feed. Procedures for parasite collection, purification and sporulation were carried out as described previously (20).

### Preparation of samples and extraction of total RNA

Four distinct developmental stages of the parasites were incorporated in this study: Unsporulated oocysts (USO), Sporulated oocysts (SO), merozoites at 108 h post-infection (Mer108) and merozoites at 120 h post-infection (Mer120). Purified unsporulated oocysts were collected from intestinal contents at 7 days post-infection (d.p.i.) from three groups of chicken (3 birds/group). Samples of sporulated oocysts were collected from the feces of three cages of chicken 9 d.p.i, and sporulated in 2.5% K_2_CrO_4_ (21). Caeca were collected from six groups of chicken at 108 and 120 h after infection, respectively. The merozoites for each sample were collected separately as reported by Schwarz et al. (22) with modifications. Briefly, sheared cecum was digested (0.50% sodium taurodeoxycholate hydrate and 0.25% trypsin in PBS) at 42°C for 30 min, filtered through gauze and centrifuged to obtain a precipitate containing dirty merozoites, which was further filtered to obtain clean merozoites. RNAs from all samples were extracted separately with Trizol regent, and genomic DNA was digested with DNase I (Qiagen, Hilden, Germany). Total RNAs were used for RNA-seq, and small RNA molecules were purified and used for miRNA-seq. RNA integrity was assessed using a 1.0% agarose gel. Thereafter, the quality and quantity of RNA were assessed using a Nano Photometer® spectrophotometer (IMPLEN, CA, USA) and an Agilent 2100 Bioanalyzer (Agilent Technologies, CA, USA). High-quality RNA samples were subsequently submitted to Sangon Biotech (Shanghai) Co., Ltd. for library preparation and sequencing.

### Library construction and sequencing

For miRNA sequencing, libraries were generated using the NEBNext® Multiplex Small RNA Library Prep Set for Illumina® (NEB, USA) according to the manufacturer's recommendations. Using total RNA as the starting sample, small RNA ends were directly connected to the adapter and synthesized by reverse transcription into cDNA. DNA fragments of 140–150 bp were separated by PAGE gel electrophoresis and the cDNA library was recovered. Finally, library quality was assessed on the Agilent Bioanalyzer 2100 system, and the libraries were sequenced on an Illumina NextSeq 500 platform.

For RNA-seq analysis, sequencing libraries were generated using the NEBNext® Ultra™ RNA Library Prep Kit for Illumina® (NEB, USA) according to the manufacturer's recommendations. Sequencing was performed using the Illumina Novaseq 6000 platform, generating 150 bp paired-end reads. The Illumina sequencing data used in this study could be found in Sequence Read Archive data base with project accession number: PRJNA832521.

### Analysis of differentially expressed mRNA

Paired-end clean reads were aligned to our newly generated reference genome of the *E. tenella* H strain (deposited into CNGB Sequence Archive of China National GeneBank DataBase with accession number CNP0003153) using hisat2 software version 2.2.1 (23). Htseq-count version 0.13.5 (24) was used to count the reads. Differential expression analysis was performed for both conditions (Comparison of endogenous and exogenous developmental stages, and comparison between USO and SO) using the DESeq2 R package (1.20.0) (25). *P*-values were adjusted using the Benjamini & Hochberg method. Corrected *P* < 0.01 and log2 (Fold change) > 1 were considered significantly different. The FPKM values of each gene were calculated as described previously (26), and the FPKM values of the selected genes were used for clustered heatmap drawing *via* Pretty heatmaps in the R package.

### Known miRNAs and novel miRNAs prediction

Clean reads were obtained by removing reads that contained ploy-N, with 5′ adapter contaminants, without 3′ adapter or the insert tag, reads that contained ploy A or T or G or C, and low-quality reads from raw data. Then, a certain range of lengths was chosen from the clean reads to perform all downstream analyses. Using Bowtie software (27), clean reads were searched against several databases such as Silva, GtRNAdb, Rfam, and Repbase to filter for ribosomal RNA (rRNA), transfer RNA (tRNA), small nuclear RNA (snRNA), small nucleolar RNA (snoRNA) and other ncRNAs and repeats. The remaining reads were used to detect known miRNAs and novel miRNAs by comparison with our reference genome and known miRNAs from miRBase. Randfold software (28) was used for secondary structure prediction. Target gene prediction was performed by psRobot_tar in miRanda (29). Gene function was annotated based on the following databases: Nr, Pfam, KOG/COG, Swiss-Prot, KEGG, and GO.

### Differentially expressed miRNA detection and target gene enrichment

DESeq2 (25) was used to compare expression levels between sample pairs. We estimated library size correction factors with median of ratios method, based on the number of mapped reads, in DESeq2 and used those factors in further models analyzing differential abundance. We used fold change > 2 and *P* < 0.05 as thresholds to define a significant differentially expressed miRNA. To provide an overview of the different states of gene expression, we used volcano plots to present genes with differential expression between paired samples. Gene Ontology (GO) enrichment analysis was used on target gene candidates of differentially expressed miRNAs. Goseq (30) was implemented for GO enrichment analysis. Transcripts per million (TPM) were calculated for each miRNA for data normalization (TPM = Readcounts * 1,000,000/Mapped Reads), and used for clustered heatmap drawing.

### Identification of miRNA biogenesis-related proteins

Key domains are involved in miRNA biogenesis, including Piwi-Argonaute-Zwille (PAZ, Pfam accession number: PF02170), Piwi (Pfam accession number: PF02171), Ribonuclease 3 (RNase III, Pfam accession number: PF00636), double-stranded RNA binding motif (Pfam accession number: PF00035) and double stranded RNA binding domain (DSRM, Pfam accession number: PF03368), whose models were obtained from the Pfam database (http://pfam.sanger.ac.uk/). Local HMMER3 (31) was used to find protein sequences containing domains targeting *E. tenella* and several other species by default parameters. Multiple alignments and maximum likelihood phylogenetic trees were built by muscle v3.8 (32) and IQ-TREE2 (33) based on Dicer and Argonaut sequences, respectively. A thousand bootstrap replicates were computed for each phylogenetic tree.

## Results

### Core components of the RNA silencing machinery are presented in *E. tenella*

**Figure 1:**
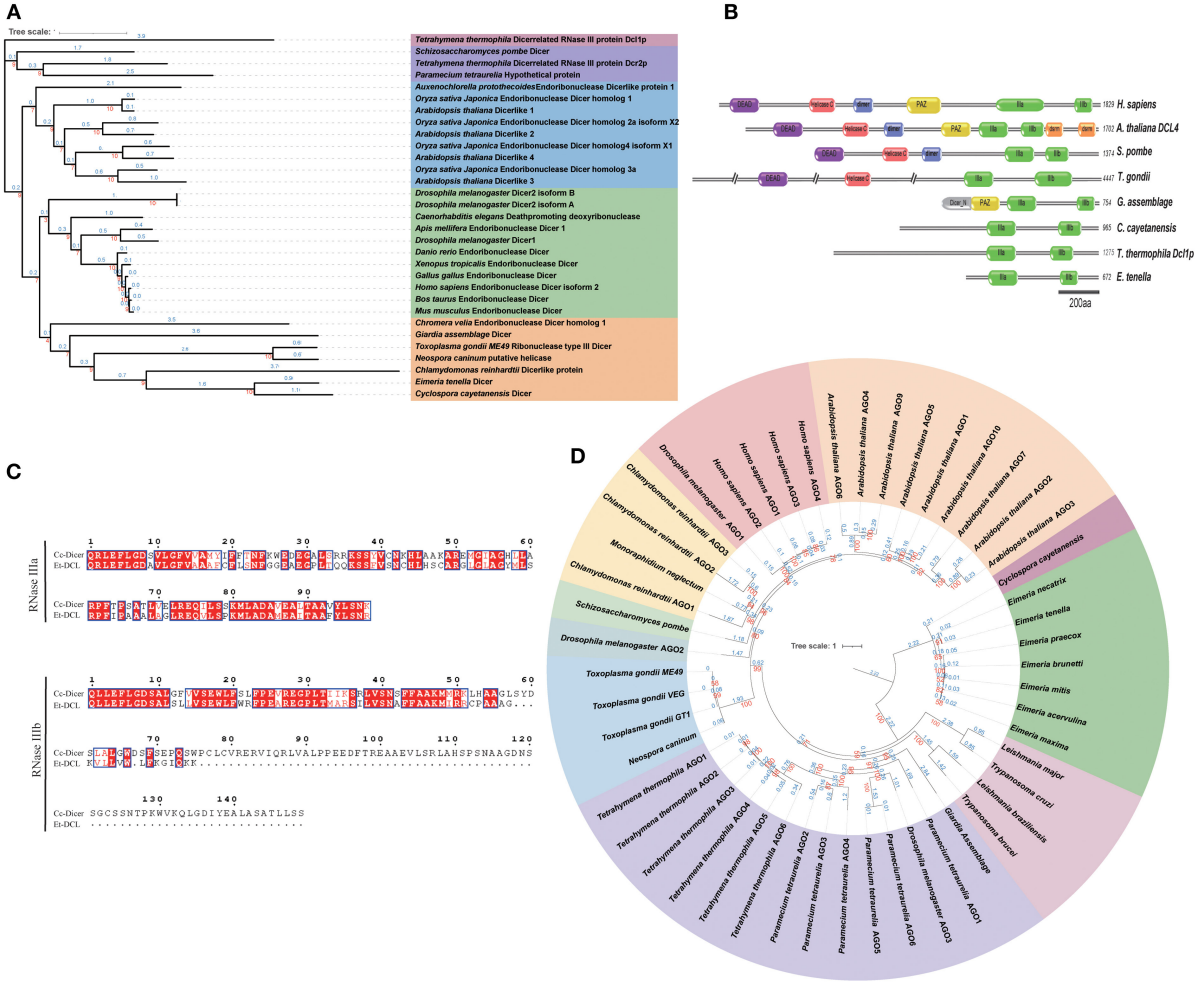
Domain organization and phylogenetic analysis of Dicer and Ago proteins. **(A)** Dicer-based maximum likelihood tree showing the evolutionary relationships between *Eimeria* and other species. Protein sequences were aligned by Muscle software, and the unrooted maximum likelihood tree was constructed using IQ-TREE2 with 1,000 bootstrap replicates. **(B)** Domain organization of Dicer proteins. IIIa, RNase IIIa; IIIb, RNase IIIb; PAZ, Piwi-Argonaute-Zwille domain; dsrm, recognizable dsRNA binding domain; Helicase C, RNA helicase domain; DEAD, DEAD box helicase; dimer, Dicer dimerisation domain. **(C)** Sequence alignment of conserved RNase domains of Dicer proteins from *E. tenella* and *C. cayetanensis*. **(D)** Argonaute-based maximum likelihood tree (rooted at midpoint) showing the evolutionary relationships between *Eimeria* and other species. Red and blue numbers on both phylogenetic trees represent the bootstrap values (%) and branch lengths, respectively. Protein accession ID shown in Supplementary Datasets 10, 11.

Previous analyses have shown that plant and animal Dicer proteins have divergent origins (34). Sequence analysis revealed that the *E. tenella* genome encodes only a Dicer-like protein, Et-ECL, which exhibits significant variability in protein sequence and domain organization compared to higher eukaryotes (Figure 1A and Supplementary Dataset 1). Et-ECL has two RNase III catalytic domains (RNase IIIa and RNase IIIb), but lacks a recognizable DSRM, PAZ, or RNA helicase domain (Figure 1B). These features clearly distinguish Et-DCL from Dicer of another coccidian, *T. gondii*, which has 4,447 amino acids and contains an additional helicase domain and a DEXDc domain (19). This structure is similar to the Dicer-like protein of the Sporozoa parasite *Cyclospora cayetanensis* (Figures 1B,C) and belongs to a specific branch supported by a strong bootstrap score (Figure 1A). Dicers from fungi, plants and vertebrates show similarity in domain organization and are located in their own clade in the phylogenetic tree. However, we were unable to identify any Et-ECL orthologs in other chicken coccidia even under a high cutoff (*E*-value = 1.0), and we speculate that this may be due to the lack of genome completeness or the absence of RNA silencing machinery in these species (Supplementary Dataset 2).

Interestingly, despite the lack of Dicer-like proteins in other chicken coccidia, a single Argonaute protein (Ago) was found in each *Eimeria* parasite by HMMER searches using the PAZ domain (PF02170) and the Piwi domain (PF02171) (Supplementary Dataset 1). By phylogenetic analysis, the Ago protein of *E. tenella* is located in the same branch with several other species of *Eimeria* and *Cyclospora* with long branch length. *T. gondii* and *N. caninum* Agos fall in a clade with algae (*Chlamydomonas* and *Monoraphidium*), plants and animals (Figure 1D). These results suggest that *Eimreia's* Ago proteins are divergent from Tg-Ago and others.

### Characterization of miRNAs in *E. tenella* by illumina sequencing

As Dicer-like protein was only found in *E. tenella* among chicken coccidia, we are interested to know its characteristics and roles in gene regulation during different life stages. To this end, miRNAs from USO, SO, Mer108, and Mer120 were identified by Illumina sequencing. After removal of low-quality reads, a total of 58 million clean reads were obtained (Supplementary Dataset 3). Unannotated reads containing miRNAs were obtained after filtering for ncRNAs such as ribosomal RNA (rRNA), transfer RNA (tRNA), intranuclear small RNA (snRNA), nucleolar small RNA (snoRNA), and repetitive sequences (the proportions of small ncRNAs in the four samples are given in Supplementary Dataset 4). In the merozoite samples (Mer108 and Mer120), only ~23% of the unannotated reads could be aligned with the *E. tenella* genome, and ~50% in the USO and SO samples (Supplementary Dataset 5). Comparable reads in each sample (ranged from 0.7 to 1.3 M, and with an average of 0.76 M for Mer108, 1.1 M for Mer120, 1.23 M for SO, and 0.83 M for USO) were used for differential expression analysis.

**Figure 2:**
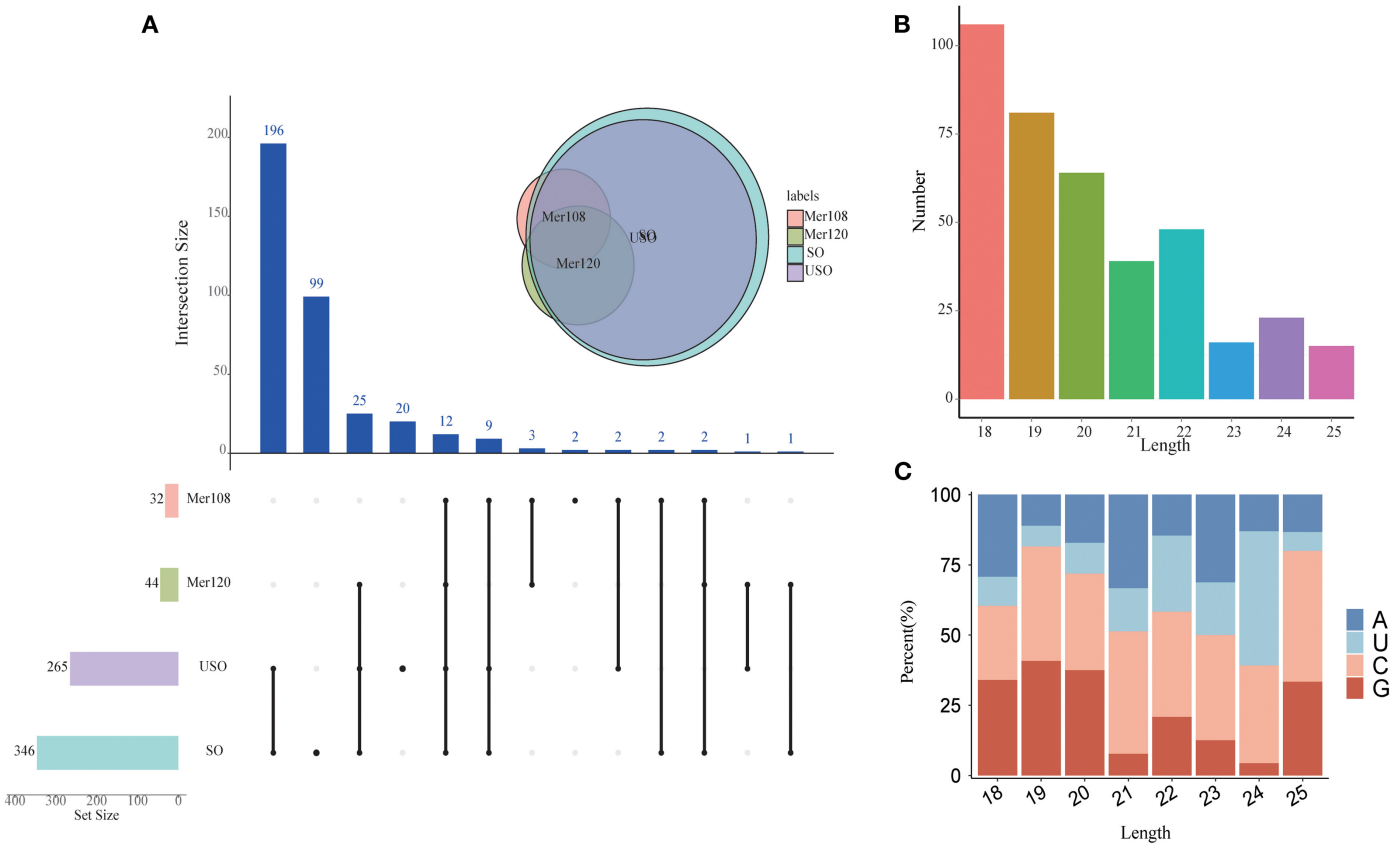
Characterization of the novel miRNAs in *E. tenella*. **(A)** An UpSet plot showing the set of all miRNAs from four different developmental stages. Vertical bars show the number of intersecting miRNAs between stages, denoted by the connected black circles below the histogram. The horizontal bars show the size of the transcript sets between stages. The Venn diagram shows the numbers and overlapping situation of miRNAs with different transcripts in different groups. **(B)** Length distribution of the novel *E. tenella* miRNAs. **(C)** Codon usage preference of the first base of *E. tenella* miRNAs of different lengths.

In total, we obtained 392 miRNAs with length distributions ranging from 18 to 25 nt across all samples, of which 18 nt represents the major size class (Figures 2A,B and Supplementary Dataset 6), which is significantly different from the length distribution and major size of *T. gondii* miRNAs (ranging from 17 to 33 nt with a major size of 25 nt). The development of miRNAs from precursors to mature bodies was accomplished by Dicer enzyme cutting. The specificity of enzyme cutting points gives a strong bias to the first base of its mature body sequence. By base-preference of the first base at the 5' end and at each site of the miRNA, we found that 18–20 nt of the miRNA first bases were biased toward G, except for miRNAs of 24 nt length that were biased toward U and others that were biased toward C (Figure 2C). This finding is less consistent with the previously reported first base bias toward the use of U (35–37), which is speculated to be either species-specific or related to the quality of sequencing, for reasons that require further investigation.

### Differential miRNA expression between endogenous and exogenous development

**Figure 3:**
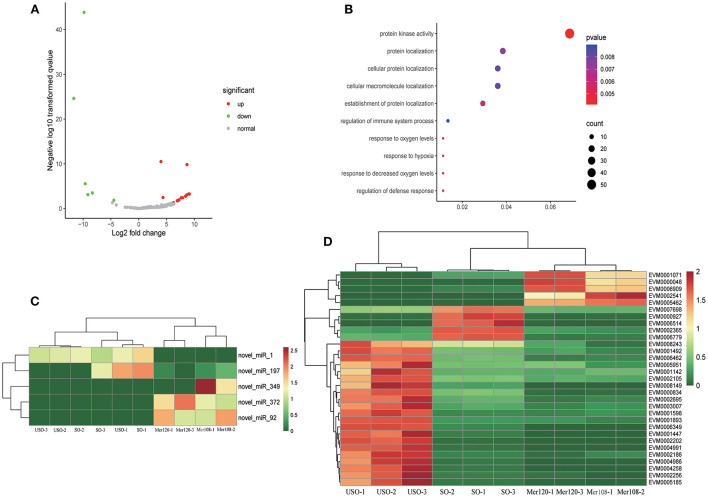
Target gene analysis of differentially expressed miRNAs at exogenous and endogenous stages. **(A)** Volcano plot showing differentially expressed miRNAs between endogenous developmental stages (Mer108 and Mer120) and exogenous stages (USO and SO). Gray dots represent non-differentially expressed miRNAs, red dots represent up-regulated miRNAs, and green dots represent down-regulated miRNAs. |log2 (FC) | ≥ 1.00 and FDR ≤ 0.05 were regarded as statistically significant. **(B)** Go enrichment plot of differentially expressed miRNA-targeted genes in exogenous and endogenous stages. Only the 10 GO functions with the smallest *p*-value were taken for statistical purposes. **(C)** Heatmap showing differentially expressed miRNA-targeted genes enriched in the protein kinase activity pathway. **(D)** Heatmap showing differentially expressed miRNAs enriched in the protein kinase activity pathway.

*Eimeria* parasites undergo endogenous and exogenous development, interacting with totally different environments. To understand whether miRNAs have a regulatory effect on the parasite when dealing with different environments, we compared miRNA expression between endogenous (Mer108 and Mer120) and exogenous (USO and SO) developmental stages. A total of 24 differential miRNAs were identified, six of which were up-regulated and 18 were down-regulated (Figure 3A). In addition, two miRNAs were unique at the endogenous stage and 124 were unique at the exogenous developmental stage. We found that the target genes of differential miRNAs were mainly enriched for protein kinase activity (GO: 0004672, *p* = 0.0041), including CDPKs, CMGCs and ribosomal protein S6 kinase, which were up-regulated at the endogenous schizogony stages (Figures 3B–D and Supplementary Dataset 7). Apart from the protein kinases, protein translation related proteins (mainly ribosomal proteins) were also significantly upregulated at schizogony stages (Supplementary Dataset 8). CDPKs participate in many processes, such as parasite invasion and egress from host cells (38). Therefore, we speculate that these miRNAs may be involved in the parasitism of *E. tenella* during schizogony stage.

### Differential miRNA expression during sporulation

*Eimeria* parasites undergo drastic chromatin rearrangements during the sporulation process though meiosis and mitosis, leading to dramatic changes in gene expression through complex epigenetic reprogramming events, as well as variations in cellular morphogenesis (39). Since miRNAs play a key role during development, it is reasonable to assume that they could play a relevant role in sporulation.

**Figure 4:**
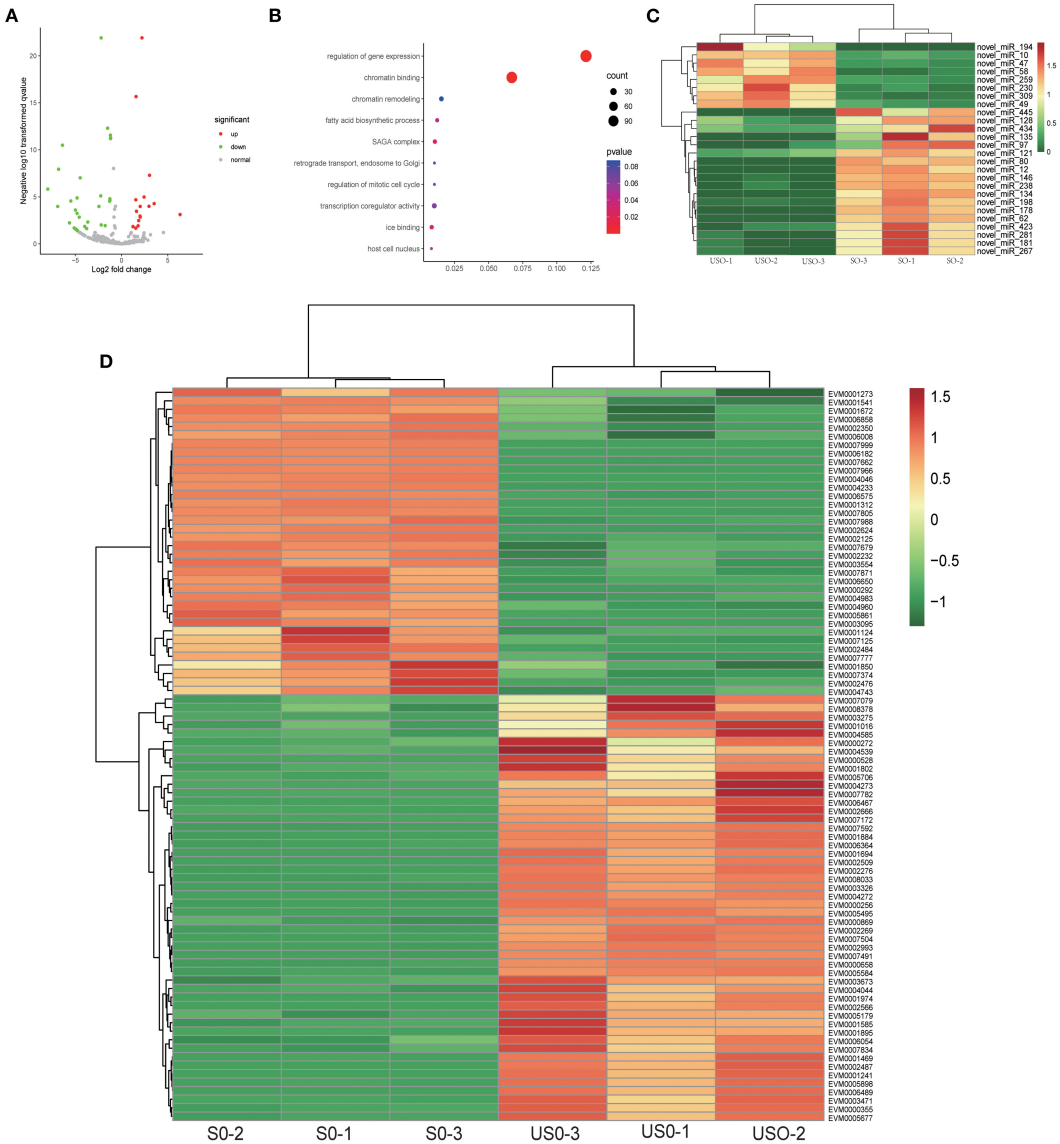
Target gene analysis of differentially expressed miRNAs in USO vs. SO. **(A)** Volcano plot showing differentially expressed miRNAs between USO (unsporulated oocysts) and SO (sporulated oocysts). **(B)** Go enrichment plot of target genes for differentially expressed miRNAs in USO vs. SO. Only the 10 GO functions with the smallest *p*-value were taken for statistical purposes. **(C)** Heatmap showing DEGs (differentially expressed genes of targets) of differentially expressed miRNAs enriched in gene expression regulation and chromatin binding pathway. **(D)** Heatmap showing differentially expressed miRNAs enriched in gene expression regulation and chromatin binding pathway.

In the comparison between USO and SO, we obtained 56 differentially expressed miRNAs, of which 38 were up-regulated and 18 were down-regulated (Figure 4A). The 56 differentially expressed miRNAs targeted 3,442 differentially expressed mRNAs. By GO enrichment analysis, we found that most of the target genes (86 genes) were enriched in the regulation of gene expression (GO: 0010468, *p* = 8.77E−10) and chromatin binding (GO: 0003682, *p* = 1.74E−5) pathways (Figure 4B). After clustering all differentially expressed target genes enriched in this pathway (Figure 4C and Supplementary Dataset 9), it was observed that genes highly expressed in USO were mainly associated with gene expression regulation (Figure 4D). For example, the chromo domain-containing protein (EVM0000869) is associated with epigenetic chromatin remodeling and operation (40), while ApiAp2 transcription factors (e.g., EVM0006489) are reported as master regulators in many cellular processes. This result reveals a comprehensive chromatin remodeling and gene expression regulation during sporulation in *Eimeria*, and the involvement of these miRNAs in this process.

### miRNA-mRNA interaction

**Figure 5:**
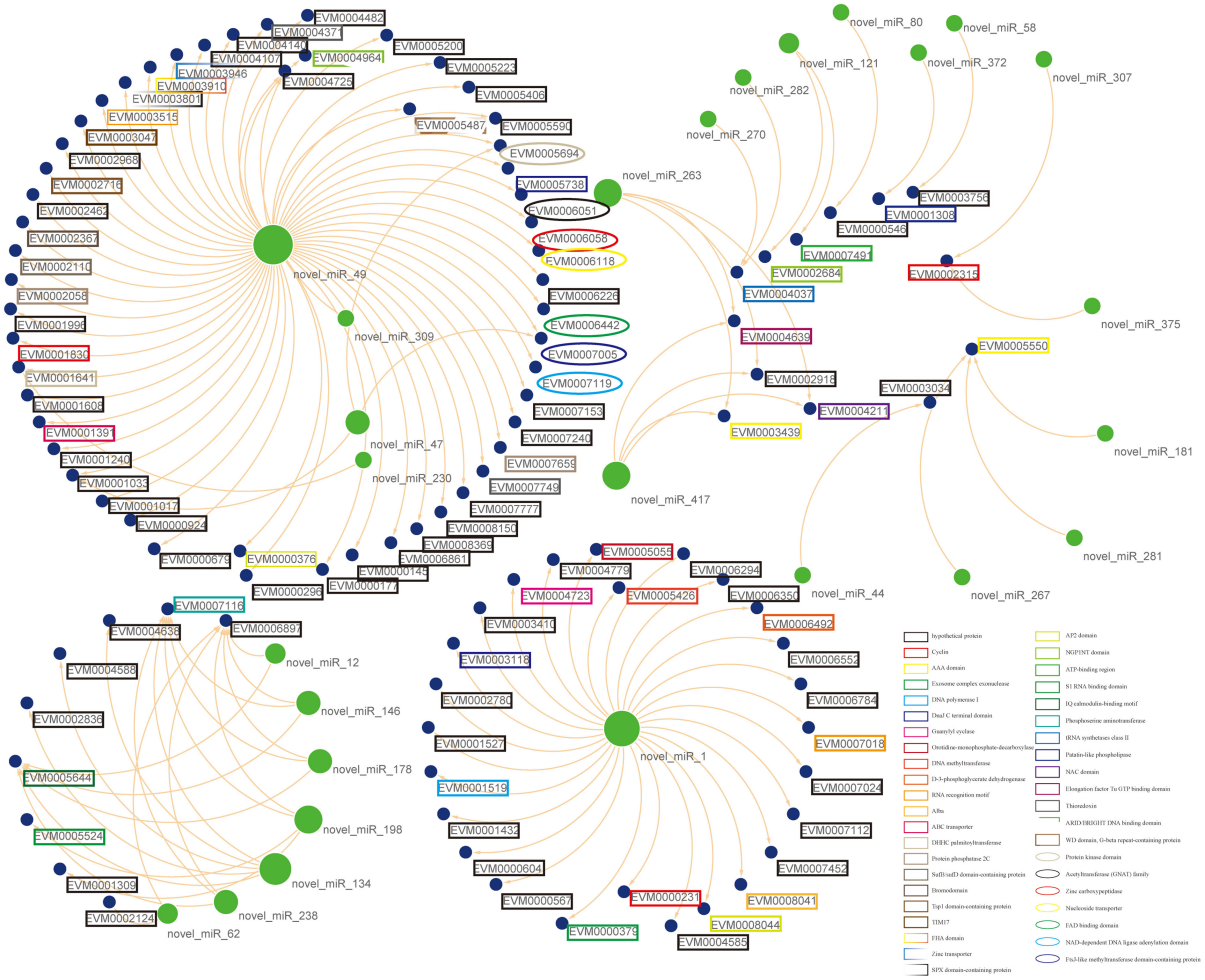
Putative functional interactions. All the co-expressed interactions found in this study are shown as a network by using Cytoscape. The size of the node represents its outdegree, and the functional annotations for mRNAs were also marked in the lable.

Numerous reports have found that miRNAs lead to translational repression or mRNA degradation through complementary binding to the 3' UTR of target genes. In animals, most miRNAs are partially complementary to target mRNA sequences and usually repress translation of target genes, implying that miRNA expression is negatively correlated with mRNA expression (41, 42). Based on the regulation of negative regulation by miRNAs in animals, it is possible to predict the target genes that are potentially negatively regulated by miRNAs by combining miRNA target gene data and transcriptome data, greatly narrowing down the number of crude genes underlying differential miRNAs. By miRNA-mRNA association analysis, twenty-six differential miRNAs were obtained to be negatively correlated with 101 genes (Figure 5). However, not all differential miRNAs had negatively correlated mRNAs. It is speculated that it may be related to the positive regulation of mRNAs, but the mechanism of this positive regulation remains unclear (43). This suggests that the manipulation of miRNAs on target genes needs to be verified by a large number of experiments.

## Discussion

miRNAs are considered key regulators of gene expression at the post-transcriptional level. Previous studies have found that *T. gondii* has a complete RNA silencing pathway, suggesting that small non-coding RNAs may play a key role in parasite development and its parasitism in the host (19, 44). In this study, we characterized the RNA silencing mechanism of *E. tenella*, and also investigated the distribution of miRNA population at different periods of the life cycle.

We identified Dicer-like protein and Argonaute protein in the genome of *E. tenella*, suggesting the potential of a complete RNA silencing machinery. However, we did not find the Dicer homolog in other chicken coccidia, but instead found the Argonaute protein. It is speculated that this may be due to the lack of genome completeness or the loss of RISC-related genes in a similar evolutionary process as in *Leishmania* species, in which *L. braziliensis* do exist RISC proteins and others (e.g., *L. major* and *L. donovani*) do not (16, 34). By phylogenetic analysis, we found that the Et-ECL and Et-Ago are not, as in *T. gondii*, patched together by proteins of plant and fungal origin. Interestingly, Et-ECL has only two RNase domains, which suggests that it may function like *Tetrahymena thermophila*, requiring coupling of other proteins for pre-miRNA clipping (8).

By high-throughput sequencing, we identified a total of 392 miRNAs, and there were far more miRNAs in sporulated and unsporulated oocysts than in merozoites. We checked the purity after harvesting the merozoites and concluded that the final sample had over 90% merozoites except for some cell fragments (data not shown). Since chickens are abundant in miRNAs and *Eimeria* are relatively poor in miRNAs, therefore, a small amount of host cell contamination may result in a smaller number of *Eimeria* miRNA reads. However, comparable mapped reads in each sample were obtained and used for miRNA prediction and further analysis (Supplementary Dataset 5). Thus, we believe the different miRNA numbers in the different life stage samples should be its natural characteristics. Through the first base bias analysis, we found that the first base of the remaining miRNAs was not U, except for miRNAs with a length of 24 nt. Whereas, in other eukaryotes, such as *Drosophila, Penicillium marneffei*, Swarnaprabha rice and Rat, there is a high proportion of miRNAs whose first base is U (35–37, 45–47). Considering the cutting point preference of Dicer on the first base of miRNA, this different characteristics may result in significant differences in miRNAs of *E. tenella* compared to other organisms (48).

Previous studies reported that 336 miRNAs and 201 miRNAs were found in the tachyzoites of *T. gondii* RH strain and ME49 strain (44), respectively, while 300 miRNAs were found in the tachyzoites of *N. caninum* (49). More than 200 miRNAs were found in the green alga *C. reinhardtii* (50–52) and 148 miRNAs were found in *Drosophila melanogaster* (53). These results show that the number of miRNAs seems to be independent of the evolution of the species. By analyzing the differential genes and miRNAs, we found that miRNAs may be involved in the regulation of coccidia growth and development, which is consistent with the findings in animals and plants (54, 55).

By comparing endogenous and exogenous development stages, we found that the target genes of differential miRNAs were mainly enriched for protein kinase activity. EVM0005185 and EVM0001071 annotated as *P. cynomolgi* circumsporozoite protein and CMGC kinase, respectively, are both highly expressed at endogenous developmental stages. The former gene associated with cell movement (56) and the latter related to cell division (38). These are both crucial processes for merozoites development *in vivo*. Meanwhile, EVM0002541, annotated as CDPK1, was highly expressed in the endogenous stage. Previous report on *T. gondii* showed that CDPK1 has an important role in regulating parasite motility and host cell invasion (38). Thus, we speculate that these differentially expressed miRNAs may be involved in parasite invasion or egress by regulating its target genes. In addition, we found only one differentially expressed miRNA (novel_miR_400) in the comparison between two merozoite stages, suggesting relatively consistency of miRNA expression in merozoites. However, the target mRNA of the miRNA was not differentially expressed (data not shown).

The target gene functions of the differentially expressed miRNAs in USO and SO are significantly enriched in two pathways, namely gene expression regulation and chromatin binding, which are consistent with the biological process of unsporulated oocysts undergoing meiosis and mitosis to give rise to eight haploid sporozoites. EVM0000869, annotated as a Chromo domain-containing protein, is evolutionarily conserved and plays an important role in regulating gene activation, genome recombination and repair, and chromatin remodeling in different organisms. These proteins regulate epigenetic processes through various signaling pathways (40). The gene is also targeted by three differentially expressed miRNAs (two down-regulated and one up-regulated), making it difficult to find the specific functions of these miRNAs. Interestingly, the target gene EVM0007966 had only one differentially expressed miRNA, namely novel_miR_423. There was a positive correlation between them. During the whole life cycle, EVM0007966 had the lowest expression in the unsporulated oocysts, while all miRNAs of the target gene had the highest expression in sporulated and unsporulated oocysts, and no miRNA of the target gene was present in the merozoite. This also suggests that the regulatory mechanism of miRNAs is complex and that the same miRNA may play different roles at different times. Further work is needed to better understand the exact roles of differentially expressed genes, miRNAs, and miRNA interactions at different developmental stages.

## Conclusion

In this study, we characterized the RNA silencing machinery in *E. tenella* and investigated the miRNA distribution of *E. tenella* at different time periods by high-throughput sequencing. We identified 58 miRNAs that were differentially expressed between US and SO and potentially regulate Ap2 transcription factors and epigenetic modulators. Twenty-four miRNAs were differentially expressed between endogenous and exogenous development stages, and may be involved in protein kinase activities. Our results might lead to functional understanding of gene regulation in *E. tenella*, and also has the potential to provide basic information to inform research on treatment against the parasite.

## Data availability statement

The datasets presented in this study can be found in online repositories. The names of the repository/repositories and accession number(s) can be found in the article/supplementary material.

## Ethics statement

The animal study was reviewed and approved by Administration Committee of Laboratory Animals in Guangxi University (Approval No: Gxu-2021-013).

## Author contributions

LZ: investigation, writing—original draft, and visualization. LC: investigation. HZ: validation and visualization. HS: supervision. XL: writing—review and editing. XS: conceptualization and writing—review and editing. DH: software, funding acquisition, and supervision. All authors read and approved the final manuscript.

## Funding

This work was supported by the National Natural Science Foundation of China (Grant No. 32102694), the Natural Science Foundation of Guangxi Zhuang Autonomous region (Grant No. 2021GXNSFBA220057), and the Specific Research Project of Guangxi for Research Base and Talents (Grant No. AD21075028).

## Conflict of interest

The authors declare that the research was conducted in the absence of any commercial or financial relationships that could be construed as a potential conflict of interest.

## Publisher's note

All claims expressed in this article are solely those of the authors and do not necessarily represent those of their affiliated organizations, or those of the publisher, the editors and the reviewers. Any product that may be evaluated in this article, or claim that may be made by its manufacturer, is not guaranteed or endorsed by the publisher.

## Supplementary material

Supplementary Dataset 1 HMMER search results for key RNAi pathway proteins in *Eimeria* species. Thirteen protozoan reference genomes were downloaded from vEuPathDB (release 57), and new assemblies of *E. tenella, E. maxima* and *E. acervulina* from our unpublished data were built as a database. Hmm model of PAZ, Piwi, Ribonuclease 3, double-stranded RNA binding motif and double stranded RNA binding domain were downloaded from Pfam database and used as query against the protozoan genome database by hmmsearch using default parameters.

Supplementary Dataset 2 Sequence alignment files of Dicer and Ago proteins. Amino acid sequences were trimmed manually and then were aligned by Muscle v3.8 with default parameters.

Supplementary Dataset 3 Statistical table of sequencing data.

Supplementary Dataset 4 SRNA annotation classification statistics for different samples.

Supplementary Dataset 5 Statistical table of comparative information with the reference genome.

Supplementary Dataset 6 Statistics of the amount of miRNA expression in each sample, and normalization of the amount of expression by TPM.

Supplementary Dataset 7 Genes targeted by differentially expressed miRNAs enriched in the protein kinase activity pathway.

Supplementary Dataset 8 Table of differential mRNA statistics for differential miRNA targets in endogenous and exogenous stages.

Supplementary Dataset 9 DEGs (differentially expressed genes for targets) of differentially expressed miRNAs enriched in gene expression regulation and chromatin binding pathway.

Supplementary Dataset 10 Protein accession ID of Dicer proteins in different species.

Supplementary Dataset 11 Protein accession ID of AGO proteins in different species.
