# Supplementary material for: A comparative study of microRNAs in different stages of Eimeria tenella
Source: Front Vet Sci. 2022 Jul 22;9:954725. doi: 10.3389/fvets.2022.954725 (PMC9353057; doi:10.3389/fvets.2022.954725)
Supplement: Supplementary file 2 [file Data_Sheet_2.docx]

Ago proteins in afa format:

>Cyclospora_cayetanensis_cyc_06383

----------------------------------RHDRKLELGCLEDHLR---DMDHQKA

QITYRNRLCVAVHKGNIYR----------IINVTD-TTADRATFEKGGQTIN--------

--------------------------VAQYFKDAYNEHVEGKLVVQAKEVRGG------S

RVCFLPVNLLRVIAQAPPPTAGSRAVLRPKSAQET---LVDC--A---------------

---ELVADILRT------------------------------------------------

--PAVRNQMSELKLQ------------------IGDKGLPLDVQQNVTNLRKLMGD----

------VRSFGEARIS--WGATERERQQDTSADLS----------PDFRAR----LCQNL

--------VL-RHKVPLDK------QWM-LLVCT-------------------------G

RREHGGSRAAEA---IKN------------------TIELTRQRIRYKNQLVVEME----

-----------APVCCEHFVK----------TEAEWKRGKV-------------EVTKRS

LRDLFS---------LHRQGLRGAIVLLAYDS-D--EVNNRVRIAAKQVSVQMDFPVQCC

KLQTLQNFKNKPEDKSMNMW-WNVYLQFQTKIT-------DEESRAGVPWAADQPLAPLR

EAPVKMSSTQPSQAPPTKVSEIARAIGIAVNSFG--------------------------

---------------------------PASSRCAVVGIVG----------GRNVEHTRFI

SHIGAS--------------DPPNY----------RA----GVIKEVLAHE--VAAIRDV

IEADLGKIEFSSE-------KRELTIEHPVAAAFPEGVFQCALRMCRETDLEAF-SGKAE

DIGAHIN----------------YKGAI---LSCDGPTLTSQGRNILRVIQAEDKL----

--------GV--------------------VLPDTHRLLSY--ARSNYRWLQPIKLTFTA

TADEPTPHEANGVIIALDGYVSPEVKFHKNEP------RLGSTVYLRKAIISHIIQRVHL

--NVLVNPHRPASPMRFFMRG----RDGVQ------------------------------

---GLPVGSYIDSGPIIPISPDTATLEKKFLIGTADPTRG-SPSASEF------HIIANE

SDWSRH-----FLAVVSYKLCHMYYNWAGTVKHPHILQMALAIVRQHQI--YIASESGYG

LDSEFNLDEPAEEQPL-EQQESPEEGEEAETRERLQKRRQEGGYATH-VGGREGLERTDD

AAEREAGKQNLRELKANLERPDSVLMTTAFML----------------------------

------

>AGO_Eimeria_tenella_EVM0002661

----------------------------------RHDRKLEPGLLEDRLR---EMDYQTA

RNKYRNRLCVAVHKGNIYR----------IVEVTD-TTADRATFEKGGATIS--------

--------------------------VAQYFKDMYNQNVDGKQVVTTKEARGG------S

RLCYLPVNLLRVIAQAPQATGSCRPILRPKSAQET---LIEC--T---------------

---ELMNSIIRM------------------------------------------------

--PAVREQMAELKLRI-----------------GGDSGLPVDIQKNITSLKRLIET----

------SPSFGEPRIS--WGAKEKEHQQDTYVDLS----------PDFRAR----LCNNL

--------VL-RHKVPLSG------RWM-LVVCT-------------------------G

RREHGSSRAVET---IKN------------------TIELTRQRIRYKNQVAVDME----

-----------APACCEHFIR----------NEQDWKKGRV-------------ERTKRS

LRELFS---------ENKQYLRGAVVLLAYDS-D--EVNNRVRVAAKQVSLQLDFPIQCC

KLQTLQSFKNRPDDKSMNMW-WNVYLQFQTKIT-------DDSNLAGVPWAADQLLAPLS

E--------GIK-----KVSEVVRVIGIAVNSFG--------------------------

----PPNARCGVVGIVGGTNVEHTQFVSHIGASSPHYYRA----------GVVRDMARHF

KLFWEKLNLRFGDGRIR---RAPVHLLIYRSSPS-GI----SQIRDVLAHE--VGAIRRV

IDADLGKIEFSPE-------TRELTVEHAMAAAFPDAEFAVTVSVCSDSAMRRF-AGKAE

ALGQYAL---------PDAQTLSCPRAI---PDSEGRYILRFKLPEDTV-PLYRPL----

----GFIVGIVTFPEPEKFQFERREDEEGITLPEDHPILVY--ARANSRWMIKTTLLFSA

SADSS--HEFGASIVSVDGEVAAEVNIVGSGLNP------GDIIYLKKATVHQIFQRVFI

--NVLVNPHRPANPIKFFMKG----REGVQ------------------------------

---GLPVGSYIDSGPITPVLPNTETLEEKFLIGTADPTRG-SPSASEF------HIVQNE

SDWSKQ-----YLAILSYKMCHMYYNWAGTVKHPHLLQMALAIVRQHQI--YVA------

------------------------------------------------------------

------------------------------------------------------------

------

>Eimeria_necatrix_ENH_00062980

----------------------------------RHDRKLEPGLLEDRLR---EMDYQTA

RIKYRNRLCVAVHKGNIYR----------IVEVTD-TTADRATFEKGGATIS--------

--------------------------VAQYFKDVYNQNVDGKQVVTTKEARGG------S

RLCYLPANLLRVIAQAPQAIGSCRPIMRPKSAQET---LIEC--T---------------

---ELMNSILRM------------------------------------------------

--PAVREQMAELKLRI-----------------GDDSGLPVDIQENITSLKRLIEN----

------SPSFGEPRIS--WGAKEKEHQQDTYVDLS----------PDFRAR----LCNNL

--------VL-RHKVPLSG------RWM-LVVCT-------------------------G

RREHGSSRAVET---INN------------------TIELTRQRIRYKNQLVVDME----

-----------APACCEHFVR----------SEQDWKKGRV-------------ERTKRS

LRELFS---------ENRQYLRGAIVLLAYDS-D--EVNNRVRVAAKQF-----------

----------------------------QTKIT-------DDSNHAGVPWAADQLLAPLS

E-------------GIKKVSEVVRVIGIAVNSFG--------------------------

----PPNARCGVVGIVGGTNIEHTQFVSHIGASSPHNYRA----------GVIRDMARHF

KLFWEKLNLRFGDGKIR---RAPVHLLIYRSSPS-GI----SQIRDVLAHE--VGAIRRV

VDADLGKIEFSPE-------TCELTVEHAMAAAFPDAEFAVTVSVCSGSAMRRF-AGKAE

ALGQYAL---------PEAQTLSCPRAI---PDSEGRYILRFKLPEDMV-PLYRPL----

----GFIVGIVTFPEPQKFQFERREDEEGITLPEDHPILVY--ARANSRWMIKTTLLFSA

SADGS--NEFGASIVSVDGEVAAEVKIVGSGLNP------GDIIYLKKATVHQIFQRVFI

--NVLVNPHRPANPIKFFMKG----REGVQ------------------------------

---GLPVGSYIDSGPITPVLPNTETLEEKFLIGTADPTRG-SPSASEF------HIVQNE

SDWSKQ-----YLAVLSYKMCHMYYNWAGTVKHPHLLQMALAIVRQHQI--YVASESGFG

LESEFALEEKLEAKPVADASESPEEGEEQQLRQRVQRRKQEVGYAA--VGLHHAHREALE

AEERDAGLENLKELKANLENKQSRLMTTAFML----------------------------

------

>Eimeria_brunetti_EBH_0070680

----------------------------------RHDRKLEVGFLEDRLR---EMDYQQA

QFTYKNRLCVTVHKGNLYR----------IVDITD-TTADRATFEKGGATIT--------

--------------------------VAQYFKDAYNENVVGKQVVMAREARGG------S

RVCYLPANLLRVVAQAPQPIGGSRAMLRPKSAQET---LVEC--T---------------

---ELVKDILRT------------------------------------------------

--PAVKEQMAELKVHI-----------------GANGGLPVDVHKDITSLRTVMGD----

------ELRYGEPRIS--WGPERKEEQQDTFVELT----------PDFRAR----LCQHL

--------VL-RHKVPLNC------HWM-LVVCT-------------------------G

RREHGSSRAVES---IKN------------------TIELTRQRIRHRGQLTVDMD----

-----------APTCCEHFIR----------SEADWKKGRV-------------ERTKRS

LGDLFI---------KHRHGLRGAVVLLAYDS-D--DTNNRVRVAAKQVSIQQDFPIQCC

KLQTLQAFKSRPEDKSMNMW-WNVYLQFQTKIT-------DDSLRAGIPWATDQLLAPIK

E-----------GVRPPQISEVVRVLGIAVNSFG--------------------------

----PANARCGVVGIVGGRNVAHTQFMSHIGASAPPNYRA----------GVIKDMARHF

KPFWEKLTFEETTKPHK---RAPAHLLIYRSAPS-GT----SQIKEVIAQE--VTAIRSV

IAADLGNFEFLPE-------TSELVVEHPMAAAFPDAEFALVLAVCSAAELRRA-AGKGE

EVGQLAR---------PRPETLSCQGTT---VGSEGRRVLKFKLKQETV-PLLQST----

----AILVSEVDAPEPEAFQVEEGEDGKRITLPDSHPLLVY--ARANVRWLKNIEMRFTN

TADGPL-NGFKGFIVPVNGEIYGDVNVREGESSPGAESTLAETIYLRQAAISQLVQKVFI

--NLLVNPHRPANPMKFFMKG----REGVQ------------------------------

---GLPVGAYIDSGPVIPVAPNTETPEEKFLIGTADPTRG-SPSASEF------HVAQSK

RHMPLL-----RLFDAV-------------------------------------------

------------------------------------------------------------

------------------------------------------------------------

------

>Eimeria_mitis_EMH_0010570

----------------------------------RHDRKLEAGTLEDHLR---QMDYQQA

QLTYRNRLCVTVHKGNLYR----------IVNITD-TTADRTTFEKGGATIT--------

--------------------------VAQYFKDAYNENVDGKQVITAKEARGG------S

RLCYLPANLLRVIAQVPQPVEGSRAMLRPKSAQET---LLEC--T---------------

---ELVKDILRT------------------------------------------------

--PAVREQMAELKVHI-----------------GDNGGLPIDVHKDITSLRKVMGD----

------ELRYGEPRIS--WGPEKKEEQQDTFVELT----------SDFRAR----LCQHL

--------VL-RHKVPLNS------NWM-LVVCT-------------------------G

RREHGGSRAVES---IKN------------------TIELTRQRIRHRGQLTVDMD----

-----------APAFCEHFIR----------SEADWKRGRV-------------ERTKRS

LRDLFI---------RQRHGLRGAIILLAYDS-D--DTNNRVRVAAKQVSVQQDFPIQCC

KLQTLQAFKSKPEDKSMNMW-WNVYLQFQTKIT-------DDALRAGIPWAADQLLAPIR

E-----------GVRPPQVSEVVRVIGIAVNSFG--------------------------

----PSSARCGVVGIVGGRNAGHTQFVSHIGASAPANYRA----------GVIKDMEKHF

KLFWEKLTFEESTKPQK---HAPVHLLIYRSAPS-GS----SQIKEVLAQE--VTAIKKV

LAADLGNFEFSPE-------TSELIVEHPMATAFPDAEFAIVLTVCPAAEMRRS-AGKAE

EITQHVR---------PRHETLTCQGAT---IGSEGRRTLRFKLQEGKE-PLLQAM----

----AILVSEFDVPEPEEFQVEEGEEGKRITLPDDHPILVY--AKSNSRWLKNIELQFTS

TADGPL-NGFKGVIVPVHGEIYADVNIQEDESSPEAESSIGDTIYLRQAAISQLVQKVFI

--NVLVNPHRPANPMKFFMKG----REGVQ------------------------------

---GLPVGAYIDSGPIIPVAPNTETPEEKFLIGTADPTRG-SPSASEF------HVAQNE

SDWSKH-----YLAVVSYKLCHMYYNWAGTVKHPHLLQMALAIVRQHAI--YIGSESGFG

LESEFTLEQQPEEETAKERPASPELGEEPEIRERVQKRRQEAGQEAP-AAMRVQRETEED

AEERNAGLENLKELKEALENRDSILMTTAFML----------------------------

------

>Eimeria_praecox_EPH_0024580

----------------------------------RHDRKLEAGFLEDRLR---EMDYQRA

QITYRNRLCVTVHKGNIYR----------IIDITD-TTADRATFDKGGATIT--------

--------------------------VAQYFKDAYNENVEGKQVIMAKEARGG------S

RLCYLPVNLLRVIAQAPQPVEGSRAMVRPKSAQET---LVEC--T---------------

---ELVKDILRT------------------------------------------------

--PAVKEQMAELKVHI-----------------GDNGGLPIDVQKDITSLRKVMGE----

------EQRYGEPRIS--WGPEGREEQQDTFVELT----------PDFRAR----LCQHL

--------VL-RHKVPLSS------HWM-LVVCT-------------------------G

RREHGSSRAVES---IKN------------------TIELTRQRIRHRGQLTVDME----

-----------APACCEHFIR----------SEADWKKGRV-------------ERTKRS

LRDLFI---------KQRHGLRGAIILLAYDS-D--DTNNRVRVAAKQVSVQQDFPIQCC

KLQTLQAFKSRPEDKSMNMW-WNVYLQFQTKIT-------DDSLHAGVPWVADQLLAPIR

E-----------GVRPPQISEVVRAIGIAVNSFGACGAKC--------------------

NLLRPPNARCGVVGIVGGRNVAHTQFMSHIGASAPTNYRA----------GVIKDMARHF

KLFWEKLTFEETTKPHK---HAPAHILIYRSAPS-GS----SQIKEVLAQE--VTAIKSV

LGADLGNFEFSPE-------TSELTIEHPMATAFPDAEFAVVLTVCSVDEMRRS-TGKAE

EVSQHSR---------PRHETLTCQGAT---IGSDGRHILKFKLEEGTV-PLLQAM----

----AILVSEFDAPEPEAFQLEEGEDGKRITLPEDHPLLVY--AKSNSRWLKNIELQFTT

TADGPL-DGFKGVIVPVNGEIYADINVRETASSPRTELRAGDTIYLRQAAISQLIQKVFI

--NVLVNPHRPANPMKFFMKG----REGVQ------------------------------

---GLPVGAYIDSGPIIPVAPNTEMPEEKFLIGTADPTRG-SPSASEF------HIAQNE

SDWSKH-----YLAVVSYKLCHMYYNWAGTVKHPHVLQMALAIVRQHSI--YIASESGFG

LESEFAPEEPPEEEPPPERPVSPELGEEPEIRERVQKRRQEVGQEAFAAGTREQRETAED

IEERNAGLENLKELKATLENKDSVLMTTAFML----------------------------

------

>Eimeria_maxima_EMWEY_00004660

----------------------------------RHDRKLEAGFLEDRLR---EMDYQQA

QLTYRNRLCVTVHKGNLYR----------IVDITD-TTADRVTFEKGGSTIT--------

--------------------------VAQYFKDAYDENVEGKQLVTAKEARGG------S

RLCYLPANLLRVIAQTPQLMEGSRAMMRPKSAQET---LVEC--T---------------

---ELVKDILRT------------------------------------------------

--PAVKEQMAELKVHI-----------------GDNGGLPIDVHKDITSLRKVMGD----

------EPRYGEPRIS--WGSERREEQQDTSVELC----------PDFRAR----LCQHL

--------VL-RHKVPLNS------HWM-LVVCT-------------------------G

RREHGSSRAVES---IKN------------------TIELTRQRIRHRGQLTVDMD----

-----------APACCEHFIR----------SEADWKKGRV-------------ERTKRS

LLDLFI---------RQRHGLRGAIVLLAYDS-D--DTNNRVRVAAK-------------

----------------------------QTKIT-------DDSLRSGIPWATDQLLAPIR

E-----------GVRPPQISEVVRVIGIAVNSFGHVFHHALPPLAEIVDKQRAPLSLNLV

ALSQPPNARCGVVGIVGGRNVAHTQFVSHIGASAPANYRA----------GVIKDMAKHF

KLFWEKLTFEESTKPHK---RAPSHLLIYRSAPS-GS----SQIKEVLAQE--VTAIKGI

LGTDLGNFEFAPE-------TGELTVEHPMATAFPDAEFALVLTVCPAAEMRRS-AGKAE

EITHHAR---------PAHETLTCQGAA---IGSDGRRTLQFKLQEGTV-PLLQAM----

------GILV--------------------SEFDVPGAEAFELRGAVLA-----------

-------DGFKGVIVPVNGEIYADVNIQET-------------------------ESSGP

------------------------------------------------------------

---IIPVATNTET-PEE-----------KFLIGTADPTRG-SPSASEF------HIAQNE

SGWSKH-----YLAVVSYKLCHMYYNWAGTVKHPHLLQMALAIVRQHSI--YIASESGFG

LESEFAPEGELEEEPAQERPASPELGEEPEIRERIQKRRQETGQEALPRTREQRETAE-D

VEERTAGLQNLRELKTALENRDSVLMTTAFML----------------------------

------

>Eimeria_acervulina_EAH_00018790

----------------------------------RHDRKLEVGFLEDRLR---EMDYQQA

LLTYKNRLCVTVHKGNLYR----------IVDIMD-TTADRATFEKGGTTIT--------

--------------------------VAKYFKDAYNENVEGKQVVTAKEARGG------S

RLCYLPVNLLRVIAQAPQPEEGSRAMLRPKSAQET---LVEC--T---------------

---ELVKDILRT------------------------------------------------

--PAVKEQMKELKVHI-----------------GDNGGLPIDVQKDITSLRKVMGD----

------EPHYGEPRIS--WGPERKEEQQDTFVELT----------PDFRAR----LCQNL

VRLHNQLPVL-RHKVPLNS------RWM-LVVCT-------------------------G

RREHGSSRAVES---IKN------------------TIELTRQRIRHRGQLTVDMD----

-----------APACCEHFIR----------SEADWKKGRV-------------ERTKRS

LRDLFI---------RQRHGLRGAVVLLAYDS-D--DTNNRVRVAAKQNRPED-------

--------------KSMNMW-WNVYLQFQTKIT-------DDSLRAGIPWAADQLLAPIR

E-----------GVRPPQISEVVRAIGIAVNSFG--------------------------

----PPNARCGVVGIVGGRNVGHTQFVSHIGASAPANYRA----------GVIKDMGKHF

KRFWEKLTFEESVKPHK---RAPAHLLIYRSAPS-GS----SQIKEVLAHE--VTAIRNV

LGTDLGSFEFSPE-------TSELIIEHPMATAFPDAEFAVVL------TLEDGEDGKRI

TL----------------PDSHPLLEYA--------KSNSRWLKNIELEFTHSPDG----

------------------------------------PLNGF-------------------

----------KGVIVPVNGEIYADVNIRAAESSPGTELSSGDTIYLRQAAISQLVQKVFI

--NVLVNPHRPANPMKFFMKG----REGVQ------------------------------

---GLPVGAYIDSGPVIPVAPNTETPEEKFLIGTADPTRG-SPSASEF------HVAQNE

SDWSKH-----FLAVVSYKLCHMYYNWAGTVKHPHLLQMALAIVRQHAI--YIASESGFG

LESEFAPEGEPEGEPAQERPVSPELGEEPEIRERVQKRRQEAGQEAL-VGTREQRETEED

AEERTAGLENLKELKAALQNRDSVLMTTAFML----------------------------

------

>Trypanosoma_cruzi_BCY84_20438

-------------------------------------QMAPSDIQELTNK--IAIWTLGQ

QESG-KKAFTVAREANGKLVCTHDSFTVGGMRIFKGTIVRAVFIDS-NGAIDTKNCAASA

LPAPPSTAVLTTLRAGHHPTEPLRFLVKEFLREFE----FKETPVQSYRIEDA------S

GVIMAS---------------LWKATKSAKLSPGT---IYEA--T---------------

---DYRVRVFEN------------------------------------------------

--RGSM-RLVEMTVGE---------TALKPVEEKQTHKKSAEVKSTSGGVEPTKRRGKKK

CVVPPTGKEEATDAINNKVTPGVTGEGSSSVMPGALALKIDTKGTVAAELSLWEEVKQHF

--------GSGPYDEATEERIARSVQGT-PVIIS-------------------------A

NLRHSIIRLVR----FNI------------------DPEKVDL--DPALQRLLPDL----

-----------EPGQPCAVLKDHSVVPLQALHCCYDPRMKSWQDISVSTCSFLPTRRLDV

LRVFHA---------VLEKEMRRWGLILASEPFA--SKELSLLPAPQKLTSYG-------

---------------VGQGASLNHKQNRSGSLR-------HPPPPSFPTTLAVVAIASPH

A-----------------EEEVRARITQTGQAVA--------------------------

---------------------------RSYRSPITMTVPDEK--------EAVRFLLQQL

TNASDGTL------------KDPNTAAVIISAER-ET----RASRWILAECLTRGILGMF

IPP--ATTVKRQN-------LLCENVRIQLRTKF-ETDPAHGVNLPREVPALA--QRRVL

VVGVDAC-HTTT------FSTGSVVGIL---CAPERNHLLPFFWKHEMRGQEADRV----

------TEHF--------------------RLVLQRACELY-------------------

-------DGLDEVVVLQDGDVSSEARQMQP--------------HLPAGC----------

--GFTFMCLHKRTNVRFVHRV----QGGNQFPKTTAA-----------------------

---NVVKGAVVQA--LTPISLQDDSVVPSFYLQNHDCNMS-TARTVQY------TIHSTS

PTLEVS-----DVQQLSHVLSHVLS--TQATKLPMATRCAHRLSSVAER--LLDAA----

------------------------------------------------------------

-------------PPLTCDMIPAPLNERLWFF----------------------------

------

>Leishmania_major_LmjF.21.0410

-----------------------------ELPPSELQMLLNKI-VPEVAR--TSHREQRQ

RVDATPAFLEVVREKAGKLVCTTQGVSAGGLRLYQ-GVLVQAIFVD-SSAALDPNAADAR

EGQVAGEAHTCSLASAKTSA------GSGTKPSTD----ADACPIATRHLGDTPVGLTDA

AAAVHF---------------QVVAFLRPFAYRGT---HAESYRIRDASGVYLASLWAPA

QPRSLTVGAVYA------------------------------------------------

--AAPV--RIREFAERGSARLI----------EFLDDTTLTLLSASTATAPASLSSG-SA

TAQPFSPRSDSGDGGN--GGA----ASEASRLPGQLSLKIDTRGTIASEVSLWEEVLQHF

--------GHGPYDEAAQQRLRKSVQGI-PVVISYSLRQSIVRDVRFDSDALLAAASHTH

NLAAAEEGRGQSVGAPHSSSPNGDAVAHDRARERLSAPVLCVR--EPRLVPLMPRL----

-----------DSQQPCAILDDHTIVPLQVLHCCFDPRMRSWQSIGVSALSLMPRQRRAL

LESIRA---------LLANGLQRWGIDLSANPYR--TKALSLLPAPTKCV----------

--------------VPQRRP--------ATGFA-------NPAAVGFPTTIVVIGVTGPR

C-----------------TAEQSRRISLTAQHLA--------------------------

---------------------------HYFRTKFVATLAD----------EGAAVQYVHE

QLMYTPAAVAAPTGQPTASLKDPNASVILITNEMDTRATRWLKVECMCRGAHFIAIPASS

NP---KKLN-----------LVGAQLRMRIATQF-ELNPLRGVDLRGELPVLG--HRHVL

VIGVDSC-HTNT------HSVGTIVGIL---STPTESKLLSYFWRHDARGREAQHV----

------AKHF--------------------RGILASAVALS-------------------

-------GRVDEVVVFQDGDVFSELVGV-K----------EELTMQVPNC----------

--GLTFMCLHKRCNVRFMHASPG--RDGSSATRSQASAAATASNDVEKDDRDTNDFREDN

NLHNLVKGVVIPA--LAPVPLHHQLAANSFYLQAHESSMS-TARIVQY------TVHHVS

PSLDVT-----DVQQIANIMANVLA--PQATKLPMSTRCAHRLAAQAER--LLD------

------------------------------------------------------------

-----AVPQLTADMIPR------PLCNRLWFL----------------------------

------

>Trypanosoma_brucei_Tb927.10.10850

---------------------------------KTVTYTYTLT-LKQATS--CSLNPKER

TMEL------------------------------N-CVIGSAVQEC--YEEK--------

--------------------------VGTKFVDLK----NGKKNAT-------------G

TISIFEAVSVK----------AFSTVIR---GKEN---DVLQ--L---------------

---DVSLPVASA------------------------------------------------

--MDCL-TAMEDERRR------------------ARGSIRRALAERFIKKKVYSVVDKSR

------ATMYTVLDIT--DNK------ACDAAGLK----------QNPSQT----YVEYF

--------RD-RYGIHIE-------PQQALFKCR-------------------------T

NDGRRV---------VLV------------------PPQVLHE----------MSL----

-----------NEQDRRQLPQ----------LCSIFPDDRV-------------QRLRRV

IERLIQ-----HEGGRVVKFLEAYGISFGKDF-V--SVDGQVLKSPEILIPKG-------

------DGFRRVN-PQSESS--------QQGFVKELKDLRHPGARQTVDVVLYDETNNKQ

G------------------TVVVGNIKKYLDGMS--------------------------

--------------------------APLNFGDSIPV-------------RSLKEAKGRL

GK------------------NIFGVTFLRRPERE-P----YAEWKASWSGG---GALSQV

VA---KDLTGGREL------SIVMAVAQQICAKTGRLNWTLDVNQVCPKLAKADPSGGIL

IIAADVGRDQRSVATESSAVRQEFFAVAFVSFYVKGTQWSTYCNHYQVN-GRKETL----

------YADGSDCDTTSMSEGGPPTPSEVISKKMGEFVQEAKSHFTSKG-----------

-------NAVSAYLVLRGCASEGELLDARK----------SDVDVLSEVLKGS-------

--SWAVVAGQRYQHSRFAFQ-----APDDR---TMYC-----------------------

---NAPRGFVTAE-GAD-----KKFGE-AFFLTGANCTLG-HARSTLY-------VVSKR

KGFDLG-----ELQALLYGMCFLYPNKTDALPLPLPLKCA---AEYGRK--YSA------

------------------------------------------------------------

----------LTNLKTL----GGNLRTTMHYL----------------------------

------

>Leishmania_braziliensis_LbrM.11.0360

---------------------------------NTEERHYRVR-IRYDGE--VSLKLPE-

-----------------------------HAQWVN-KIIAFGLADT-YSEHI--------

--------------------------GSDYVDMKSTVERGGDLVTM-------------D

AISL-----------------NALRIVKQSGSTTA---MMDVLQL---------------

---DVSTKASTK------------------------------------------------

--TKCS-DEMRRLRQQ------------------NPQGFRRAVNEALVGISVTTVFG---

-----EPTFLKVKAID--FNI----L-ASSPTMFK----------TNPEET----FVEYF

--------KR-KYDAIID-------PTL-PMLYC-------------------------I

FADRTKMSRRM----PY-------------------PADSLLL----------NKL----

-----------NEAQLSKLPI----------LCSIYPNERM-------------KRIKAA

LERVLASP-------LMITVLQQYGVRIQPQF-V--KVSGRVLPAPTIYVPSG-------

---------------------PNMFNRINTAEY-------TG--QAGFALGLKDLQHPSQ

P---------------------CEFKTLLMDEYF--------------------------

--------------------------MNGNITHWLQKYNV----------ALPSPRKTSF

DSAAQRITE-----------GPGTFAMVKLRTKEAGA---YNNFKERFARS---SIVSQM

AVVDLTR-------------NVPQMITQQVAAKIGQLCFVADVDE----AGKSFACRPLL

IVGAVVGTAMNTMLEKYKSINVRLYTITFVAFLANGKSWKPYCMHHQVK-GEEHVL---Y

EDSDAASSHMSSTTLTVRRQNANEVLNNRFPDFLKEVTAHF---KLNGK-----------

-------GSKGTMVLYRGAMTDAEVGFTAN----------MDL-VMEQVLPNW-------

--DTATVVVHPRSHFRMAWDPTTVFPHETASAYAGLS-----------------------

---NVPRGFSTTDCRIILADSDPYTPVDSFYLSAANCTLG-HAANTYY------LVQKRA

ASISLM-----DLQKLTYNMCYMYPNKPDALPLPLPIKCAYEYARKYGS--LKS------

------------------------------------------------------------

----------VKELPTR-------MRPTMHYL----------------------------

------

>Giardia_Assemblage_GL50803_2902

---------------------------------MRNNIAGPPI-TPVQAT--VKVTRVRQ

MDPT--DLMS-------------------MKILLN-ILLRRTFESS-LGMLN--------

-------------------I-------RSGFYDLR-----NPSIHAIQIDGRG------Y

DICWIP---------------GFRLTTAT--LRGK---LGLQ--I---------------

---LPETTKVRS------------------------------------------------

--KSMN--------------------------ELLTERRGNIHPSALAVITVMAMHN---

------GKVFRVHSIV--QGQ----T---IVSPLA----------NGNETD----YFTYY

--------ST-RYRDKIDS------AGL-SLLRHNDYCNSVMQQDKFILKLTPLKRTQNG

KVVRP----------CNV------------------PSSLCIIISDNEIAPYGVSK----

-----------LSTNRAAVA-----------LSTMSPDALL-------------EKATAF

AARLAEDT-------ELQSLLGDYGFGFTSNP-L--ELETFVCKPPKLMMDNT-------

--------SRIVTIEDDSGGVFHNLLQ-SPGVSPIYYSNNNQPATGMPVWAIMVPRNLGN

D----------------YARRLRKELTERVRSLA--------------------------

-----------------------GTTAPNIGDPPLIAVELSNQRREM---YRVEPYKDAF

KALLNKLRLEYKDARESEIVAKIQLVVIVIPGPKQDRGDLYKGIKQFYTH---MGIVTQC

LLA-PKSSQNELQWYDQ---AVLRSVCQQIYAKAGGAVWAPVL------PPQNVYSKSTM

LCALDIS-RPKKTVGRPAEVPISTAGFI---STYDGSFEYIYSQKKTLV-PNRLNH----

------GGEL--------------QQQTLMEAFIKNSCNVY--LAFNNR-----------

-------ILPDHIVIFRDGVSDSQISATLE----------VEIKSLYECLRQIYHKSNRP

MCDLKVIVAQKTCAMRFS-------AVGGT------------------------------

---VLRSGYYVINRSPD-----NRQQGSEFLMASQAIVHGTTPKPIRY------KIIFDS

MEASVDNDSFNQLIELTNAMAYGYVNWPQAISLPHVLHMAHHLSKFCGE--VLRNG----

------------------------------------------------------------

-------------DDLFESCAIFGLQYRPFFI----------------------------

------

>Toxoplasma_gondii_ME49_TGME49_310160

---------------------------------CMKKLEYIE--MLRGVS--LSIHPQQS

VIMA--NLGEQLSMQNSATEE--------VAQILQ-LIMNHAAVME-GYQMF--------

-------------------H------GVNFFKDMP----ENFVPL----RGPN------K

AFQIWD---------------GFSQAVAPYQTAGT---MSWNAVF---------------

---NLRACTSTK------------------------------------------------

--AIPLVKYIEIQASAIAKRMVDLSTEKGCRALMADAEVMRRLNRRLRGTKLESFHILNR

DTQQPEKRVYKLKELMTFSAS----S-EESRFDLQ----------DGRRVT----VLQHF

--------KE-TYPHASGI------PPFQPLINT-------------------------R

SKDRP----------AYL------------------PVSIVTL----KHQPAREGV----

-----------TEEDRAQVAE----------HMIMPPRNRV-------------AKTEQL

LSLVFGP-----QGHSAPKVLDAFGVILQLEA-K--TAQGRVLESPLIKYREV-------

-PGGGPGRSSKTV-RPAQGD-WNL--R-DAAFC-------RG--TVCKVWALYSFVETSQ

N----------------VIENLARVLKTQGAKYG--------------------------

--------------------------VNLTTKPGLGAYTR----------NDRRPMLDQF

AAFVALAKT-----------KGCELLFVILNERV-SL-DIYQIVKSCTD----VNFPSQC

LNGRHKCIDAIFRGADNPNPQYFANVMSKVNMKLQGVNQTLEADIIKQ-EIGT--DKSTL

VLAVETSFFANPTKTSPPPTAPIVCACT---GNMD-DDLGAFGHAVCVE-SRKHPI----

------VTDI---------------------GSMFKTILSY--RKTT-K-----------

-------NWPARIIYLRSATTEAHFPLVLA----------GEIRAIEELYVR--ENRSKP

--RILAVAVQRRQQTRLFPT-----KEMQA----QGN-----------------------

---NLPPGFLLAN-SLQ-----HPGHFRNFLLISHKALQG-TARPTRY------YILRDD

ANRDME-----KVAQLMYSLCHVYGRCQRAVSIPAPLYYAELLAARAQS--YMKVGMRRE

R-----NIDIDDLSHL-------------------------------------------S

GEAGEKMLTETRHFADEYLRSTAAKVTPMVFC----------------------------

------

>Toxoplasma_gondii_VEG_TGVEG_310160

---------------------------------CMKKLEYIE--MLRGVS--LSIHPQQS

VIMA--NLGEQLSMQNSATEE--------VAQILQ-LIMNHAAVME-GYQMF--------

-------------------H------GVNFFKDMP----ENFVPL----RGPN------K

AFQIWD---------------GFSQAVAPYQTAGT---MSWNAVF---------------

---NLRACTSTK------------------------------------------------

--AIPLVKYIEIQASAIAKRMVDLSTEKGCRALMADAEVMRRLNRRLRGTKLESFHILNR

DTQQPEKRVYKLKELMTFSAS----S-EESRFDLQ----------DGRRVT----VLQHF

--------KE-TYPHASGI------PPFQPLINT-------------------------R

SKDRP----------AYL------------------PVSIVTL----KHQPAREGV----

-----------TEEDRAQVAE----------HMIMPPRNRV-------------AKTEQL

LSLVFGP-----QGHSAPKVLDAFGVILQLEA-K--TAQGRVLESPLIKYREV-------

-PGGGPGRSSKTV-RPAQGD-WNL--R-DAAFC-------RG--TVCKVWALYSFVETSQ

N----------------VIENLARVLKTQGAKYG--------------------------

--------------------------VNLTTKPGLGAYTR----------NDRRPMLDQF

AAFVALAKT-----------KGCELLFVILNERV-SL-DIYQIVKSCTD----VNFPSQC

LNGRHKCIDAIFRGADNPNPQYFANVMSKVNMKLQGVNQTLEADIIKQ-EIGT--DKSTL

VLAVETSFFANPTKTSPPPTAPIVCACT---GNMD-DDLGAFGHAVCVE-SRKHPI----

------VTDI---------------------GSMFKTILSY--RKTT-K-----------

-------NWPARIIYLRSATTEAHFPLVLA----------GEIRAIEELYVR--ENRSKP

--RILAVAVQRRQQTRLFPT-----KEMQA----QGN-----------------------

---NLPPGFLLAN-SLQ-----HPGHFRNFLLISHKALQG-TARPTRY------YILRDD

ANRDME-----KVAQLMYSLCHVYGRCQRAVSIPAPLYYAELLAARAQS--YMKVGMRRE

R-----NIDIDDLSHL-------------------------------------------S

GEAGEKMLTETRHFADEYLRSTAAKVTPMVFC----------------------------

------

>Toxoplasma_gondii_GT1_TGGT1_310160

---------------------------------CMKKLEYIE--MLRGVS--LSIHPQQS

VTMA--NLGEQLSMQNSATEE--------VAQILQ-LIMNHAAVME-GYQMF--------

-------------------H------GVNFFKDMP----ENFVPL----RGPN------K

AFQIWD---------------GFSQAVAPYQTAGT---MSWNAVF---------------

---NLRACTSTK------------------------------------------------

--AIPLVKYIEIQASAIAKRMVDLSTEKGCRALMADAEVMRRLNRRLRGTKLESFHILNR

DTQQPEKRVYKLKELMTFSAS----S-EESRFDLQ----------DGRRVT----VLQHF

--------KE-TYPHASGI------PPFQPLINT-------------------------R

SKDRP----------AYL------------------PVSIVTL----KHQPAREGV----

-----------TEEDRAQVAE----------HMIMPPRNRV-------------AKTEQL

LSLVFGP-----QGHSAPKVLDAFGVILQLEA-K--TAQGRVLESPLIKYREV-------

-PGGGPGRSSKTV-RPAQGD-WNL--R-DAAFC-------RG--TVCKVWALYSFVETSQ

N----------------VIENLARVLKTQGAKYG--------------------------

--------------------------VNLTTKPGLGAYTR----------NDRRPMLDQF

AAFVALAKT-----------KGCELLFVILNERV-SL-DIYQIVKSCTD----VNFPSQC

LNGRHKCIDAIFRGADNPNPQYFANVMSKVNMKLQGVNQTLEADIIKQ-EIGT--DKSTL

VLAVETSFFANPTKTSPPPTAPIVCACT---GNMD-DDLGAFGHAVCVE-SRKHPI----

------VTDI---------------------GSMFKTILSY--RKTT-K-----------

-------NWPARIIYLRSATTEAHFPLVLA----------GEIRAIEELYVR--ENRSKP

--RILAVAVQRRQQTRLFPT-----KEMQA----QGN-----------------------

---NLPPGFLLAN-SLQ-----HPGHFRNFLLISHKALQG-TARPTRY------YILRDD

ANRDME-----KVAQLMYSLCHVYGRCQRAVSIPAPLYYAELLAARAQS--YMKVGMRRE

R-----NIDIDDLSHL-------------------------------------------S

GEAGEKMLTETRHFADEYLRSTAAKVTPMVFC----------------------------

------

>Neospora_caninum_NCLIV_054260

---------------------------------------------------------FMG

KLEY--------------------------------LIMNHAAVQE-GYQMF--------

-------------------N------GVNFFKDLP----ENFFPL----RGQN------K

AFQIWD---------------GFSQAVAPYQTSGA---LSWNAVF---------------

---NLRACTATK------------------------------------------------

--AIPLVKYIEIQASAIAKRMVDLSTDKGCRALMADAEVMRRLNKRLRGTKVESFHILNR

DTKRPEKRVYKLKELMTFSAS----S-EESRFDLQ----------DGRTVT----VLQHF

--------QE-SYPHVSGI------PPFQLLINT-------------------------R

SKNRP----------AYL------------------PISIVTL----KHQPVREGI----

-----------TDEDRAQIAE----------HMIMPPRNRV-------------AKSEQL

LSLVFGP-----QGGSATKVLDAFGVVLQREA-K--TAEGKVLESPLIKYREV-------

-PGGIPGRSTKTI-RPAQGD-WNL--R-DAAFC-------RG--TVCRVWALYSFVETNQ

N----------------VIEKLARVLKAQGATYG--------------------------

--------------------------VNLTTKPGLGAYTR----------SDRRAMLDQF

AAF-VAL--AKT--------KGCELLFVILNERV-SM-EIYQIVKSC--TD--VNFPSQC

LNGRHKCIEAIFRG-DNPNPQYFANVMSKVNMKLQGVNQTLEADILKQ-EIGM--DKSTL

VLSVETSFFSNPSKPRPPPTAPVVCACT---GNVD-DDLGVFGHAVCVE-SRKEPI----

------VTDI---------------------GKMFRMILSY--RKTA-K-----------

-------NWPARIIYLRSATTEAHFPLVLA----------GEIRAIEELYVR--ENRPKP

--RILAVAVQKRQQTRLFPT-----KEMQS----QGN-----------------------

---NLPPGFLLAD-SLQ-----HPGQFRNFLLISHKALQG-TARPTRY------YILRDD

ANRDIE-----RVAQLMYSLCHAYGRCQRAVSIPAPIYYAELLAARAQS--YMKVGMRRE

R-----NIDMDDLSHL-------------------------------------------S

GESGEKILAETRQFADEYLRSTAARVTPMVFC----------------------------

------

>AGO2_NP_730054_Drosophila_melanogaster

------------------------------------------IEIKETGDSTIDLKSLTT

YMND--RIFDKPMR---------------AMQCVE-VVLASPCHNK--AIRV--------

--------------------------GRSFFKMSD---PNNRHELD-------------D

GYEALV---------------GLYQAFM---LGDR---PFLN--V---------------

---DISHKSFPI------------------------------------------------

--SMPMIEYLERFSLK-----------AKINNTTNLDYSRRFLEPFLRGINVVYTPPQSF

QSA---PRVYRVNGLS--RAP----A-SSETFEHD-----------GKKVT----IASYF

--------H--SRNYPLKF------PQL-HCLNV-------------------------G

SSIKS----------ILL------------------PIELCSI---EEGQALNRKD----

-----------GATQVANMIK----------YAATSTNVRK-------------RKIMNL

LQYFQH---------NLDPTISRFGIRIANDF-I--VVSTRVLSPPQVEYHSK-------

----------RFT-MVKNGS-WRM--D-GMKFL-------EP--KPKAHKCAVLYCDPRS

G--------RKM--NYTQLNDFGNLIISQGKAVN--------------------------

--------------------------ISLDSDVTYRPFTDDE--------RSLDTIFADL

K----------R--------SQHDLAIVIIPQFR-IS---YDTIKQKAELQ--HGILTQC

IKQ--FTVERKCNN------QTIGNILLKINSKLNGINHKIKDDPR--LPM----MKNTM

YIGADVT-HPSP----DQREIPSVVGVA---ASHD-PYGASYNMQYRLQ-RGALEE----

------IEDM--------------------FSITLEHLRVY--KEYRNA-----------

--------YPDHIIYYRDGVSDGQFPKIKN----------EELRCIKQACDKV---GCKP

--KICCVIVVKRHHTRFF-------PSGDVTTSNKFN-----------------------

---NVDPGTVVDR-TIV-----HPNEM-QFFMVSHQAIQG-TAKPTRY------NVIENT

GNLDID-----LLQQLTYNLCHMFPRCNRSVSYPAPAYLAHLVAARGRV--YLTGT----

------------------------------------------------------------

----NRFLDLKKEYAKRTIVPEFMKKNPMYFV----------------------------

------

>AGO1_XP_001694840_Chlamydomonas_reinhardtii

--------------------------------RPPPDEEQPEGSPAPAGV--LPPPPAPP

ATSG--AA---------------------ALAVLD-LVTRAGVAWD-VASVR--------

-------------------T------GGGSYV-------SYKQEDASGPVKGL------L

PMKLLR---------------GFRSAVAQ--VAAG---TTLT--I---------------

---DAASAVVAG------------------------------------------------

--DGPLLGQLQAALAA-------GRDVSALATELGNSKNARRLEEALVGLEVQPSYT---

------ANRYKLSGKL--GKS----A-RSLAFEFE-----------GRKIS----VQTYF

--------KE-RYGVSLQH------PDL-PCVLD--------------------------

--KKG----------GAL------------------PIELLTV---VKYQKRMSRL----

-----------DARQKADYIR----------TAALKPADRM-------------RAMREA

LTRQLGS--------VRGQVARAFGLAPFNDMPDFLKVPAQLLPGPLLQVAKQ-------

----------QAVEPGNKGE-WA-----GGGVA-------VA--PEWRSGAVLCYLSQSE

G--------------QAPLSGLLAALSKALSASS--------------------------

--------------------------ASALPPNIPVIWASLPKP------GMSPDKAAAD

AEFWLNRAAAKAQEQYK---RQAQMVMVVLPDRG-GV-GLYDAIKAAGDGK--LGVATQC

VNP-AKAGLVGPKASSGPSFSYAAQLALSVNAKLGGATTR---------PAGR--PNGEL

ALALPRTRRTRV----QVSAGVEYAAVV---GSAD-SHAVDYRVQLSAQVAGNRDI----

------VVSM--------------------RESVRRLLLQY--GLPQGG-------AVAA

GGPRPSSELPEVVLMYRDGLSESQFDRALA----------EEFTAIVQACADVGGPSYRP

--RICFVVVQKSHNTRFFPT-----SAEST---HKSG-----------------------

---NVLPGTAVDR-GVT-----DPHAF-DFFLNSHAGIQG-TNRAPRY------TVLVDE

VGFTAE-----ALQLLTHTLCHTYPACTRALSLPPPVRYADRAADRARTLRYMVPR----

------------------------------------------------------------

-------PSGKQGRGVDWYQMLPTLPHAGHVV----------------------------

------

>Schizosaccharomyces_pombe_NP_587782_

----------------------------------------IEFSIQKSSK--INLHTLSQ

FVNS--KYSSDPQVLS-------------SIMFLD-LLLKKKPSET-LFGFM--------

---------------------------HSFFTGEN----GVSLG---------------G

GVEAWK---------------GFYQSIRP--NQGF---MSVN--V---------------

---DISSSAFWR------------------------------------------------

--NDSLLQILMEYTDC-------------SNVRDLTRFDLKRLSRKFRFLKVTCQHR-NN

VGTDLANRVYSIEGFS--SKS----A-SDSFFVRR-------LNGEEQKIS----VAEYF

--------LE-NHNVRLQY------PNL-PCILV--------------------------

--KNG----------AML------------------PIEFCFV---VKGQRYTAKL----

-----------NSDQTANMIR----------FAVQRPFERV-------------QQIDDF

VHQMDW---------DTDPYLTQYGMKIQKKM-L--EVPARVLETPSIRYGGD-------

----------CIE-RPVSGR-WNL--R-GKRFL-------DPPRAPIRSWAVMCFTSTRR

L-------------PMRGIENFLQTYVQTLTSLG--------------------------

--------------------------INFVMKKPPVLYADIR--------GSVEELCITL

YKKAEQVGN-----------APPDYLFFILDKNS-PE--PYGSIKRVCNTM--LGVPSQC

AIS--KHILQSKP-------QYCANLGMKINVKVGGINCSLIPKSN---PLG---NVPTL

ILGGDVY-HPGV-----GATGVSIASIV---ASVD-LNGCKYTAVSRSQ-PRHQEV----

------IEGM--------------------KDIVVYLLQGF--RAMT-K-----------

-------QQPQRIIYFRDGTSEGQFLSVIN----------DELSQIKEACHSL-SPKYNP

--KILVCTTQKRHHARFFIK-----NKSDG---DRNG-----------------------

---NPLPGTIIEK-HVT-----HPYQY-DFYLISHPSLQG-VSVPVHY------TVLHDE

IQMPPD-----QFQTLCYNLCYVYARATSAVSLVPPVYYAHLVSNLARY--QDVTA----

-----------------------------------------------------------D

DTFVETSEASMDQEVKPLLALSSKLKTKMWYM----------------------------

------

>AGO3_NP_174414_Arabidopsis_thaliana

---------------------------------------YTFI-IKQVKE--LKLLDLQA

YIDG--RSTFIPRD---------------VLQGMD-VVMKEHPSKR--MITV--------

--------------------------GKRFFSTRL----EIDFG---------------Y

GVGAAK---------------GFHHTLKP--TVQG---LSLC--L---------------

---NSSLLAFRK------------------------------------------------

--AISVIEYLKLYFGW-------------RNIRQFKNCRPDDVVQELIGLKVTVDHRKT-

------KQKFIIMGLS--KDD----T-KDIKFDFI----DHAGNQPPRKIS----IVEYF

--------KE-KYGRDIDH------KDI-PCLNL-------------------------G

KKGRE----------NFV------------------PMEFCNL---VEGQIFPKEK----

-----------LYRDSAAWLKE---------LSLVTPQQRL-------------ENINKM

IKSSDGP--------RGGDIIGNFGLRVDPNM-T--TVEGRVLEAPTLKLTDR-------

-----RGNPIHEKLMSESNQ-WNLT---TKGVT-------KG--SIIKHWAVLDFTASES

L-------------KKKMPGYFVNKLIERCKGLG--------------------------

--------------------------MQMEA-PIVCKTSSMETLYDG---NALEELLRSV

IDEASHNHGG----------ACPTLVLCAMTGKH-DG---YKTLKWIAETK--LGLVTQC

FLT--ISAIKGETVSD----QYLANLALKINAKVGGTNVELVDNI----FSFFKKEDKVM

FIGADVN-HPAA----HDNMSPSIVAVV---GTLNWPEANRYAARVKAQ-SHRKEE----

------IQGF--------------------GETCWELIEAH--SQAPEK-----------

--------RPNKIVIFRDGVSDGQFDMVLN----------VELQNVKDVFAKV---GYNP

--QITVIVAQKRHQTRFFPA-----TTSKD--GRAKG-----------------------

---NVPSGTVVDT-TII-----HPFEY-DFYLCSQHGAIG-TSKPTHY------YVLSDE

IGFNSN-----QIQKLIFDLCFTFTRCTKPVALVPPVSYADKAASRGRV--YYEASLMKK

N-----SKQSRGASSS-------------------------------------------S

ASVASSSSSVTMEDKEIFKVHAGIE-NFMFFV----------------------------

------

>AGO2_NP_174413_Arabidopsis_thaliana

---------------------------------------YTFT-IKQVNV--LKLGDLKE

YMTG--RSSFNPRD---------------VLQGMD-VVMKEHPSKC--MITV--------

--------------------------GKSFFTRET----EPDEDFR-------------F

GVIAAK---------------GYRHTLKP--TAQG---LSLC--L---------------

---DYSVLAFRK------------------------------------------------

--AMSVIEYLKLYFNW----------------SDMRQFRRRDVEEELIGLKVTVNHRKN-

------KQKLTIVGLS--MQN----T-KDIKFDLI----DQEGNEPPRKTS----IVEYF

--------RI-KYGRHIVH------KDI-PCLDL-------------------------G

KNGRQ----------NFV------------------PMEFCDL---VEGQIYPKDN----

-----------LDKDSALWLK---------KLSLVNPQQRQ-------------RNIDKM

IKARNGP--------SGGEIIGNFGLKVDTNM-T--PVEGRVLKAPSLKLAER-------

------GRVVREEPNPRQNNQWNLM---KKGVT-------RG--SIVKHWAVLDFTASER

F--------------NKMPNDFVDNLIDRCWRLG--------------------------

--------------------------MQMEAPIVYKTSRMETLSNG----NAIEELLRSV

IDEASRKHGG----------ARPTLVLCAMSRKD-DG---YKTLKWIAETK--LGLVTQC

FLT--GPATKGGD-------QYRANLALKMNAKVGGSNVELMDT-----FSFFKKEDEVM

FIGADVN-HPAA----RDKMSPSIVAVV---GTLNWPEANRYAARVIAQ-PHRKEE----

------IQGF--------------------GDACLELVKAH--VQAT-G-----------

-------KRPNKIVIFRDGVSDAQFDMVLN----------VELLDVKLTFEKN---GYNP

--KITVIVAQKRHQTRFFPA-----TNNDG---SDKG-----------------------

---NVPSGTVVDT-KVI-----HPYEY-DFYLCSHHGGIG-TSKPTHY------YTLWDE

LGFTSD-----QVQKLIFEMCFTFTRCTKPVSLVPPVYYADMVAFRGRM--YHEASSREK

------NFKQPRGAST-------------------------------------------S

AASLASSLSSLTIEDKAIFKLHAELENVMFFV----------------------------

------

>AGO6_NP_180853_Arabidopsis_thaliana

---------------------------------------YKVQ-IHYAAE--IPLKTVLG

TQRG--AYTPDKSAQD-------------ALRVLD-IVLRQQAAER-GCLLV--------

--------------------------RQAFFHSDG-------HPMK---VGG--------

GVIGIR---------------GLHSSFRP--THGG---LSLN--I---------------

---DVSTTMILE------------------------------------------------

--PGPVIEFLKANQSV----------------ETPRQIDWIKAAKMLKHMRVKATHR---

------NMEFKIIGLS--SKP----C-NQQLFSMK--IKDGEREVPIREIT----VYDYF

--------KQ-TYTEPISS------AYF-PCLDV-------------------------G

KPDRP----------NYL------------------PLEFCNL---VSLQRYTKPL----

-----------SGRQRVLLVE----------SSRQKPLERI-------------KTLNDA

MHTYCY---------DKDPFLAGCGISIEKEM-T--QVEGRVLKPPMLKFGKN-------

-----------EDFQPCNGR-WNF--N-NKMLL-------EP--RAIKSWAIVNFSFPCD

S------------------SHISRELISCGMRKG--------------------------

--------------------------IEIDRPFALVEEDPQ---------YKKAGPVERV

EKMIATMKLKFP--------DPPHFILCILPERK-TS-DIYGPWKKICLTE--EGIHTQC

ICP--IKISD----------QYLTNVLLKINSKLGGINSLLGIEYSYNIPLIN--KIPTL

ILGMDVS-HGPP----GRADVPSVAAVV---GSKCWPLISRYRAAVRTQ-SPRLEM----

------IDSL---------FQPIENTEKGDNGIMNELFVEF--YRTSRA-----------

-------RKPKQIIIFRDGVSESQFEQVLK----------IEVDQIIKAYQRL-GESDVP

--KFTVIVAQKNHHTKLFQA------KGPE------------------------------

---NVPAGTVVDT-KIV-----HPTNY-DFYMCAHAGKIG-TSRPAHY------HVLLDE

IGFSPD-----DLQNLIHSLSYVNQRSTTATSIVAPVRYAHLAAAQVAQ--FTKFE----

------------------------------------------------------------

-------GISEDGKVPELPRLHENVEGNMFFC----------------------------

------

>AGO9_NP_197613_Arabidopsis_thaliana

---------------------------------------FMVE-ISYAAK--IPMQAIAS

ALQG--KETENLQD---------------ALRVLD-IILRQSAARQ-GCLLV--------

--------------------------RQSFFHNDV----KNFVPIG-------------G

GVSGCR---------------GFHSSFRT--TQGG---LSLN--I---------------

---DTSTTMIVQ------------------------------------------------

--PGPVVDFLLANQNK----------------KDPYGMDWNKARRVLKNLRVQITLS---

------NREYKISGLS--EHS----C-KDQLFTWR--KPNDKGEFEEVEIT----VLNYY

--------K--ERNIEVRY------SGDFPCINV-------------------------G

KPKRP----------TYF------------------PIEFCNL---VSLQRYTKSL----

-----------TNFQRAALVE----------KSRQKPPERM-------------ASLTKG

LKDSNY---------NADPVLQDSGVSIITNF-T--QVEGRILPTPMLKVGKG-------

-----------ENLSPIKGK-WNF--M-RKTLA-------EP--TTVTRWAVVNFSARCD

T------------------NTLIRDLIKCGREKG--------------------------

--------------------------INVEPPFKDVINENPQ--------FRNAPATVRV

ENMFEQIKSKLP--------KPPLFLLCILAERK-NS-DVYGPWKKKNLVD--LGIVTQC

IAP--TRLND----------QYLTNVLLKINAKLGGLNSLLAMERSPAMPKVT--QVPTI

IVGMDVS-HGSP----GQSDIPSIAAVV---SSRQWPLISKYKACVRTQ-SRKMEM----

------IDNL------------FKPVNGKDEGMFRELLLDF--YYSSEN-----------

-------RKPEHIIIFRDGVSESQFNQVLN----------IELDQMMQACKFL-DDTWHP

--KFTVIVAQKNHHTKFFQS------RGPD------------------------------

---NVPPGTIIDS-QIC-----HPRNF-DFYLCAHAGMIG-TTRPTHY------HVLYDE

IGFATD-----DLQELVHSLSYVYQRSTTAISVVAPVCYAHLAAAQMGT--VMKYEELSE

-----------------------------------------------------------T

SSSHGGITTPGAVPVPPMPQLHNNVSTSMFFC----------------------------

------

>AGO4_NP_565633_Arabidopsis_thaliana

---------------------------------------FRVE-ISYAAK--IPLQALAN

AMRG--QESENSQE---------------AIRVLD-IILRQHAARQ-GCLLV--------

--------------------------RQSFFHNDP----TNCEPVG-------------G

NILGCR---------------GFHSSFRT--TQGG---MSLN--M---------------

---DVTTTMIIK------------------------------------------------

--PGPVVDFLIANQNA----------------RDPYSIDWSKAKRTLKNLRVKVSPS---

------GQEFKITGLS--DKP----C-REQTFELKKRNPNENGEFETTEVT----VADYF

--------RD-TRHIDLQY------SADLPCINV-------------------------G

KPKRP----------TYI------------------PLELCAL---VPLQRYTKAL----

-----------TTFQRSALVE----------KSRQKPQERM-------------TVLSKA

LKVSNY---------DAEPLLRSCGISISSNF-T--QVEGRVLPAPKLKMGCG-------

-----------SETFPRNGR-WNF--N-NKEFV-------EP--TKIQRWVVVNFSARCN

V--------R----------QVVDDLIKIGGSKG--------------------------

--------------------------IEIASPFQVFEEGNQF--------RRAPPMIRVE

NMF-KDIQSKLP--------GVPQFILCVLPDKK-NS-DLYGPWKKKNLTE--FGIVTQC

MAP-----TRQPND------QYLTNLLLKINAKLGGLNSMLSVERTPAFTVIS--KVPTI

ILGMDVS-HGSP----GQSDVPSIAAVV---SSREWPLISKYRASVRTQ-PSKAEM----

------IESL------------VKKNGTEDDGIIKELLVDF--YTSSNK-----------

-------RKPEHIIIFRDGVSESQFNQVLN----------IELDQIIEACKLL-DANWNP

--KFLLLVAQKNHHTKFFQP-----TSPE-------------------------------

---NVPPGTIIDN-KIC-----HPKNN-DFYLCAHAGMIG-TTRPTHY------HVLYDE

IGFSAD-----ELQELVHSLSYVYQRSTSAISVVAPICYAHLAAAQLGT--FMK------

------FEDQSETSSS-------------------------------------------H

GGITAPGPISVAQLPRL---KDNVA-NSMFFC----------------------------

------

>AGO_XP_013896353_Monoraphidium_neglectum

------------------------------------------------------------

------------------------------------------------------------

------------------------------------------------------------

------------------------------------------------------------

------------------------------------------------------------

------------------------------------------------------------

------------------MGA----D-KTMFYYEP----------EKREIS----VAQYF

--------QV-AYKIALKL------PRL-PCVNT-------------------------S

KDVTK----------LVW-----------------LPIEFCHI---LPGQRKRMLQ----

-----------DERHTTAMLR----------FAGLKPHERA---RYLKGARVKEDDVVSN

PRLANF---------NAEPAVQRFGMQIAQDM-V--NVQGRILDPPKLAYGKP-------

---------EALD-PGTQGA-WNL--R-QVEFP-------VA--APLSSWACVSLMDQSE

V--------DLQEGHPQSLKTFVAELVSMCNSCG--------------------------

--------------------------MKTVVPPIIHF-------------DHSYSVGEHI

SYAVEEAQNTFK--------AKCKLVLVLLPSKG-KD--TYQAVKQASDSE--CSVPTQC

FVAQKAGVGRSSPPAKGRL-QYCANLALKMNVKVGGTNVKLLGDLKQMPVLGS--NQPFM

I-SADVT-HPTG----FDSSEPSIAAVT---ASYE-------------------------

----------------------------------WAMLLNF--YRRTNS-----------

-------NKPAAIIYYRDGVDEGQFAAVLK----------EEYTAIRSACQGL-EAGYTP

--RITFVVVQKRHATRLFPK-----DRSGE---DRSG-----------------------

---NTLPGTVLDA-GIC-----SAAGF-DFFLNSHAGLQG-HNKASHYQARRPSHVLVDE

NGFSAD-----GLQLLTYWLTYLYARCTRSVSYPPP-YLAHLAAFRGRL--MLDVPD---

-----------------------------------------------------------S

GSESSRGSRGGRPAPLL-SIKDDFSDTTMFYV----------------------------

------

>AGO3_XP_001698906_Chlamydomonas_reinhardtii

RFDGRKNLFLPGELLPREVREWPVTLKPREGDKSERDKGFVVT-TKWAAC--VGLTQLQD

YLAR--RQQTAPRD---------------AMQVLD-IVIRHAFAID-PRCTV--------

-------------------V------GRGFYYGGE-----GVMP-----LGG--------

GAEVWS---------------GFQQSFKA--VQAG---LTLN--L---------------

---DSSFAAFMS------------------------------------------------

--ARPLPELLAE------------------------------------GAGVEFPMPGGR

------ARRKALVGLS--EQG----ADRTMFMNEK----------EGREMS----VAEYF

--------R--STGRPLRH------PGL-PCANV-------------------------G

DRRRA----------VFI------------------PVELCTV---VAGQRRMKL-----

-----------DATQSAGMIT----------AAKQDPAVKK-------------EACDKQ

AKRVAEAL-------AAGGTDRCWGLKLATGM-L--PVQGRMLPNPVLH-----------

----------------------------NVKFV-------DP--RALDSWGVAVMMNQAD

V------DFDGDNSLWQFLEDLTGGMIRCGMRVA--------------------------

--------------------------SPVTAASTDSPPVEFGGMPGGGGRGGGRGIEATM

RAAADAAAARYK--------KPAQLVLVVLPEKT-AD--EYREVKRVSDIE--LGIPSQV

VVASKARVGYRAHKGGGP--QYCANVAMKINNKLGGVNVQLSGGLRNMPVLGGAGAVPFM

VLGADVT-HPTGAAARADSRDPSVAAVV---ASLD-ASLGRWASRVLLQ-AGRQEV----

------ITGM--------------------CGATKELLLEF--YRANKQ-----------

-------VKPQRLVMYRDGVSEGQFEQVLA----------EEYTALRRACREL-EEGYRP

--AITFVVVQKRHNTRLLPS-----DRAAS---DQKG-----------------------

---NVVPGTVVDS-GIT-----APDGF-DFYLNSHAGLQG-TNKPAHY------HVLVDE

IGFGAD-----GIQLLTYWLCYLYQRTTKSVSYCPPAYYADRAAFRGRT--LLAASSS--

------ASDSASETAS-------------------------------------------R

SGRGAGAAEGGASAPPTFAGIHRNLTNVLYFM----------------------------

------

>AGO2_XP_001698670_Chlamydomonas_reinhardtii

--GVGGSGNASDRVELASSVTRPSPIPFRNPGRALRSAELQAAGEDSAGRAGIGAAGTAA

AGPGRNGACSVAAGGGQGGANGGNSLSYECSNGND-RITTGATGSETSLAHSTFQHSPAA

QHPPAAQHPPAVQLRAGPQLASAKAADVQRYLDQAAAAHAAAESAARHAHAHLSAAATVA

AAALAGHTSRHGGFRAAAGEVGEATAADAATGAGAPAAGTLA--AGGAAGECDFSRREPG

VEGSISHRVKRRRGGDAANKPAGLGYGSGASASDGDEEVGADDDPDYGPDGHHCGGDDDA

GAAAAVPSWVIPTCVSTCYPALPPQVLWPAPPHGQEEQQAQQAQQPLWDAQVPMRPPPHA

------PRQGLHWSAAAGNGRGEFLG-SAPAVPMQ-----------GLQMQQPWHLQHEV

--------EA-MAEATAEAGEVETTPDGRPLGIT-------------------------R

RPNAGTVGKAVALLANYFALATTPAFPGQAYHY---DVEIRSVEEAAGGEGLLPREVREW

PVTLKPREGDKSERDKGFVVATKWAACVGLTQLQDYLAQRQ-------------QTAPRD

AMQSFKAAKRVAEALAAGGTERSWGLKLGTGM-L--PVQGRVLPNPVLQYGNR-------

---------QDFD-AGPLGS-WNT--L-NVKFV-------DA--RALDSWAVA-------

------------------------VMMNQAARVG--------------------------

------------------------------------------------------------

----------------------------------------YREQR--------GGGP---

--------------------QYCANVAMKINNKLGGVNVQLSGGLRYMPVLGGAGSVPFM

VLGADVK-----------------------------------------------------

------------------------------------------------------------

---------PQRLVMYRDGVSEGQFEQVLA----------EEFTALRRACREL-EEGYRP

--AITFVVVQKRHNTRLLPN-----DRASA---DPKG-----------------------

---NVVPGTVVDS-GIT-----APDGF-DFYLNSHSGLQV-------------------G

GGWGGG-------------------GCRFQPKLANPMQ----------------------

------------------------------------------------------------

------------------------------------------------------------

------

>AGO7_NP_177103_Arabidopsis_thaliana

-----------------------------------LREKQPQKKIEKLFR--VNMKLVSK

FDGK--EQRKEGEDWAPLPPE--------YIHALD-VILRENPMEK--CTSI--------

--------------------------GRSFYSSSM----GGSKE-----IGG--------

GAVGLR---------------GFFQSLRH--TQQG---LALN--M---------------

---DLSITAFHE------------------------------------------------

--SIGVIAYLQKRLEF-------LTDLPRNKGRELSLEEKREVEKALKNIRVFVCHRET-

------VQRYRVYGLT--EEI----T-ENIWFPDR----------EGKYLR----LMSYF

--------KD-HYGYEIQF------KNL-PCLQI--------------------------

SRARP----------CYL------------------PMELCMI---CEGQKFLGKL----

-----------SDDQAAKIMK----------MGCQKPNERK-------------AIIDKV

MTGSVGP--------SSGNQTREFNLEVSREM-T--LLKGRILQPPKLKLD---------

--------------RPRNLK--------ESKVF-------KG--TRIERWALMSIGGSSD

Q--------KST------IPKFINELTQKCEHLG--------------------------

--------------------------VFLSKNTLSSTFFEP---------SHILNNISLL

ESKLKEIQRAAS--------NNLQLIICVMEKKH-KG---YGDLKRISETR--IGVVTQC

CLY--PNITKLSS-------QFVSNLALKINAKIGGSMTELYNSIPSHIPRLLRPDEPVI

FMGADVT-HPHP----FDDCSPSVAAVV---GSINWPEANRYVSRMRSQ-THRQEI----

------IQDL--------------------DLMVKELLDDF--YKAV-K-----------

-------KLPNRIIFFRDGVSETQFKKVLQ----------EELQSIKTACSKF-Q-DYNP

--SITFAVVQKRHHTRLFRC-----DPDHE------------------------------

---NIPPGTVVDT-VIT-----HPKEF-DFYLCSHLGVKG-TSRPTHY------HILWDE

NEFTSD-----ELQRLVYNLCYTFVRCTKPISIVPPAYYAHLAAYRGRL--YIER-----

------SSESNGGSMN-------------------------------------------P

SSVSRVGPPKTIPLPKL---SDNVK-NLMFYC----------------------------

------

>AGO1_NP_001246314_Drosophila_melanogaster

---------------------------------------FRVT-IKWQAQ--VSLFNLEE

ALEG--RTR-------QIPYD--------AILALD-VVMRHLPSMT--YTPV--------

--------------------------GRSFFSSPE----GYYHP-----LGG--------

GREVWF---------------GFHQSVRP--SQWK---MMLN--I---------------

---DVSATAFYK------------------------------------------------

--AQPVIDFMCEVLDI---------RDINEQRKPLTDSQRVKFTKEIKGLKIEITHC-GQ

M-----RRKYRVCNVT--RRP----A-QMQSFPLQ------LENGQTVECT----VAKYF

--------LD-KYRMKLRY------PHL-PCLQV-------------------------G

QEHKH----------TYL------------------PLEVCNI---VAGQRCIKKL----

-----------TDMQTSTMIK----------ATARSAPDRE-------------REINNL

VKRADF---------NNDSYVQEFGLTISNSM-M--EVRGRVLPPPKLQYGGRVSTGLTG

QQLFPPQNKVSLA-SPNQGV-WDM--R-GKQFF-------TG--VEIRIWAIACFAPQRT

V--------RED-----ALRNFTQQLQKISNDAG--------------------------

--------------------------MPIIGQPCFCKYATGP--------DQVEPMFRYL

KITF----------------PGLQLVVVVLPGKT-PV---YAEVKRVGDTV--LGMATQC

VQA--KNVNKTSP-------QTLSNLCLKINVKLGGINSILVPSIR---PKVF--NEPVI

FLGADVT-HPPA----GDNKKPSIAAVV---GSMD-AHPSRYAATVRVQ-QHRQEI----

------IQEL--------------------SSMVRELLIMF--YKSTGG-----------

-------YKPHRIILYRDGVSEGQFPHVLQ----------HELTAIREACIKL-EPEYRP

--GITFIVVQKRHHTRLFCA-----EKKEQ--SGKSG-----------------------

---NIPAGTTVDV-GIT-----HPTEF-DFYLCSHQGIQG-TSRPSHY------HVLWDD

NHFDSD-----ELQCLTYQLCHTYVRCTRSVSIPAPAYYAHLVAFRARY--HLVEK----

------EHDSGEGSHQ-------------------------------------------S

GCSEDRTPGAMARAIT----VHADTKKVMYFA----------------------------

------

>AGO2_NP_001158095_Homo_sapiens

---------------------------------------FKVS-IKWVSC--VSLQALHD

ALSG--RLPSVPFE---------------TIQALD-VVMRHLPSMR--YTPV--------

--------------------------GRSFFTASE----GCSNPLG-------------G

GREVWF---------------GFHQSVRP--SLWK---MMLN--I---------------

---DVSATAFYK------------------------------------------------

--AQPVIEFVCEVLDF---------KSIEEQQKPLTDSQRVKFTKEIKGLKVEITHC-GQ

M-----KRKYRVCNVT--RRP----A-SHQTFPLQ------QESGQTVECT----VAQYF

--------KD-RHKLVLRY------PHL-PCLQV-------------------------G

QEQKH----------TYL------------------PLEVCNI---VAGQRCIKKL----

-----------TDNQTSTMIR----------ATARSAPDRQ-------------EEISKL

MRSASF---------NTDPYVREFGIMVKDEM-T--DVTGRVLQPPSILYGGR-------

--------NKAIA-TPVQGV-WDM--R-NKQFH-------TG--IEIKVWAIACFAPQRQ

C--------TEV-----HLKSFTEQLRKISRDAG--------------------------

--------------------------MPIQGQPCFCKYAQGA--------DSVEPMFRHL

KNTY----------------AGLQLVVVILPGKT-PV---YAEVKRVGDTV--LGMATQC

VQM--KNVQRTTP-------QTLSNLCLKINVKLGGVNNILLPQGR---PPVF--QQPVI

FLGADVT-HPPA----GDGKKPSIAAVV---GSMD-AHPNRYCATVRVQ-QHRQEI----

------IQDL--------------------AAMVRELLIQF--YKST-R-----------

-------FKPTRIIFYRDGVSEGQFQQVLH----------HELLAIREACIKL-EKDYQP

--GITFIVVQKRHHTRLFCT-----DKNER------------------------------

---------------------------------------G-TSRPSHY------HVLWDD

NRFSSD-----ELQILTYQLCHTYVRCTRSVSIPAPAYYAHLVAFRARY--HLVDK----

------EHDSAEGSHT-------------------------------------------S

GQSNGRDHQALAKAVQV---HQDTL-RTMYFA----------------------------

------

>AGO4_NP_060099_Homo_sapiens

---------------------------------------FKVS-VQWVSV--VSLQLLLE

ALAG--HLNEVPDD---------------SVQALD-VITRHLPSMR--YTPV--------

--------------------------GRSFFSPPE----GYYHPLG-------------G

GREVWF---------------GFHQSVRP--AMWN---MMLN--I---------------

---DVSATAFYR------------------------------------------------

--AQPIIEFMCEVLDI---------QNINEQTKPLTDSQRVKFTKEIRGLKVEVTHC-GQ

M-----KRKYRVCNVT--RRP----A-SHQTFPLQ------LENGQAMECT----VAQYF

--------KQ-KYSLQLKY------PHL-PCLQV-------------------------G

QEQKH----------TYL------------------PLEVCNI---VAGQRCIKKL----

-----------TDNQTSTMIK----------ATARSAPDRQ-------------EEISRL

VKSNSMVG-------GPDPYLKEFGIVVHNEM-T--ELTGRVLPAPMLQYGGR-------

--------NKTVA-TPNQGV-WDM--R-GKQFY-------AG--IEIKVWAVACFAPQKQ

C--------RED-----LLKSFTDQLRKISKDAG--------------------------

--------------------------MPIQGQPCFCKYAQGA--------DSVEPMFKHL

KMTY----------------VGLQLIVVILPGKT-PV---YAEVKRVGDTL--LGMATQC

VQV--KNVVKTSP-------QTLSNLCLKINAKLGGINNVLVPHQR---PSVF--QQPVI

FLGADVT-HPPA----GDGKKPSIAAVV---GSMD-GHPSRYCATVRVQ-TSRQEISQEL

LYSQEVIQDL--------------------TNMVRELLIQF--YKST-R-----------

-------FKPTRIIYYRGGVSEGQMKQVAW----------PELIAIRKACISL-EEDYRP

--GITYIVVQKRHHTRLFCA-----DKTER--VGKSG-----------------------

---NVPAGTTVDS-TIT-----HPSEF-DFYLCSHAGIQG-TSRPSHY------QVLWDD

NCFTAD-----ELQLLTYQLCHTYVRCTRSVSIPAPAYYARLVAFRARY--HLVDK----

------DHDSAEGSHV-------------------------------------------S

GQSNGRDPQALAKAVQ----IHHDTQHTMYFA----------------------------

------

>AGO3_NP_079128_Homo_sapiens

---------------------------------------FKVS-IKFVSR--VSWHLLHE

VLTG--RTLPEPLELDKPISTN-------PVHAVD-VVLRHLPSMK--YTPV--------

--------------------------GRSFFSAPE----GYDHP-----LGG--------

GREVWF---------------GFHQSVRP--AMWK---MMLN--I---------------

---DVSATAFYK------------------------------------------------

--AQPVIQFMCEVLDI---------HNIDEQPRPLTDSHRVKFTKEIKGLKVEVTHC-GT

M-----RRKYRVCNVT--RRP----A-SHQTFPLQ------LENGQTVERT----VAQYF

--------RE-KYTLQLKY------PHL-PCLQV-------------------------G

QEQKH----------TYL------------------PLEVCNI---VAGQRCIKKL----

-----------TDNQTSTMIK----------ATARSAPDRQ-------------EEISRL

VRSANY---------ETDPFVQEFQFKVRDEM-A--HVTGRVLPAPMLQYGGR-------

--------NRTVA-TPSHGV-WDM--R-GKQFH-------TG--VEIKMWAIACFATQRQ

C--------REE-----ILKGFTDQLRKISKDAG--------------------------

--------------------------MPIQGQPCFCKYAQGA--------DSVEPMFRHL

KNTY----------------SGLQLIIVILPGKT-PV---YAEVKRVGDTL--LGMATQC

VQV--KNVIKTSP-------QTLSNLCLKINVKLGGINNILVPHQR---PSVF--QQPVI

FLGADVT-HPPA----GDGKKPSIAAVV---GSMD-AHPSRYCATVRVQ-RPRQEI----

------IQDL--------------------ASMVRELLIQF--YKST-R-----------

-------FKPTRIIFYRDGVSEGQFRQVLY----------YELLAIREACISL-EKDYQP

--GITYIVVQKRHHTRLFCA-----DRTER--VGRSG-----------------------

---NIPAGTTVDT-DIT-----HPYEF-DFYLCSHAGIQG-TSRPSHY------HVLWDD

NCFTAD-----ELQLLTYQLCHTYVRCTRSVSIPAPAYYAHLVAFRARY--HLVDK----

------EHDSAEGSHV-------------------------------------------S

GQSNGRDPQALAKAVQ----IHQDTLRTMYFA----------------------------

------

>AGO1_NP_001304051_Homo_sapiens

---------------------------------------FKVS-IKWLAI--VSWRMLHE

ALVS--GQIPVPLE---------------SVQALD-VAMRHLASMR--YTPV--------

--------------------------GRSFFSPPE----GYYHPLG-------------G

GREVWF---------------GFHQSVRP--AMWK---MMLN--I---------------

---DVSATAFYK------------------------------------------------

--AQPVIEFMCEVLDI---------RNIDEQPKPLTDSQRVRFTKEIKGLKVEVTHC-GQ

M-----KRKYRVCNVT--RRP----A-SHQTFPLQ------LESGQTVECT----VAQYF

--------KQ-KYNLQLKY------PHL-PCLQV-------------------------G

QEQKH----------TYL------------------PLEVCNI---VAGQRCIKKL----

-----------TDNQTSTMIK----------ATARSAPDRQ-------------EEISRL

MKNASY---------NLDPYIQEFGIKVKDDM-T--EVTGRVLPAPILQYGGR-------

--------NRAIA-TPNQGV-WDM--R-GKQFY-------NG--IEIKVWAIACFAPQKQ

C--------REE-----VLKNFTDQLRKISKDAG--------------------------

--------------------------MPIQGQPCFCKYAQGA--------DSVEPMFRHL

KNTY----------------SGLQLIIVILPGKT-PV---YAEVKRVGDTL--LGMATQC

VQV--KNVVKTSP-------QTLSNLCLKINVKLGGINNILVPHQR---SAVF--QQPVI

FLGADVT-HPPA----GDGKKPSITAVV---GSMD-AHPSRYCATVRVQ-RPRQEI----

------IEDL--------------------SYMVRELLIQF--YKST-R-----------

-------FKPTRIIFYRDGVPEGQLPQILH----------YELLAIRDACIKL-EKDYQP

--GITYIVVQKRHHTRLFCA-----DKNER--IGKSG-----------------------

---NIPAGTTVDT-NIT-----HPFEF-DFYLCSHAGIQG-TSRPSHY------YVLWDD

NRFTAD-----ELQILTYQLCHTYVRCTRSVSIPAPAYYARLVAFRARY--HLVDK----

------EHDSGEGSHI-------------------------------------------S

GQSNGRDPQALAKAVQV---HQDTL-RTMYFAXRQNAVTSLDRRKLSKPQELCHPNPEEA

RRREVG

>AGO5_NP_850110_Arabidopsis_thaliana

---------------------------------------FKVA-VKNVTS--TDLYQLQQ

FLDR--KQREAPYD---------------TIQVLD-VVLRDKPSND--YVSV--------

--------------------------GRSFFHTSL----GKDARDGRGELG--------D

GIEYWR---------------GYFQSLRL--TQMG---LSLN--I---------------

---DVSARSFYE------------------------------------------------

--PIVVTDFISKFLNI------------RDLNRPLRDSDRLKVKKVLRTLKVKLLHWNG-

------TKSAKISGIS--SLP----I-RELRFTLE----------DKSEKT----VVQYF

--------AE-KYNYRVKY------QAL-PAIQT-------------------------G

SDTRP----------VYL------------------PMELCQI---DEGQRYTKRL----

-----------NEKQVTALLK----------ATCQRPPDRE-------------NSIKNL

VVKNNY----------NDDLSKEFGMSVTTQL-A--SIEARVLPPPMLKYHDS-------

--------GKEKMVNPRLGQ-WNMI---DKKMV-------NG--AKVTSWTCVSFSTRID

R---------------GLPQEFCKQLIGMCVSKG--------------------------

--------------------------MEFKPQPAIPFISCPP--------EHIEEALLDI

HKRA----------------PGLQLLIVILPDVT-GS---YGKIKRICETE--LGIVSQC

CQP--RQVNKLNK-------QYMENVALKINVKTGGRNTVLNDAIRRNIPLIT--DRPTI

IMGADVT-HPQP----GEDSSPSIAAVV---ASMDWPEINKYRGLVSAQ-AHREEI----

------IQDL-------YKLVQDPQRGLVHSGLIREHFIAF--RRAT-G-----------

-------QIPQRIIFYRDGVSEGQFSQVLL----------HEMTAIRKACNSL-QENYVP

--RVTFVIVQKRHHTRLFPE-----QHGNRDMTDKSG-----------------------

---NIQPGTVVDT-KIC-----HPNEF-DFYLNSHAGIQG-TSRPAHY------HVLLDE

NGFTAD-----QLQMLTNNLCYTYARCTKSVSIVPPAYYAHLAAFRARY--YMESEMSDG

-----------------------------------------------------------G

SSRSRSSTTGVGQVISQLPAIKDNVKEVMFYC----------------------------

------

>AGO10_NP_199194_Arabidopsis_thaliana

---------------------------------------YKVA-IKFVAR--ANMHHLGE

FLAG--KRADCPQE---------------AVQILD-IVLRELSVKR--FCPV--------

--------------------------GRSFFSPDI----KTPQRLG-------------E

GLESWC---------------GFYQSIRP--TQMG---LSLN--I---------------

---DMASAAFIE------------------------------------------------

--PLPVIEFVAQLLGK------------DVLSKPLSDSDRVKIKKGLRGVKVEVTHRANV

------RRKYRVAGLT--TQP----T-RELMFPVD----------ENCTMKS---VIEYF

--------QE-MYGFTIQH------THL-PCLQV-------------------------G

NQKKA----------SYL------------------PMEACKI---VEGQRYTKRL----

-----------NEKQITALLK----------VTCQRPRDRE-------------NDILRT

VQHNAY---------DQDPYAKEFGMNISEKL-A--SVEARILPAPWLKYHEN-------

--------GKEKDCLPQVGQ-WNMM---NKKMI-------NG--MTVSRWACVNFSRSVQ

E---------------NVARGFCNELGQMCEVSG--------------------------

--------------------------MEFNPEPVIPIYSARP--------DQVEKALKHV

YHTSMNKTKG----------KELELLLAILPDNN-GS--LYGDLKRICETE--LGLISQC

CLT--KHVFKISK-------QYLANVSLKINVKMGGRNTVLVDAISCRIPLVS--DIPTI

IFGADVT-HPEN----GEESSPSIAAVV---ASQDWPEVTKYAGLVCAQ-AHRQEL----

------IQDL-------YKTWQDPVRGTVSGGMIRDLLISF--RKAT-G-----------

-------QKPLRIIFYRDGVSEGQFYQVLL----------YELDAIRKACASL-EPNYQP

--PVTFIVVQKRHHTRLFAN-----NHRDKNSTDRSG-----------------------

---NILPGTVVDT-KIC-----HPTEF-DFYLCSHAGIQG-TSRPAHY------HVLWDE

NNFTAD-----GIQSLTNNLCYTYARCTRSVSIVPPAYYAHLAAFRARF--YLEPEIMQD

NGS--------------------------------------------------------P

GKKNTKTTTVGDVGVKPLPALKENVKRVMFYC----------------------------

------

>AGO1_NP_001185169_Arabidopsis_thaliana

---------------------------------------FKVV-IKLVAR--ADLHHLGM

FLEG--KQSDAPQE---------------ALQVLD-IVLRELPTSRIRYIPV--------

--------------------------GRSFYSPDI----GKKQSLG-------------D

GLESWR---------------GFYQSIRP--TQMG---LSLN--I---------------

---DMSSTAFIE------------------------------------------------

--ANPVIQFVCDLLNR------------DISSRPLSDADRVKIKKALRGVKVEVTHRGNM

------RRKYRISGLT--AVA----T-RELTFPVD---------ERNTQKS----VVEYF

--------HE-TYGFRIQH------TQL-PCLQV-------------------------G

NSNRP----------NYL------------------PMEVCKI---VEGQRYSKRL----

-----------NERQITALLK----------VTCQRPIDRE-------------KDILQT

VQLNDY---------AKDNYAQEFGIKISTSL-A--SVEARILPPPWLKYHES-------

--------GREGTCLPQVGQ-WNMM---NKKMI-------NG--GTVNNWICINFSRQVQ

D---------------NLARTFCQELAQMCYVSG--------------------------

--------------------------MAFNPEPVLPPVS-----------ARPEQVEKVL

KTRYHDATSKLSQG------KEIDLLIVILPDNN-GS--LYGDLKRICETE--LGIVSQC

CLT--KHVFKMSK-------QYMANVALKINVKVGGRNTVLVDALSRRIPLVS--DRPTI

IFGADVT-HPHP----GEDSSPSIAAVV---ASQDWPEITKYAGLVCAQ-AHRQEL----

------IQDL-------FKEWKDPQKGVVTGGMIKELLIAF--RRST-G-----------

-------HKPLRIIFYRDGVSEGQFYQVLL----------YELDAIRKACASL-EAGYQP

--PVTFVVVQKRHHTRLFAQ-----NHNDRHSVDRSG-----------------------

---NILPGTVVDS-KIC-----HPTEF-DFYLCSHAGIQG-TSRPAHY------HVLWDE

NNFTAD-----GLQSLTNNLCYTYARCTRSVSIVPPAYYAHLAAFRARF--YMEPETSDS

GSMASGSMARGGGMAG-------------------------------------------R

STRGPNVNAAVRPLPAL----KENVKRVMFYC----------------------------

------

>XP_001456124_Paramecium_tetraurelia

----------------------------------GNETNQDTATLKLVGK----IENKAD

LNII-------------------------ISRLFK-QVIRSQMQMV-SVGNK--------

--------------------------GQKLFWSSR----AQQFKD--------------Q

NLEIWP---------------GVECIFRP--GEGGAQNPTLV--I---------------

---DCAFKMLRY------------------------------------------------

--RSAL-----------------------------EELNQTRNPACIQDQIVMTTYN---

------KKFYKVEAVD--VNL----K-PASTFTNE----------KGETIS----FAQYY

--------EQ-RYKVKVDG-------NQ-PLIRA-------------------------T

VRSKQDKTEKT----IHL------------------IPQLCQL----TGLTDAIRN----

-----------DFNAMKNLAE----------LTK-----EM-------------KMAQEF

ANQLASTEIVNKGSGTKRQIFKEWGVEINPGS-M--DVPARRIHPGNMLMGNG-------

---------LKLDLSSPQTN-LDR--QTQTQMF-------STPPQQLILGIIYNKKTGQQ

T-----------------MDSLMQNFQAACNDFK--------------------------

--------------------------FQAFMAPKVFPIEQ----------DRDEDLERVL

DGFQKQAEANK---------AKVGFLLFLLPGQK-KKARLYKTAKKISMQK--FGCASQV

VVE--KTLAKNTR-------SIVNKILIQLNAKVGGTPWAIDS------LPTTFQNQPTM

ICGTDCF---------VKSGRKNQLAFC---STVD-RNLSRYYSQVVTSGEFSQHL----

------------------------------QQVFKASLLAF--KEQN-G-----------

-------IFPKLIIIYRDGVGDGQQAVVLA----------NELPQYKQALEEL--QITDT

--KISLVVCNKRVSAKFY--------TGGN---ARPD-----------------------

---NPQPGTCVDNPKVV-----EQSNP-NFYLISQVTRQG-TVTPSLY------KIIHSD

QAGLDD-----DIKVLTFKLCWLFYNFTGSIKIPAPVRYAHCLCNFIGD--NYD------

------------------------------------------------------------

----------DRDQVKFLPLPDLVKQKVLFYI----------------------------

------

>XP_001454540_Paramecium_tetraurelia

----------------------------------GNETNQDTATLKLVGK----IENKAD

LNII-------------------------ISRLFK-QVIRSQLQMV-SVGNK--------

--------------------------GQKLFWSSR----AQQFKD--------------Q

NLEIWP---------------GVECIFRP--GEGGAQNPTLV--I---------------

---DCAFKMLRY------------------------------------------------

--RSAL-----------------------------EELNQTRNPSCIQDQIVMTTYN---

------KKFYKVEAVD--VNL----K-PASTFTNE----------KGETIS----FAQYY

--------EQ-RYKVKVDG-------NQ-PLIRA-------------------------T

VRSKQDKTEKT----IHL------------------IPQLCQL----TGLTDAIRN----

-----------DFNAMKNLAV----------VTKPGADQRM-------------KMAQEF

ANQLANTEIVNKKAGTKRQIFKEWGVEINPGS-M--DVPARRIHPGNMLMGNG-------

---------LKLDLSSPQTN-LDR--QTQTQMF-------STPPQQLILGIIYNKKTGQQ

T-----------------MDSLMQNFQAACNDFK--------------------------

--------------------------FQAFMAPKVFPIEQ----------DRDEDLERVL

DGFQKQAEANK---------AKVGFLLFLLPGQK-KKARLYKTAKKISMQK--FGCASQV

VVE--KTLAKNTR-------SIVNKILIQLNAKVGGTPWAIDS------LPLTFQNQPTM

ICGTDCF---------VKSGRKNQLAFC---STVD-RNLSRYYSQVVTSGEFSQHL----

------------------------------QSVFKASLLAF--KEQN-G-----------

-------IFPKLVIIYRDGVGDGQQAVVLA----------NELPQYKQALEEL--QITDT

--KISVVVCNKRVSAKFY--------TGGN---ARPD-----------------------

---NPQPGTCVDNPKVV-----EQSNP-NFYLISQVTRQG-TVTPSLY------KIIHSD

QAGLDD-----DIKVLTFKLCWLFYNFTGSIKIPAPVRYAHCLCNFIGD--NYD------

------------------------------------------------------------

----------DRDQVKFLPLPDLVKQKVLFYI----------------------------

------

>XP_001440628_Paramecium_tetraurelia

---------------------------------------YYLE-IKKVKV--INMQDLEN

PNPI--EADK-------------------PLMFFN-YLIKDFFKNL-KYQEH--------

--------------------------GKTRKFHDI----SRRQPIPE------------T

ELSVIP---------------GFAVNFQT--NEQG---FSFK--I---------------

---DISHRIIRT------------------------------------------------

--TTVL-QYIDQIWKE----------------QSNKQCREKYVTHVMTKQLVLANYG---

-----TYRTYSVDNLL--FDQ----NTAEYSIELA------DSKGKQQFIT----MDQYF

--------KQ-RYNYDIKN------KKA-PLLVI-----------------------EDT

KTNRK----------VYL------------------VAELCIM----AGVPDNL------

-----------NEFTRKQIQK----------ECQTNPKDRF-------------EKIKKM

LFDLIN--RQPQTGLTMQKLKEEFGIDINSTP-Q--YVKAKQLRPPNIVFGNQ-------

----------NVMPQGDKEN-FSFNCNNQSAFI-------KR--YEVRIAIISPYDFDCE

E--------------------FTQNIHELIQKYG--------------------------

--------------------------IKMGVQTKCHYLS-----------GQRQNYIAEI

EQTIMRNVP-----------HTTNMIVIVLNRYM-KN--CYAPLKKMCMSE-KFGVLTQM

VSS--DSISKQKKGLF----SIIQKLAIQMMDKVGNCLWTVQL------PQKW--PDNIM

IVSVIIE-------------KGKFAGML---SSLD-KTYSRYYSQMSTK-IVEKCL----

------IKDI--------------------GDMMKQSFLQF--KQEN-G-----------

-------CFPQKIIYFRDGLSEESISHLLQ----------YETREIVLAANQL-CQ-FGD

--QIIIINIQNTNITKLF--------REER---DQYF-----------------------

---NAPAGTVVDS-EIT-----TKN-Y-EFILVPVYSSRG-CPRPILY------RIIYDT

IKIPME-----QLQDFVYGQCYNYQNWTGSIKLPAIVKNCQKMIKFLVE--ILQTE----

------------------------------------------------------------

--------------------PINKLRSSFYYL----------------------------

------

>AGO3_NP_001036629_Drosophila_melanogaster

---------------------------------FDGNTLYLPI-LLPNKM--TVFISKAE

DVEL--QIRILYKKKEEMRN---------CTQLYN-ILFDRVMKVL-NYVKF--------

--------------------------DRKQFDPSR----PKIIPL--------------A

KLEVWP---------------GYVTAVDE--YKGG---LMLC--C---------------

---DVSHRILCQ------------------------------------------------

--KTVL-EMLVDLYQQ------------------NVEHYQESARKMLVGNIVLTRYN---

------NRTYKINDIC--FDQ----N-PTCQFEIK----------TGCTS-----YVEYY

--------KQ-YHNINIKD------VNQ-PLIYS-------------------------I

KKSRGIPAERENLQFCLI-------------------PELCYL----TGLRDEVRS----

-----------DNKLMREIAT----------FTRVSPNQRQ-------------MALNKF

YENVSNTP-------AAQEILNSWGLSLTNNS-N--KISGRQMDIEQIYFSKI-------

------------SVSAGRSAEFSKHAV-TNEML-------KV--VHLSKWIIIHLRNYRQ

A-----------------ATSLLDNMKQACESLG--------------------------

--------------------------MNISN-PTMISLDH----------DRIDAYIQAL

RRNIT---------------MNTQMVVCICHNRR-DD--RYAAIKKICCSE--IPIPSQV

INA--KTLQNDLKIR-----SVVQKIVLQMNCKLGGSLWTVKI------P-----FKNVM

ICGIDSY-HDPS------NRGNSVAAFV---ASIN-SSYSQWYSKAVVQ-TKREEI----

------VNGL--------------------SASFEIALKMY--RKRN-G-----------

-------KLPTNIIIYRDGIGDGQLYTCLN----------YEIPQFEMVCGNR-I-----

--KISYIVVQKRINTRIF--------SGSG---IHLE-----------------------

---NPLPGTVVDQ-HIT-----KSNMY-DFFLVSQLVRQG-TVTPTHY------VVLRDD

CNYGPD-----IIQKLSYKLCFLYYNWAGTVRIPACCMYAHKLAYLIGQ--SIQRD----

------------------------------------------------------------

--------------------VAEALSEKLFYL----------------------------

------

>Tetrahymena_thermophila_XP_001032516

---------------------------------RVDDQTYSLS-LTFTKL--FEVNEESA

QNKD--KTTPNYKKGNK------------VTQVLN-VILKNIIDQE-GFKQI--------

-------------------------GRNSQFFDLQ----SRADVVIKNQNNT-------S

TLEVYK---------------GITFKVSP--TQQG---LYLN--S---------------

---DYLCRILRK------------------------------------------------

--ETAQ-QYLT-------------------------------ARHDIEGQSVLTKYN-N-

------YRTYIIDRVD--YSK----N-PRSTFLYR---------KTNLQIS----YAEYY

--------FQ-NYQIEIRD------MQQ-PLLVS-------------------------K

IKYREADTNILKIQEIFL------------------IPELCYM----TGLTDEQRK----

-----------NKNAMKEIAT----------HTKLTPEQKY-------------SKSVQY

CQL------------FGRKTIKS-GIRISHTD-N--KIDAIQLKAPKITFGGK-------

------------SFIPEHGGNFET----RAPTQ-------DQ--VQFKDWAIIHHKNDEK

E-----------------VYKFIDLLKNASQAYQ--------------------------

--------------------------INVNDKPQFFAPED----------FNPKNWIRLL

DKDFRKN-------------GVPQFIVTFSNAEK-NP-SLYREMKKFFSSEGGVGIESQH

VTP--RALQKNGK-------SVASKIALQIASKLGKRIWSVET------PVGI--NQNTM

IVGIETS-MKKI-------RNQQVIGVV---ASIN-KDFNKFYSQVDFR-NGNDIK----

------LPTL--------------------SKIISSAIEAY--SKNT-K-----------

-------TVPEEIIIYRQGLGEGQIQYSHQ----------LEIQAIQNGFINF-KQGYSP

--RFAFFQVNRKISEKFYQQ-----LYNER---VQIS-----------------------

---NPPSGTIVAS-ELT------QNNF-EFFMAAQNCNSG-VCTPTKY------TCLFNN

TNLKED-----QFWQLTYFQTFNYYNWQGPIRVPAVMKYAEKLAKFTSE--TLEGV----

------------------------------------------------------------

--------------------ANNELINSLYYI----------------------------

------

>Tetrahymena_thermophila_XP_012652907

---------------------------------NVDGQKYSIL-LRFTKS--FEVNDESA

QNKD--KTSVNFKKGNQ------------VTQLLN-IILKSIMRDE-KFQEI--------

-------------------------GKNSKFYDVN----SKSDIIIKSRDGST------S

ALEAYK---------------GFTFNVTP--TQDR---LYLM--V---------------

---DYCSRIIRK------------------------------------------------

--ETAL-EYL------------------------------KNNNNQLSGLSVITKYN-N-

------YQTYLIDRVD--DRK----T-PSSTFLNQ---------KTKTQIS----FAQYY

--------KE-RYNIQIRD------MRQ-PLLVH-------------------------K

RKYREPNTNIQKVEEVYL------------------IPELCNM----TGMTDEQRE----

-----------NFQAMKEVAT----------HTKLTPQERY-------------QNSVNN

CKFLSN-------------KTSSSGIKIDERS-N--LIDAVQLRAPKVTLGGN-------

-----------KTANTERGN-FDM----RAPTL-------DK--IEFKDWSLICNKKDDQ

S-----------------ADNFIDTLKKASQTFG--------------------------

---------------------------ITVNDPCFLAQRD----------WNANNWIRSL

DEDFSNN-------------GLPQFVLSFGQANS-GL---YSGLKRFLTSE--VGIESQH

VNP--RSLQKNGM-------SVASKIALQIASKLGRRIWQVET------PTGI--DKNTM

IIGIETS--------MKKINRQQVIGVV---ASID-KDFTKYFSDVEIR-KENDTT----

------FPTL--------------------SKIVTKAIQAY--VKNT-K-----------

-------VVPAEVIIYRQGLGEGQIQQSFN----------LEIKAIQNGFSNF-KKDYNP

--RLAFFQVNRKIGQKFYQQ-----PQGDK---SEVS-----------------------

---NPASGTIVAS-QVV------QNNF-EFFMAAQNCNSG-VCTPTKY------TCLYNN

TNLKED-----QIWQLTYYQTFNYYNWQGPVRVPAAMKYAEKLAKFVSD--TIQES----

------------------------------------------------------------

--------------------ANENLANSLFYL----------------------------

------

>Tetrahymena_thermophila_XP_001470773

---------------------------------NVDGQKYSIL-LRFTKS--FEVNDESA

QNKD--KTSINFKKGNQ------------VTQLLN-IILKSIMRDE-KFQEI--------

-------------------------GKNSKFYDVN----SKSDIIIKSRDGST------S

ALEAYK---------------GFTFNVTP--TQDR---LYLM--V---------------

---DYCSRIIRK------------------------------------------------

--ETAL-EYL------------------------------KNNNNQLSGLSVITKYN-N-

------YQTYLIDRVD--DKK----T-PSSTFLNQ---------KTKTQIS----FAQYY

--------KE-RYNIQIRD------MKQ-PLLVH-------------------------K

RKYREPNTNIQKIEEVYL------------------IPELCNM----TGMTDEQRE----

-----------NFQAMKEVAT----------HTKLTPNERY-------------QNSVKN

CKFLSN-------------KTSSSGIKIDEKS-N--LIDAVQLRAPKVTLGGN-------

-----------KTANTERGN-FDM----RAPTL-------DK--IEFKDWSLICNKKDDQ

S-----------------ADNFIDTLKKASQTFG--------------------------

---------------------------ITVNDPCFLAQRD----------WNANNWIKTL

DEDFSNN-------------GLPQFVLSFGQANS-GL---YSGLKRFLTSE--VGIESQH

VNP--RSLQKNGM-------SVASKIALQIASKLGRRIWQVET------PTGI--DKHTM

IIGIETS--------MKKINKQQVIGVV---ASID-KDFTKYFSDVEIR-KENDTT----

------FPTL--------------------SKIVTKAIQAY--VKNT-K-----------

-------SVPAEVIVYRQGLGEGQIQQSCN----------FEIKAIQNGFINF-KKDYNP

--RLAFFQVNRKIGQKFYQQ-----PQGDK---SEVS-----------------------

---NPASGTIVAS-QVV------QNNF-EFFMAAQNCNSG-VCTPTKY------TCLYNN

TNLKED-----QIWQLTYYQTFNYYNWQGPVRVPAAMKYAEKLAKFVSD--TIQES----

------------------------------------------------------------

--------------------ANENLANSLFYL----------------------------

------

>Tetrahymena_thermophila_XP_001470772

---------------------------------NVDGQKYSIL-LRFTKS--FEVNDESA

QNKD--KTSVNFKKGNQ------------VTQLLN-IILKSIMRDE-KFQEI--------

-------------------------GKNSKFYDVN----SKSDIIIKSRDGST------S

ALEAYK---------------GFTFNVTP--TQDR---LYLM--V---------------

---DYCSRIIRK------------------------------------------------

--ETAL-EYL------------------------------KNNNNQLSGLSVITKYN---

-----NYQTYLIDRVD--DKK----T-PSSTFLNQ---------KTKTQIS----FAQYY

--------KE-RYNIQIRD------MKQ-PLLVH-------------------------K

RKYREPNTNIQKIEEVYL------------------IPELCNM----TGMTDEQRE----

-----------NFQAMKEVAT----------HTKLTPNERY-------------QNSVKN

CKFLSN-------------KTSSSGIKIDEKS-N--LIDAVQLRAPKVTLGGN-------

-----------KTANTERGN-FDM----RAPTL-------DK--IEFKDWSLICNKKDDQ

S-----------------ADNFIDTLKKASQTFG--------------------------

---------------------------ITVNDPCFLAQRD----------WNANNWIKTL

DEDFSNN-------------GLPQFVLSFGQANS-GL---YSGLKRFLTSE--VGIESQH

VNP--RSLQKNGM-------SVASKIALQIASKLGRRIWQVET------PTGI--DKNTM

IIGIETS--------MKKINKQQVIGVV---ASID-KDFTKYFSDVEIR-KENDTT----

------FPTL--------------------SKIVTKAIQAY--IKNT-K-----------

-------VVPAEVIVYRQGLGEGQIQQSCN----------FEIKAIQNGFSNF-KKDYNP

--RLAFFQVNRKIGQKFYQQ-----PQGDK---SEVS-----------------------

---NPASGTIVAS-QVV------QNNF-EFFMAAQNCNSG-VCTPTKY------TCLYNN

TNLKED-----QIWQLTYYQTFNYYNWQGPVRVPAAMKYAEKLAKFVSD--TIQES----

------------------------------------------------------------

--------------------ANENLANSLFYL----------------------------

------

>Tetrahymena_thermophila_XP_001015192

---------------------------------NVDGQKFSIL-LRFTKT--FEVNDESA

QNLD--KTSPNFKKGNQ------------VTQLLN-IILKSIMRDE-KFQEI--------

-------------------------GKNSKFYNVN----SRNEIVIKGRDGNT------S

ALEAYK---------------GFTFNVTP--TQDK---LYLM--V---------------

---DYCSRIIRK------------------------------------------------

--ETAL-EYL------------------------------KNNNNQLSGLSVITKYN-N-

------YQTYLIDRVD--GQK----T-PSSTFLNS---------KTNTQIS----FAQYY

--------KE-RYNIQIRD------MRQ-PLLVH-------------------------K

RKYREANTNIQKVEEVYL------------------IPEICNM----TGMTDEQRE----

-----------NFQAMKEVAT----------HTKLTPQERY-------------QNSVNN

CRFLSSK-------------TSSSGIRIDERS-N--LIDAVQLRAPKVTLGGN-------

----------KTA-NTDRGN-FDM----RAPTL-------DK--IEFKDWTLIYNQKDER

S-----------------VDGFIDTLKKASQTYG--------------------------

--------------------------ITVND-PFFFAQRD----------WNANSWIKSL

DEDFSKN-------------DLPQFVLSFGQAQS-GL---YSGLKKFLTSE--AGIESQH

VNP--RSLQKNGM-------SVASKIALQIASKLGRRIWQVET------PTGI--DKNTM

IIGIETS-MKKI-------KQQQVVGVV---ASID-KDFTKYFSDVEIR-KDNDTT----

------LPTL--------------------SKIVTKAIQAY--VKNT-K-----------

-------SVPAEVIVYRQGLGEGQIQQSCN----------LEIKAIQNGFSNF-KSDFNP

--RLAFFQVNRKIGQKFYQS-----PQGDK---GEVS-----------------------

---NPASGTIVAS-QVV------QNNF-EFFMAAQNCNSG-VCTPTKY------TCLYNN

TNLKED-----QIWQLTYYQTFNYYNWQGPVRVPAAMKYAEKLAKFVSD--TIQEA----

------------------------------------------------------------

--------------------ANENLTNSLFYL----------------------------

------

>Tetrahymena_thermophila_XP_001015193

---------------------------------IVDKQKYSIH-LVFTKS--FEVNDESA

QNLD--KTSPNFKKGNQ------------VTQLLN-IILKSIMRDE-KFLEI--------

-------------------------GKNSKFYEVN----SKSDIIIKSREGST------S

GLEAYK---------------GFTFNVTP--TQDK---IYLM--V---------------

---DYCSRIIRK------------------------------------------------

--ETAL-EYL------------------------------KNNNNQLSGLSVITKYN---

-----NYQTYLIDKVD--DKK----T-PSSTFLNS---------KTNIQIS----FAQYY

--------KE-RYNIQIRD------MKQ-PLLVH-------------------------K

RKYREANTNIQKVEEVYL------------------IPELCNM----TGMTDEQRE----

-----------NFQAMKEVAT----------HTKLTPQERY-------------QNSINN

CRFLSK-------------KTSSSGIKIDERS-N--LIDAVQLRTPKVTLGGN-------

-----------KTANTDRGN-FDM----RTPTL-------DK--IEFKDWSLIYNQKDEK

Y-----------------VDDFVDTLKKASQTYG--------------------------

---------------------------IIVNDPYFFAERD----------SNANNWIKSL

DEDFSKN-------------DLPQFVLSFGQVNS-GF---YSSLKKFLTSE--AGIESQH

VTP--KSLQKNGM-------SVASKIALQIASKLGRRIWQVET------PRGI--DKNTM

IIGIETS--------MKKVKQQQVVGVV---ASID-KDFTKYFSDVEIR-KDNDTT----

------LPTL--------------------SKIVTKAIQAY--VKNT-K-----------

-------SVPAEVIVYRQGLGEGQIQQSFN----------LEIKAIQNGFSNF-KNDFNP

--HLAFFQVNRKIGQKFYQS-----PQGDK---VEVS-----------------------

---NPASGTIVAS-QVV------QNNF-EFFMAAQHCNSG-VCTPTKY------TCLYNN

TNLKED-----QIWQLTYYQTFNYYNWQGPVRVPAAMKYAEKLAKFVSD--TIQEA----

------------------------------------------------------------

--------------------ANENLTNSLFYL----------------------------

------

>XP_001437215_Paramecium_tetraurelia

----------------------------------MNKEKKMILDIKYVKT--INLKEMAQ

FDDQ------------SSINV--------TKQAIN-AILKQLYESR-NMKEL--------

-------------------F------GKGKFYESK----MNEEKNLQKN----------Y

NIGYMK---------------GFRSAFCS--GQSS---PLLQ--I---------------

---DYSVKLI-N------------------------------------------------

--TYPI-SRIISDMEW----------------NYDKHELQLQIRKKLIGQSGLAKYS---

------NRFYRIDDID--FSR----N-PQSMM-------------EDGKTT----YRLYY

--------QQ-RYNITIND------ISQ-PLLVH-------------------------Q

TKKGQ---------KIRL------------------IPELMML----TGLSQAQKT----

-----------NTNIKKFLRP----------ILIVNQNERQ-------------QRIMDE

RGELEY-------------LMKQQNIVLKSNS----KTQAYEIRKPEIYAEGI-------

-----------IN-SFPGGC-FEI----KNKFY-------QQ--SKLENWVLIYNDQEEQ

L-----------------ANYFLRQLISSGNRYG--------------------------

---------------------------LILSQPLRQKVKS----------DQSQDWISCL

EQNFSEK-------------GRPKLVVSLIDQEK-DK-QIYQQLKQYLIAE--EGVSHQN

VTL--QLIENQKFG------AIVPKIIQQIHSKLGNQTWNI--------QKIQEISDNIM

IVGIDVY-HKTV------LGLDSCVGFN---AQFGQQGYANFTKTIIV--RKGKEI----

------NKDV--------------------AMLLEQSLEEY--QNYN-K-----------

-------KLPDTIIIFRDGVGTSQINRLYQ----------EEVETMKEIINNK-YNLKLP

--EFAFIMVNKRINDRFFSQ-----SKENF------------------------------

-------GMIVAD-RVV------SSHF-DYFLIAQQVNQG-TATPTHY------TVLENS

TKWNED-----LFWKFTFYQCFNYRNWCGPVKIPACVQNAHTAAYRVGE--VIKTN----

------------------------------------------------------------

--------------------ASYYLETKLFFL----------------------------

------

>XP_001448808_Paramecium_tetraurelia

----------------------------------LDGKKHCVY-LNFTKA--IDFQYLEG

DNQD--PALMQP-----------------LQQAVN-VQVKKALKDM-GLREL--------

-------------------N------RLSQFFDPK--------SIDANPVKG-------F

PLKVWL---------------GYKTSLKL--TSTK---PQLL--I---------------

---DYASRVLST------------------------------------------------

--QSAL-NFIRSF-----------------------NQNIGQIKDELEGKSVLATYG-N-

------YRLYKISAVD--FKK----T-PKSSFQLE----------DGKQIT----YAQYY

--------QD-RYKIKIQD------MNQ-PLLES-------------------------L

DRRNPDKK-------FYL------------------IPELVYM----TGLTDDQRS----

-----------DFGLMKELAY----------YTKKEPGERL-------------QIIQSL

LS-------------KLQKQLNAQQMTLKSDS----STHAYIVQQPQLTFGNG-------

---------RRLD-ADPTGF-FIL----KDPVF-------QS--AYIKDWVLIYQSRGRQ

D--------DEL------ADDLVADLNQQGGRLG--------------------------

--------------------------IKFDK-PFFINMKG----------NKPQEWIKEL

QAEFKN--------------GLPSLVVSLTDKNR-DT-SLYIGLKDFLLSQGGAGVIHQN

VTT--KSCKNKNKQ------SIASKIAQQISVKLGNPLWVI--------PKVKGISEKIM

IIGMDIY-HKLV------TGKQSCMGFV---AHFDLECKTSFSKTIIM--KSGQEF----

------NQAV--------------------GQTFKEALQAY-FLHYGKK-----------

-------QLPDTILVYRDGVGDSQIQTLMQ----------TELEQIKKVISTY-TAGYNP

--QFAYITINKKISDRFFMT-----RKNNK---SPYI-----------------------

---NPVSGLVVAD-RVT-----SKY-F-DFFLAAQYVTQG-TCTPTHY------QVLDNN

TQFTEE-----MFWQITYYQCFNYWNWTGAVKVPACVQYAHKLAFLVGD--TFQKA----

------------------------------------------------------------

--------------------VHQNLQKQHFYL----------------------------

------

>XP_001435375_Paramecium_tetraurelia

----------------------------------FQGQEYRVL-VEHKKE--YTLQDLSE

TTVN------------------------PVTQSIN-VAVKRSLREM-GMIEI--------

-------------------------GRQSKFYDPR----DIDCNR--------------H

GLKVWR---------------GVKTSFQM--YQGK---PYLQ--I---------------

---DFASRVLRD------------------------------------------------

--QTAL-QFMMSLNTR----------------------NIQDIQNEMIGLSVLAAYG---

-----NCRIYKIDEID--FTV----S-PLHSFQLQ----------DGKSIT----YQEYY

--------KQ-RYNIQIRD------LQQ-PLIVN-------------------------R

DKRNQDKI-------IYL------------------VPELLTM----TGLTDRQRS----

-----------DYRCMQSVAQ----------YTKLTPQQRD-------------NEIYGF

YQ-------------NLRKYLNKQNIQLSDD--Q--NVGGFQLQAPRIFMGNK-------

----------EYQ-TDQSGF-FMI----KDPVF-------QG--SHIRDWFMVYQSRGKN

D--------DDD------VDFLVSELQKQGDRIG--------------------------

--------------------------IRIEK-PYYVVLKD----------NNIQNWMQRL

TAEIGD--------------KPPQLIVTFINEKD-KD-RIYGQMKKYCFQE--HGISHQN

ILS---KFLKSKNPS-----SVASKIAQQMSMKLGNPLWAIPK------PNGI--SDKTM

VIGIDIY-HKLL------TNRRSCMGFV---AYLESECLNTFARPIIM--KEGQEM----

------CHEV--------------------GRITVEAISAY--FERNGKK----------

-------YLPDTIIVFRDGVGNAQIEALKQ----------TEILQMKNAIRSI-NKNYNP

--QFAVIMINKKINDRFFMV-----NGGGGQNQQKQTLS---------------------

---NPPSGSVIAD-KIT------SSNF-DYFITAQYVTQG-TCTPTHY------RVLEND

TNWSEE-----LFWQFTYYQCFNYQNWTGAVRVPSCVQYAHKLAYLIGD--TYQGT----

------------------------------------------------------------

--------------------LHKRLAHLQCCL----------------------------

------

Dicer proteins in afa format:

>Dicerrelated_RNase_III_protein_Dcl1p_Tetrahymena_thermophila

----------------MRNKPKVKSAKLGFKIICHSIKKQYS-----SIKKTKQKKIDK-

-----------GQSKK----RLTAIKQKQGYDIKMD--MQLSQIPEAENLAFQF----DA

KNQCQKTVDYLLSQNL--------------------------------------------

------------------------------------------------------------

------------------------------------------------------------

-------------EQISQCTYIQQPFREIKQKKDTHN-------ESDEESKIYEVYLHQV

YNTENPAY-----VFGFISSV--------------KLPELRELELFLRKDLEDYK-----

------------------------------------------------------------

---------SEVNPCIYQLKF---------------------------------------

------------------------------------------------------------

------------------------------------------------------------

------------------------------------------------------------

-----------------------------------------ISTISLSSSELL-------

------------------------------------------------------------

-SIYNFNTGFFKDFQQEGNQI----SSYIFEYF---------------------------

------------------------------------------------------------

------------------------MQSTISVKFLQVLLTQK-----YIEVELNNF-----

------------------------------------------------------------

--------------------------------------------NNNHVLTNFD------

SKKCDMEKE----------INENPYQFIDFDLIHNFNLQKKQKSIL-------DNK---L

NLATTFINFSQ-------------------------------------------------

------------------------------------------------------------

-----------------------------------------------------DLQMIDE

TSKSDNLDKQNVTIIELINEN-------------------TLTMNQKDQLIENE------

-------NKLQDE------V----IILP----------------TKQIEEFNL-------

------------------------------TFQKEKNKIND------------ISDQEIT

QNL------HDLKKIDV-----DSNNQQENTCSSVVVTEQNGQK-----------QELV-

-----------------NDQNK--Q-----LQEIQ--QSEQDQIPENQ------------

-----MKM------QDPTNQDQQQKP------------RV--------------------

-------------------------------------KLDG--------DLF--------

QESEKLNNISLTLKNEK--------------FRLAYQSN---------------------

IYRHLFLNQDLYQIVEI-------------------------------------------

--------------------------------FDPSNTTIDQFIQSLS-QHTGMSYDSIK

QK-----------------LFLKQDATQKDLQSDTLYDCIYSQ-----------------

------------------------------------------------------------

----------------------------------------PDK-----YLTDKAQMFFA-

------------IRKQFVNFQKKEIQ---QYIQD--------------------------

YQQQLIANNNIQTN-------------Q------------Q-------------------

TQNK----------YNQNTNQISQYHQFNR------RNPLVLCRKINH-LKN--------

--------------------FDFLSDAHI------------------K--------NNKG

V------------------FQ---------------------------------------

------------SCLQKDFSDNKIN--------MSLLPLEHFEQH---------------

---------------------------VLN-------TQLINSHLQA-PSIFALIKDVMI

VRDFACNYFLEN------------------------------------------------

------------------------------------------------------------

--------------------NVDLEL----------------------------------

------------------------------------------------------------

------------------------------------------------------------

------------------------------------------------------------

------------------------------------------------------------

------------------------------------------------------------

-----------LIESFSQIS---YSKTKNLELLEVIGDVVIKYISSL-YLYLHLPN----

----------------------------------QSENALTMI-RTIFINNKYLGI----

SAFRNGLQFYVQTKRPSSYD-WR-FNLFN-------------------------------

----------------------LNSFTEAPQDQKQSFQLSYSNLSDTLESVVGCFYASNQ

FTLNTCIQLIKDLGLQIDQYLLNKFQNQVNGSFVLSDDIIDLMEIDE-------EMTYYE

LQL-------------------QGKKFLQKEFEIQANKKMEQCFEEKIQNEIIQ-NEEN-

-------------------------TQFIWN------------------LIKFYI-----

----------------------------------KDELFYKNKYEVEFQNNTCPNQRRKD

RSEYRKFKQL-HEQTLDIAISGFE--------------KILNYTF--KDKSLLI---KAL

NCHSNSNFEHY-------------------------------------------------

--------FQD-------------------Y-----------------------------

-------------------------------------QI--LEFLGDALIEILVVSHGY-

----HISRNRQKKINS--------------------------------------------

------------------------------------------------------------

------------------------PYFFCEMKSLLLSNDFMSRMAILN-----------K

FHYYALN------------------ISKYQQDEIIQFIEDCNLNTKFKESVSHQ------

------------------------------------------------------------

-------------------ANSPKVLGDLWESVAGAIIM-DS--DISTFVSIYGKLYAPY

IIHFVTFYQKIIF-NVKEKVQLVAQQTYEQLGK--------ITEVVKDTDGQYKVKVT--

--------------------ILIKQI---KVGT-----------GIDYSETLAEKKAFQ-

------DAYDHIFIK----------------QQSNLGLIFQGKQV---------------

-

>Endoribonuclease_Dicer_homolog_1_Chromera_velia

-------PDQAALEDIRDTISNLTRPGFRMDHFDDYLTRQAVT----IERVFEQELVKQS

EKEWSELRRKAQKGEA---EAVGVRKSNAAVPLGEMLGWVSQALSDLKRFFPLSCFDKER

YDLDLRWKEKFKKAEE----------VLDEMGVWGLYHFF--------------------

GEMLGQANEERMNEIWADESEQEKD-----------------------------------

------------------------------------------------------------

--DEVDVEGKGKVAGGASQEEEEEEDSDAEEGEEEEYTDRDDLEEENEEDVRAEAVENGK

KGTKERKKEEIGKNRGGREAQKYLRTLRNEK----LQEGLLQVGPKDRNCWVNDRLDIIT

L-----------------------------------------------------------

----GLMRLRIPDLEERIAEAMKCLQDMGDVCVSDVNKK-NTAWK-----------AVSD

KLLV-----------LRTVLLEEIHFIVGGGRGGEREGVLPKSGLTAAADLSAGEDVKVS

PEAAALKL---------------------TRLDLLRMYWSKSVENKNL------------

------------------------------------------------------------

--ERSDSEGAKKDDDLGFRME-IPRSAAHLVKLPGEAPDLYCPNCLQYLARLPREA----

---------------------------------------GIAAEQKERELKKHDLSGSCS

SSARAENFRFLLRRMRKAYKK----LSDKHPDLLDEATQIARPPLPPPPNPAEVADQVRD

VELRQ-------------------------------------------------------

------------------------EADLKCIIFVNTRYKAR-----LVARFLAALRL---

---------CRVGWVTSAAPGGVGGSTATYGDRMKVR-----------------------

--------------------------------------------EQKRTLDRFRIAAPLH

PDNLQCLTATT--------VIEEGIDIPSCNLVLCFDMPSSPVENI-------QRRGRAR

REGARYLWMKP-------------------------------------------------

----------------------------SGQ-----------------------------

--------------------------------------CGATVTQRNQSLKDAEDDLYLL

TQSETGFGKSAGENRPEDAGICLGALHVKSSGALVPLDRAKVCLERTFGRAGKELPSKWW

DVSGDSYVRLPYLPTRLEAKDKDPNLTKGEREANEKNEEEKKKREKEIEELKELEDEAGV

SVTPDGLLEIRWDGQDPLGDLSIQGTEGAFAIDHSLDINQRSSLRAKVMSRKVLQALVKC

RRL----SDHLLPHTPPLPIP---KRQRMKAGINEQEKTLWREL-----------PDCLL

EPDTFSRLYQRHPMSKAGSVRMYLHHVFIYPS-----REYSDVYKMGG------HFGWWL

R----LWKEKLAVAVPVQSSGDTTSAETATESESVSQRHRKAGKARPSSAAPEKKKRKYQ

SVETYMWGDRQPDSRPPDTFEYSEKAVLMSLAL----LLPQPLRAPISFPAFHPPDCREI

TGGEELKVEIVVAKRSRQTPTGREEEEAPVHFDIDCKKM---------------------

GILKRLKGKYLNLPHID-----------------AFQPTENPVLLNPLALVT--------

-----------------------------AEDPADPLRLQDPCFFPVP-----MTEDPFE

IDW--------TFSEGLWEVRCFGSDPRKGKVPSWLHLMDRFPAEVRELPSLPSDWVE--

----------------------------------------RSEGLDDSSFPAGC------

--------------------------------------PQPETRLARMVQHLSTRGPEP-

------------WV--MREKDRPWL----YWVTT-------------------------R

IEHGVDPFSYGCEA-------------G--------------------------------

TIAD----------YY-EKFRRPAAQVSKT------NRMVLSGDKIKQGPKN--------

----------------------ILFP----------------------------------

------------PVASVLKEK---------------------------------------

------------GERDLNDAHYQAP--------KNLPQFV--TPI---------------

---------------------------PIT-------GPMWNALCWL-PAIVSQVERFAK

IQDLHVWLTQLCNDGPLRTPRG--------------------------------------

-------------------------------------------------------LIGCE

GEGGGLEDFLSKIRVQHPNHPIASEL----------------------------------

------------------------------------------------------------

------------------------------------------------------------

---------KKRMDSLREYEGEKSLETHLKNHLTKLIPDTPAEPDMFPVVARRASTLLSC

GPRVQVEFDLLREAMTSP------------------------------------------

------------------------------------------------------------

-----------AMDFMGTNH---PEQGINYQRLEWLGDSVLKFLASL-GVFFDCDNTDRG

PRVVARDEEAIVAAEIPDDEELHMRTASLVALRETDEGKLSYR-RQVMISNSFLEK----

HSKRHRLYSFLVSRPFTHRT----RGGQLLTEQRAQRVGWKPHADLVEALIGAIFLSNF-

--------RPDLLLLREKQRRMHVNPFAPQEELRDLVVGKRQRRDRLMGLPSGLKNHLEE

DDWAERGGKMGIEKRETETGVIETVE----------ETESGVIEKKD-------DKESGV

IEK-------------EEEMDKAVNEEKGETQTGVIEKKKAAFLPLRHKADFFF-CASVA

-------------------------SAAFCD------------------SFCLTN-----

------------------------------------FLPPLLDRFWWAKWGAAEEGGLAL

PEDKWSSLCV---PGGFMSTEEVE--------------KMWGYTF--KNKRLVT---VAR

SHRS-LRTENL-------------------------------------------------

------GRTAT-------------------Y-----------------------------

-------------------------------------ER--LEFLGDAVLEPLMRYFLF-

----VH--FYELD-----------------------------------------------

------------------------------------------------------------

------------------------EKYLDPIRQALVSNRFLACMISRRLKLTDTAGKSMT

APRLVLH------------------STPAQTEKLRTWWQKYDPEGPDSRFVDELQFVRPE

VQAW--------------------------------------------------------

-------------------LEAGKSLGEAEELVRSGKFVGDEGEGEEGEGDEDGEEGDET

EEGQGDDSARSAEAAAAREVEYDAMGRKMTAHRRKTQEKRKLAGVRDQADGFPRTMAD--

--------------------MYESLIAATFIDT-----------GFNFEK--VWDVISP-

------DFERLL------------------------------------------------

-

>Hypothetical_protein_Paramecium_tetraurelia

EPKDSLSSLVDQMLSSISNNQEILNNKVNLLEEDDTIVDTIEEF---EFRDYQIELYEKG

IGKNSIIYLETGLGKT----LVIIMLM-----------WDRLFKYPDKKIVFLA----NT

VQLVEQQAQQIKQKLP-----------------RVAELISDDDSMIVKAKEIGSKLNVLH

GSKC--TDIW-NQIMWQIILE----------E----------------------------

------------------------------------------------------------

--SKILVMTTQIFLNILRKGLVKISNFSFIAMDECHN-----AIQDHPYNYILKEFYLKT

KFQSDNQF--LPQIVGATASPVMNSKSVSN---NQLLQELLQLSANMDSQYLHI--DAQN

L-----------------------------------------------------------

----KK---HIKEAQIVPIYYKMLFNDPDIQKVIDLKAS-LTNCE-----------KNEN

KALD-----------V--MRRNSYLTEFFQIKQTLENFK-KKYSYDLQTKILIAQIEKQV

LINGFQMY---------------------LELGQ----YLFVLLVEYV------------

------------------------------------------------------------

--IIKLNDFLTIKRPPNQAA---------------------CLDILKSISTILKDAKLR-

------------------------------------------------------------

---YSNVQQSSTPKVQVLFEL----IRKAYSQG---------------------------

------------------------------------------------------------

------------------------DDNSRILIFVKQRLTAF-----FLNRLIEEYLAS--

---------ERINIVSNFIIGHCSSMVRKQNNSIMINVI------------------ESN

KEDQEELIKKLYAELQNDQSKFSIEQLKDQVNRLNIFAYRISSTVQNDIINKFR------

QGKIKILISTS--------VAEEGIDIPMCNYVIGFNPISSSKAYV-------QMKGRAR

KENSQFMIFMV-------------------------------------------------

----------------------------DKL-----------------------------

---------------------------------------QQAQVQSHQQVQNNIKQIIEE

LTAPQRQQMLNKAMQTLQSKTYENTYFDSFEIEGSGA---LINTNWSCQLIQQL------

------CQKFNQK------D--ADKTQP----------------KYAIYQLHGNAQTNQQ

-----FIAFLLLPIS--MKSFIFYG-----KLAPSSKDAKA------------SAAFEAS

VQL------YQKGYIDDNLQI-CFDSD----------LGQHIGA-----------DQDDS

QI------MMTREQWQIACKYS--QRLVQSQSTMG--GKVNS------------QKRYYT

N----LFK------TLFQTKDQEGQF------------EDF-------------------

--LLY----------ECLFQNEKILL-----------AFPKQ-----MLPQI--------

SKSENTLQALGIQFQSM--------------QKISKSQY---------------------

EG--HYK-KYVETIIKE-------------------------------------------

---------------------------------ENLQQWRDDIYAI---INQKCQIRIKS

ESN--------SN------LLLILKGSVKSKFTLSQIINQFAL-----------------

-----------------------------------------------------R------

--------------------------------------FKVDL-----FQDKIKEQLIQ-

------------K---IQIIPQNIK----PHINE--------------------------

VLNSTSKYIKYLTS-------------LQYC-----------------------------

--------------YQNELDSL---QLNFI------HYSVSDVVLNFD-VKD--------

----------------------RLDEILF------------------K--------SLDL

S------------------SH---------------------------------------

------------KYNELLLGHQYYK--------FLIAVVLYANYF---------------

---------------------------END-------INITRAKYKTFTRSTYVRNCLLE

SYLFLYLHKNDL------------------------------------------------

------------------------------------------------------------

---NDITTQFIGLEKFQDNFDMMMEQ----------------------------------

------------------------------------------------------------

------------------------------------------------------------

------------------------------------------------------------

------------------------------------------------------------

------------------------------------------------------------

----------LILDDFSVAKRK-SQRNTIKNKIHISNQEFFQFIQNSLFLIQQNDQ----

----------------------------------DLVNGLNWL-RTFRIMTSMKIH----

VNSSQCLQEFL--------------PGFV-------------------------------

------------------------------------------------------------

------------------------------------------------------------

----------------------------PLSEQTRKIKRVFSSLEYKIKYQFKH-F----

-------------------------ELLFQA------------------MTDISF-----

---------------------------------------------KFVINNKLKFKY-YE

QFQKKEEFQG-----------MIQ--------------EQQWIEF--KSQFECM---NET

DQNN--------------------------------------------------------

------------------------------Y-----------------------------

-------------------------------------ENSLLAMLGKALWNYLTIKILN-

----EN--NLDCF-----------------------------------------------

------------------------------------------------------------

--------------------------AIKIVGKILSKTPFLAYLAIQL-----------G

LQQYLNS----------------------IYSTILLNLASEVEKCKDSSLAFKIGKIQK-

------------------------------------------------------------

-------------------SIHTQCLETAFFALVGAIYL-DSNCSSNIVMQWATLILDNV

QVQQFYSPDFIKR-QPKYQFYQWYQSNIGNPFNFNLIKIPHQDYRRVSQGDKLFIPYD--

--------------------MLEAQIVNIKK-------------DKYYEI--SQLHIYA-

------KSKEKAWEK----------------LYEMIDY----------------------

-

>Dicerlike_protein_Chlamydomonas_reinhardtii

EAAMVERAAEVEGAAEVEGAAAGRAEAEAGPEAGPETRGPEAEL---EARRAAFGVDGQQ

EGNGGGSRGGGGTSDVAMRDTHTNTLDP---ERGAT--SAREVNVEAEVPVPV-----PV

PAAAPRAARLARGGAG--------------------------------------------

GGGG--GGCYDRRRLRPRLLG---------------------------------------

------------------------------------------------------------

--LTASPLHPLLLQECMLGARLVAPPPGSLREWAAHC--------SRPKAQILWVGDDKP

LPPPPALPPAAAAAIAAATPPMPPPAAPQPVQAAGAEEAMETAEDGRERGGVEATHDPEP

LATSDDDLSFIPVPPLPRAFSFSSTSTTDTADSATDMDTATAADAAATAATSTDAATAAA

AADGGGGGSYSCPLRAAIGYC-RRLLLLAERVMSQRERL-LRLHALGEVDLKRLGLEGSE

LLAA-----------GGRHGGADMGGNALRDLRLIRSQIMDSTDLGPPPGPRDAERLRAD

WAELQLALDSCGLPKPGDSSSNGSGGSSSKCTGPDAGATAAAAGAAAV------------

--------------------------------------------------------APAA

TATAAAQRWARRAGVGERVAA----AVLAAA----------MEGCLALAAPPAEEA----

-----------------------------------AAIAAGFGDALLRRGGCRPAGRRVL

ARWAERLLRSVPPALLQFHVL----GLLPPAPHEAAAVAAAHDSWRRRGTPLVAHRVVWL

LRQLHRRALEAAAATATAATVT--------------------------------------

------------------------AGAATAAPAAEAAATAAAPV--VLTAQQRRQQRAAA

ARAAATARSAAVLLRTGVFVGTGGAAVASAPAGGKASGQK--------------------

--------------------------------------------RQDEVRTAFA------

SGRLNVLLSTS--------IGAEGLDFSSCALVAALDLPRHVTPFL-------QSGGRAR

APGSEHWLLVR-------------------------------------------------

------------------------------------------------------------

---------------------------------------TPEELELAQRLMAAEEALQAK

AAHVSAYNLRELGFRIQTPAGNPAAVAAGG----AGTGQDPNPDPDDMDADQAA------

------AARSPSD-----------TPEP----------------LTATAALEA------A

TASGPVAAAAAAAAAAAAAAVVGPGA----GTAAGVDVDVE------------LACLAAV

ALL------WRRGDLDDHLCPRHSRKALTQAAHQAAFGEFQHSI-----------ASAVL

PHPLPRLLRPPGWAPTPGAAAA--AGPLALPAPAG--TADAGAPATAADGATACDGCGVD

A----SGD------LGSSSSGKGGGGVDDASRYSEQGPAPR-------------------

--HLYCFVMEPLARFPGGGGGGGFGSGFGSGFSFGSEPNPGSGGHASTVASS--------

SGEGAAAAAAETGLLQWP-------------YTASAAAAGSNAAEPAAAAADNGGGGGGD

DPMALWRVRRIAGELAAAGPPLAPGAGGGAVQQGGSGSSGSSSTAGKGSSSS--------

--------------------SSGSGGGGAALHVLSSTAMAAAGACWVP-TARNAAAAAAE

LQLQQRQQKQQQQQ-----AGQAQQQQQQQQQSDTMEQDAPPSGGGGGGGGAAAA-----

------------------------------------------------------------

--------------------------------------TAAASAAAATATATATATVLS-

------------WGVLLHGRLPAGLPAVELFVPD--------RKKGELTERETRARVRLA

YLGELPSPPTQPPA-------------EDAVAQGSLAAASA-------------------

ASAT----------AGADGAGNGGGAAAAA------AELVAAVGSAAHSLLR--------

----------------------FPLPVAL-------------------------------

------------------------------------------------------------

------------ARAKALKAAASGA--------VPASSAATDTAADPA------------

---------------------------AAP-------AAEWVGFQRV--AAALEAAAAAA

ANGPAAGGASGGGGGGVEAALKEAVALAAAAGNGGADDAGGMEDASEAQAAEVAEAAEAG

LQAHAQGGTAAAAGGAAV--------------------------------AAVAEAGRQQ

QGRGGGRGRKGWVGYWWLPVPLRPPP----------------------------------

-------------------------------------------------PPPPPAEVTEA

VAAGPAGAGAAAGTAMDVDVDLDLGAAATAAATASDGPCRGRTGDQSSAAAA--------

-----LSYIDWRVVGGLAAPPAPLPADAATAVPLNAAAAATTTASQNAALAAVNSVAGAA

QPPSAVAGAAGAAVAGAGGAAVAGAPAPPCCYVWRQEVVRHGLFISLDSFRMCTFLEPQA

PSAAATATAVAGDATVVPA-----------------------------------------

-----AGAQGRLQQLLQP-----QPQLLQPPPGQPLLVELYSYARCRSHWLSSRLQ----

----------------------------------LQPGQQQQAPRRVAVAAATAATAAGT

AGAATAGAKQVDTVAPAAPGTTI-PTAATAATAQASAEAAAVTAVAGAAPAAPLSTPPES

GSGGGGGATEALMDTYDDGAGAADLADVTGNRGGVYGDGGLGGGGSSGGGGDGSSSSLSG

RKRARQPPGQGLYSTAPSPPRSPPRL----------RPHLEQEQQQQ-------QQQEQQ

QQQQHRHNGNSSNNNGLSASPANDTGSAADGGGWAPVPPAVAPVATPAATATTTKTATAT

-------------------------T-----------------------AAAAAT-----

-----------------------------TTSTTKRLPREVVAPLPPDTGPGRLARFPLD

AGRWRALQGLPPLLWRVEGLVAAA--------------EALERVL--RPLGFAP---EKA

TGGRSDSGGGAEPAAATTTATAAAAGTAAPGGGGNNSGDGGGGGRDEAMADANRDVPSAP

PPPPPLPPPPPLPPPPPLPPHLT-------H-------------------QPPPPPPPPL

PPPPPLPPPLLPPPLPPLVLAAAALTAPAAGDACLNNDR--LEFLGDSVLKHIAVLFTF-

---LRDRHDPTCH-----------------------------------------------

------------------------------------------------------------

------------------------EGVLSFKRDALIANEVAMDVATDF-----------C

VRAGLLP------------------PDAQLVIREGLHAEASTQRAAAAAAAAAAAATAAA

AATAAAASGGKDAAAAAAGGSAAAAG----------------------------------

-------------------AAAGAGPAAAAAAVAAAAKS-TSTRTSVPDVTLAFKSLQPL

TFRESWLRRGGAI-EAADDDATADPAAGATAGR--------SGGRAAASGGGAASSPW--

-----------------------RADTWGGGGG-----------GSSRSVPASPSPYTH-

------VTPQKQQPRPAVAAPGPYVSYTAFVAPYTPPALAAPAAAAA-------------

-

>Dicer_Giardia_assemblage

----------MHALGHCCTVVTTRGPSHWLLLLDTHLG---------TLPGFKVSAGRGL

PAAE--VYFEAG----------------------------------------------PR

VSLSRTDATIVAVYQS--------------------------------------------

------------------------------------------------------------

------------------------------------------------------------

------------------------------------------I-----------------

----------LFQLLGPT-----------------FPASWTEIGATMPHNEYTFP-----

------------------------------------------------------------

-----R---FISNPPQF---------------------------------------ATLA

FLPL--------------------------------------------------------

-------------------------------LSP--------------------------

------------------------------------------------------------

------------------------------------------------------------

------------------------------------------------------------

TSPLDLRALMVTAQLMCDAKR----LSDEYTDYSTL------------------------

------------------------------------------------------------

------------------------SASLHGRMVATPEIS---------------------

-------------WSLYVVLGID-------------------------------------

--------------------------------------------STQTSLSYFT------

----------------------------------RANESITYMRYY-------ATAHNIH

LRAADLPLVA--------------------------------------------------

------------------------------------------------------------

------------------------------------------------------------

----------------------------------------------AVRLDDLK------

------DHQIPA-------PGSWDDLAP----------------KLRFL-----------

------------------------------PPELCLLLPDE------------F-DLIRV

QAL------QFLPEIAKHICD---------------------------------------

----------------------------IQNTICALDKSFPDCGRIGG------ERYFAI

T----AGL----------RLDQGRG-------------RGL-------------------

--AGW--------------------------------RTPFGPFGVSHTDVF--------

QRLELLGDAVLG-------------------FIVTARLL---------------------

CLFP----DASVGTLVE--------------------------------LKM--------

----------------------------------ELVRNEALNYLVQT------------

-----------------------------LGLPQLAEFSNNLV-----------------

------------------------------------------------------------

--------------------------------------AKSKT-----------------

------------WADMYEEIVGSIF-----------------------------------

---------TGPNG----------------------------------------------

--------------IYGCEEFL--------------AKTLMSPEHSKT------------

------------------------------------------------------------

------------------------------------------------------------

-----------------------------------VGSAC--------------------

---------------------------PDA-------VTKASKRVCM---------GEAG

AHEFRSLVDYA-------------------------------------------------

------------------------------------------------------------

------------------------------------------------------------

------------------------------------------------------------

------------------------------------------------------------

------------------------------------------------------------

------------------------------------------------------------

------------------------------------------------------------

-----------------------CEQGISVFCSSRVSTMFLERLRDI-------------

----------------------------------PAEDMLDWY-RLGIQFSHRSG-----

LSGPGGVVSVIDIMTHLARGLWLGSPGFYVE-----------------------------

------------------------------------------------------------

------------------------------------------------------QQTDKN

ESA------------------------CPPTIPVLYIYHRSVQCPVLYGSLTETPTGPVA

-------------------------SK----------------------VLALYE-----

----------------------------------------------------KILAYESS

GGSKHIAAQT------------VS--------------RSLAVPIPSGTIPFLI---RLL

QIAL---TPHV-------------------------------------------------

------------------------------Y-----------------------------

-------------------------------------QK--LELLGDAFLKCSLALHLH-

----AL--HPTLT-----------------------------------------------

------------------------------------------------------------

------------------------EGALTRMRQSAETNSVLGRLTKRF------------

-------------------------PSVVSEVIIESHPKIQPDS----------------

------------------------------------------------------------

-----------------------KVYGDTFEAILAAILL-ACGEEAAGAF----------

------------------------------------------------------------

--------------------------------------------VREHVL--PQVVADA-

------------------------------------------------------------

-

>putative_helicase_Neospora_caninum

AYFGWQAGARQTRERGQGEI--LRDCDAVLPVPEETYSDRGTQGKL-QAWSYQYRVYRHA

VLENAIVTLPTGAGKT----LIASMATHT---------WLQNAFAADKIVVFLA----PT

AFLTLQQQRALSAVLSLFCDPVGAGAPWTGRLVVRLRGILETERLQRDEDCEDETAYFRH

TLQA----DWSHPKYWSAFYSALSEYRAALLDLHQLPAAPPADFDLRRFNLGRLLAPFRF

APSQRAALLREGPGVEHTSERG--------------------RVEGDAPGDSEVDERLSL

RWPRVFVMTPDKFRNLASRGLLRIDRVGLLIFDEAHRVGRLLQGNSSAYATILGVFVH--

----LCRAEKKPRILGLTASPIPDRKLCFFDLDNELKRAMRTLETLFQARIVKAETEKAP

A-----------------------------------------------------------

----GASGRRQEEGDNEEEATRPREDGGAERNADGYRRLRATETEAQRDDASTCTLRTVR

YLRAPAPFYCDRELRVLHRLLSRRSWETGRRAEGEGEQTAGKSAEDASDASDAGFETEQS

DGERRREA---------------------RYLGENE--AREERKKRHI------------

------------------------------------------------------------

--LEETDAKQKNDTEREEKKE------EKNE----------TESEEKEQREEMDLE----

----------------------------------------------------EGQEKSEG

RASAARSRGREDEAWQHFFERETRGRHTYFSFLLQDVLDICSAFFYVEVTRLEGGREERG

KSEDQPETANGETSPGTRERGPGGSC-------------------------------ASS

SPFPQPS-----------------SSLSSSLPSLSSSLPSLSSSLPSLSSSLPSLSSSLP

SLPSSLP--SRSPWSSSSPPPSSSSSPPPSSSLSWDSSQSGPPRSDLLSDVVHEEDVRLN

VEVMRLLWRSVETP------------------------------VRQSAKRKWE------

NFRRHMLHIQRDLGFWCVWRLKAKVDEEFAAWQRRLACGQGGASNLGPGDDSGEGDTRGE

TDGGETDGGEG-------------------------------------------------

----------------------------RGR-----------------------------

----------------------------------------GDKIEKRTVFQGTDTGEGKR

KAKEQRAQDRPRSSSRASLPLWIFFPFLPPPPCHGRCFRSSSAPSFSVSSFGRA------

-GAKEAVAALPVDCLLRRLFFGYFHFFS----------------KLLSAALSNSD-----

-----LKLTMRLCRSRDLAHLGKAF-----DALPSRGLEAE------------ATRALVL

QRVAWSLFSHKLVQLDRSLRE-AFGEQAQTQTREGLRGETCEIR-----------KEQRI

QLGRDAQNAAESTDCEAARKSNSGANLRAPPHSSPPSSSSSSSFSSSSSSSFSSSSSSSF

S----SFFSFPPSPVPCSPSSPGLALGDVPKCGLVFCRRRV-------------------

--DAW----------LLCEFLKARRL-FFVDWICSSSVASQDAAWLFGVGKASRDRNRCL

DRLRATHVVWEKLKASKHKLHD---------RTQRSEQT---------------------

SVPQQLSSSSSFSSSSSSSFSSSFSSSSSSSSCSPDGDGGRAVSRLMRRLGWGAPLQLLV

TTSVLEEGIDLPACNLVVQTDVPSSLVRYVQAKGRARKKPAEFIVLCP-------DSGPD

PSWMDASLPPPSFSS----LASLQSPPPRPIPSLSPLSPLSPLSSRSSCSSLLAWLEENG

ERSRGAVWASGASGAACMHGVSG-----------------SFSELAWLQVCEAKAAELAC

DVDAPETPGRSQPLRRLRRSFRLEELLLTRTGTLVHSAFAPAL-----LREIVDACTVPD

ARGAGKTQMLDVRGNALRDTDSPNSR---LRETPGDGADVRTDKQALSFASLEGRRVSRL

SVGKATPRGRAPPL-------------R-------VLRTEDGKWALDVPSLQGFRASPLP

AFSLELSPSASAALPATAAFAVLALQALRRLVAEGVVDTFLRCRDADSVQVDRNRGDKER

EKRESWGGVGAVDLLWRELPDCLMPPRSWRGWRTEETSESENAGKPGDEPRIGERERRGV

LKVKDGRQGTAQPNGRAARTE---------------------------------------

------------TKRQTDTASQPGKPRKHSRPPPPPAGVTRLFIHRVWCTPLTAREAALE

RSWVP-PSQKKKKERLLRRLKRARGLLPSPLYASGGFCGLWRLFADAEASL--RTAEVAL

EDDLRCRKTAAKTKGETETRTGKGLDNGLWWFGNRRQRESCFVRRERERSGETGVKGKDT

QQRPVAEGEEEEEAEEEEEEEEEEEEEEEEEERKLKSQEEAENRQRRGILQLIRSRIDAV

AEGRGPDSVLQPLCLLLSRPIASPMA----------------------------------

--------------------------------------------------FHAVHPEAEA

TGPAARRRGSTEPRFGSRGKEEGDEDAWNSQDAEEEEEGEGEGEGEGEEDEGEEDEIPGL

MRVEVEKPEKTMFIDLPTAYVEQVLLRFTHIHLCRLLQLFNFSPSGTCNPLILAVLQFLS

ALPSHNSFSSCSSFSSSSSPSFSSFSSPSFSSFSSPSFSSSSSSSFSSSSSSSSSSSSSS

SSSSSSSSSSSSSSSSSTSSSTSSSSSSSSSSSSSSSSSSSSSSSSPSFYPSFSSSSSSS

SSSTSSSSSSASSSAFSSSS---CLRGEGEKKSRLVSLPLRLFASSR-LLPSHAPSLFSP

PPSFSSLLSLAVEDAKEKSSLYCPSLSSPSSLSASSEAREEQL-KTRVAEACTSLAQAGV

VNLQRVLFPFFLLAPLSPADAKSVDLDFI-------------------------------

---------------------------HRVASLRVFLRSGQSRRRGLLEAWERPPSLLQL

HDRRSFRGAVERLHCLPSLSAVARLF----------GETRGKAGQEDRVSLESKGGRWGK

GENATRETEETASPAVDRGLLTRRLPQPGSKSPRRKERTNKNEKEGEEGEEEGEMRGERT

---------------------LSKDERFFLMLQTYNGDSLYRISDTAQAGSRQEK-----

---------------------------NLMRFFRRRSENALSVKDALREEGMEIFYHLRS

RGLEPVALRMTHQFGRCFRFYRLHTIHRHLTPHNVMINSDAGFTLGTLCRVYLKQFKQQL

EEPHPDRERGP-------------------------------------------------

-------RGNCFLVQAVKLRQEISWMPAILFELEVQAKHCDLVRRLKRDLQPFLFAPGAR

KVRLPLEVDFPLVSKSLTAPQSRLAGAVDAAGVEWHSQR--LELLGDAVLKFIVSTYLFF

HVHGDA--PPPCEQTHTDEETKEEKEAKEEKDAKEEKDEEKEKDEEKEKDAEEFGEKEER

DGAKLEGGRRDAFSASFAACLESGEGEAPGWRRGPDRTRERARAKRRLDVREGRRRSRKD

EDCGGSPLSVLTQERADAFFGGVGEGTLSRLRQHYIANDYLRFAMRRF-----------R

LQAHLVAFPFVGHKKSLADLREQPVSEKMQADVVEALIGAIYLANVENVSLFPFSQSEKL

RGEKVGGEKKAQTEERERRDSQRVEKEAAERTGAQRQAVRQRRRKRREGREAARQQ----

-------------------EKDTRFVGSSSGCVAAAAFF-DAYLLLPSVVDEAVCRLEEE

NQRTQQHRRKAEE-QKGESGKEEDRV----QEG--------LAV-RQEGDSPSEATRDEE

KEVLERRKARLAEFVEK---AARAGECFCTSEE-----------GSEKGEKEINKDAWK-

------LLPDRLTKQ-----------TTDLRALARLPHMSQTFLLLWTATETWRRSHTYL

A

>Ribonuclease_type_III_Dicer_Toxoplasma_gondii_ME49

AYCWWQAGARESGNRGHRVLKTCD---AVLPLEEETYSDRDTRGQL-QAWPYQYRVYRRA

TLENTVVALPTGTGKT----LVAAMVVHT---------WLANVFVADKLVIFLA----PT

TQLARQQHRAISAVLSLLRDPAGAEASRGRLAVRLPGVLEKERSTRGRVASRDDDDGERA

YFRSVLRVDWSHPKYWAAFYSALSEYKAALLDLQRLPAAPHAAFDLRRFHTGRLLAPYRF

VDSQRAALLRESFAEPSNEEANAQAASLEDSEGERSGNREGEREEDENEQDVSEVEHQAV

CWPRVFVMTPDKFRNLACRGLLRIDRIGLLIFDEAHT-VGRPLRSLGAYATILRVFLH--

----LCQEEAKPRILGLTASPVPDGKLGFFDLESELKRAMRSLETIFQARIVKA--DTES

A-----------------------------------------------------------

----PGIRNWSQGQPDAQRQGEQQRGVMGVRLTAAGGREIREAVE-----------EESE

RSAA--------------NEARGNARRGSGETRQDHLGGDKRLRTMETTEGDEETFTGCA

LKTVRYQLAPSPFYYDHELLILDRLTTPRVEAGRQQWRVAAWQPPPGT------------

-------------------------------------------------LRGSREPTGGV

SAVSNGDREQRKDRLPRREGTERGDPPTRST----------STKPTGTKERETEEE----

-----------------------------ELDEKKAKEKEAKAKNGQVNCDGLEGKRSEG

REDAVRWNGSEVAAFRHFVEREARGRHTNFSLLLQEVLDICSTFFYLEVTRLKEGREANE

ERHHPHETTPAEEARQETMHRI--------------------------------------

------------------------LKTDCGSPCASHPSPSS-----SSFPSCSPPLSSSP

AAPPLAPSSSCSSASAAFSTSALCSSAAPSRVPASLRSSFLAEVRGAADARLNVRVMRRL

WQHLAPL-------------------------------------TKQRAQRNWE------

NFRRHLLHVQSELGFWSVWRLKEKVEADLEAWQARLGAATEERSRA-------QTRRSRR

DCEGAECEESE-------------------------------------------------

----------------------------KED-----------------------------

-----------------------------------ETDNSGRGKESQKRVRFAGENQRTK

AGRSEEGNRTRGDRDRIKSTLAEIPLWISFPF--------RRPLSSDLDLQHGSSLSSSS

LSSLDPVGGQPVERLLRRLFFGFFFLFS----------------KALSTACSHSS-----

-----LQLTMQLCRSRDLAHVCLADVAALEGGVSGAERTGS------------LPQVLAC

SLF-----SDKLLRLDQVLRD-SFGRAQAQAQTQRREGSVADDKRGSGPDKRAGRPGDEE

ERRTEDGGEATRSGLRIKASSK--GDRECSPSPKK--SGVRTPVPSFSSSPSCVSPSALS

SAPSSMSLSSRFPAASRSPASPDVAFGGVPKCGLVFCRRRV-------------------

--DAW----------LVCEFLTARRL-VFVDWICSSSLASQGASWLFGIGKASQGRSSCL

DRLRAMHTVWGKLEKKTQKLNAQKHRPS---TTPQSHSP---------------------

SLASSLSSSASSSLSSSLSSSASSSASSSASSSASWGDGGKGVWRLMRRLGWGAPLQLLV

STSVLEEGIDVPACNLVVQMDMPSSLVRFVQAKGRARKKPAEFVVLCP------DSGPCG

ASWPDASLPFSSSFPSSSSFFPFSSSLQSQTPDCTVSSPSLLLSLQSVSSLAVSLSSLSA

PFSVSLRSRTGEAPTTPAFAAQGFSAAGGAACYLQAVSPSVNFELARLQLCEAKVAALAA

DVEATERFGLLGPFRCMRKVCSLDDLLLTHTGAMVHSSFAPAL-----LREIVDACTAG-

------------KTDERTERGADERGEEGRWAEDTEDEGREERERGNAELRTGREKALGT

CCSNAPSSSSLSSSGMRRVSDLCETRRQRTPSALRLLSTQDGKWILSVPSLQGFRSSPLP

PFSIEFSPTGSAPTRASAAAGVLAVRVLRRLVQEGVVDSFLRCRDRDS-VEKDLRDEKER

RRRENCGGVGAVDLLWRELPACLLPPRSWRNARRASGDRHGDSEETLH--------TSAK

TREEAARAEEAVSGSNEERRRSRDHKEGTIEGDAGAQEVEENAEEGRKEEEGDQLKRLPS

GTGKKDETSTSNAKSETHREKTRTP--------APPPGVTRLFLHRVWCTPLTAREAALE

RLWEPNEKQRRRRRRVLKKLAESRGFLPSPLYESGGFCGLWRIFADAEAPLQAADLALDG

ASRRRQEKAKEKEQIEPDAGDGEGDEDGLWWTGNRRQREACFEKREREGGDGKHDQCKED

EKCRAEKEEEEEEEEEEEEEEEEEEEEEEEEKKWSWQEEEKNRQRREILELIRSRIDAVV

EGRRGPDSVLQPLCLLLAHPIPATVA----------------------------------

----------------------------------FHALHPEASEKAALHRGRRPRTGRTS

FSLRASKAEKGSNAWTEQEADEEGEEGDEEGEEEEGEEEGEEEGEEDGEEEGDEEDVPGL

MRVEVEKPAKTMFIDLPTEYVEKVLLRFTHIHLFRLLQLSNFSPNGTCNSLLLTVLQFLS

ALPFRRFPFRTVPSSSSLSSPASASASASCLGGDGEPRSRFVSLPLPA------------

------------------------------------------------------------

-----CASASRLLPSHALSLFALPPSLFSLLSLLSLAAEEAEESTES-TLHSQSPP----

------------------SSPRSSSPRSSSLSSSSPSTSAEQL-KRQMVEACSSLAKAGV

VDLQRVMFPFFLLAPLSPLDAKSVDLDFI-------------------------------

---------------------------HRLASLRLFLRSGQRRSSGFLEAGTCPPSLMLQ

HDRRLLRGAVESLHCLPSLSAVARLFCPGNSARKLDYGGGRSLQKAE-------RRSARK

EQN---------EGDAAEENGAFNWPRSLLGTLPSSDRESESAKDTKEKRTPGSEKGERG

EADGERREEDGEKGATKEDGLFSKEEQFFRRLQSYKGSIFSRGDDAANAWTTEEQ-----

---------------------------KLLTFFQQRNENALSVKEALREEGMEIFYHLRS

RGFEPVALRMTHQFGRSFRFYRLQTIHRNLTPRNVVINSDAGFTLGTLCRVYLSRFKQRL

AEPHPDSQRGPQGNCFLVQGVKRRQGARQLFSRERVVDGDEFAGLHAPQFAHLLPISEPM

FQEISWMPAIL-------------------YQLEVQGKHCDLV-----------------

------QLDFRLVSRALTAPQSRQVGAVDAAGVDWHGQR--LEFLGDAVLKFVVSCYLFF

HVHGGK--PAESRADAERKETEKEETALESGEPGDAEAAEEVEEQGEEPESKETGGEREE

QEEEGEEEEARWKFLFSLLTQEEAG-----------------------------------

---------------RF--FGGVDEGVLSRLRQHYVANDYLRFAMRRF-----------R

LQAYLVNFPFVSHKKSLLDLREQPVSEKAQADVIEALLGAVYLSNADSALFGAQPGKRSG

GKETTGKTEDREATEKRGGTEENEREGERREKEREGRGRDVGENVSKKETEGRVDKRQAV

HRRPREMRHRGLDKETTEEREEQAVVGSSSGCMAAAAFF-DAYLLLPSAVNNAVLLFDEE

VEREKGRRETGERDNKLKEREESEEDRLSHEGV--------ALA-PDGAGEQREVPDRQE

KETLEHWKALLVDCLEEALRRRDRGVSVSDLGE-----------DRHKEENAFPCSAKDI

NRDAWMLLPDRLAKQ-----------TEDLRALARLPHMSETFLLLSRATNRQRTPHNGL

S

>Dicer_Cyclospora_cayetanensis

-MGTWRGDPTGWDETAEGPSAESDNEGLFFGVSERESPEGVEER---SGEEGFMRIEIEP

PEEDMQLKVPNGAALTALLEFTHEMLLR---SLSIQ--NFR-----------------PT

VKYSRTGAALWEALFN--------------------------------------------

GAGGGS------------------------------------------------------

------------------------------------------------------------

--PSELSSASLRLYASNQDAFASLPRLRTPQHQSALH-----------------------

-----------KRIDGATDPVVT------------LSQPVGSMDSNSPSEGPQDQ-DAEG

T-----------------------------------------------------------

----SV---AQLLLKGGVSDC-------------------HRILL-----------PFFA

LAPL----------------------------------------------AFDGRALNVS

YICSLRAL---------------------KCFSISSK-WRAEEASRSL------------

------------------------------------------------------------

--MEVYGVDTLRASVQGLASP------SSAL------------RFFRSALQHLEEK----

-----------------------------------------------------------Q

SSPLSGVDPDITGRQEKKVPT---------------------------------------

------------------------------------------------------------

------------------------EEMDSGSEMAVEDLVFS-----PISPLLRVG-----

---------RCFPWKDFCKAGHTMDS----------------------------------

--------------------------------------------TRQDAPRK--------

TTSKRCLQAPA----------------------------------L-------SGQGKEK

HIGGDRGGYTE-------------------------------------------------

------------------------------------------------------------

------------------------------------------------------------

----------------------------------EGT---------TMKKVREA------

------CVQTEG------------TSTK----------------FFEGQGLHK-------

---------------------------------------RL------------LSAALTC

KAS------RLPGALDSG-------------------GNAWHSQ----------------

--------------------RL---------------EFLGDSVLGFV------VAMYIF

F-------------TNFKWEDEGALS------------RRK-------------------

--SSY----------------------------------------VCNKHLA--------

AKAREMGIAGHLLARP---------------FTPSATLV---------------------

ELRE----QILSSKMLA-------------------------------------------

---------------------------------DAVEALTAAVYLSNKTLVRDTSKNSPE

ARS----------------ASKTSHTETRLGVSGHLG-----------------------

------------------------------------------------------------

------------------------------------------------------------

------------------------------------------------------------

------------------------------------------------------------

SFDG----------LIAAAAFL--------------DRFVLKEASTDT------------

------------------------------------------------------------

------------------------------------------------------------

------------GEEDTLKSHLQMD-----------------------------------

------------------------------------------ESSRL-PPIVNVLTATAQ

VSASICCTPAS-------------------------------------------------

------------------------------------------------------------

------------------------------------------------------------

------------------------------------------------------------

------------------------------------------------------------

------------------------------------------------------------

------------------------------------------------------------

------------------------------------------------------------

-----------------------EPEWAQLDATPLVAG----------------------

----------------------------------VPHAPLRPD-PRVIALLQQLMR----

--VSHDSTATSASLGISAERDWR-------------------------------------

------------------------------------------------------------

------------------------------------------------------NEEAVS

VGK-------------------------------ASHQAISNL-----------------

-------------------------------------------------WLSPAK-----

-------------------------------------------------VSSEVASPTSS

NESSGAAFWR-------------D--------------RDLNLPL--VALSRTA---EAP

AAAR----GGP-------------------------------------------------

--------TAT-------------------Y-----------------------------

-------------------------------------QL--LEFLGDSALGFVVSEWLF-

----SL--FPEVR-----------------------------------------------

------------------------------------------------------------

------------------------EGPLTIIKSRLVSNSFFAAKMMR------------K

LHAAGLSYDSLALGWDSFSEPQSWPCLCVRERVIQRLVALPPEEDFTREAAEVLSRLAHS

PSNAAGDNSSGCSSNT--------------------------------------------

-------------------PKWVKQLGDIYEALASATLL-SSGFSFEALWAAV-------

------------------------------------------------------------

--------------------------------------------SADFEICIPQLESFL-

------LALSRARSS----------------QEEHVDT----------------------

-

>Dicer_Eimeria_tenella

--------------------------MAGVRLVPPYP----------DPERGPL------

-----------GN-----------------------------------------------

------------------------------------------------------------

--------CW--------------------------------------------------

------------------------------------------------------------

------------LLRGLR------------IRQGCRW-----------------------

-----------------------------------LTRA-----------------DPGA

A-----------------------------------------------------------

----AG---RSAAPQE----------------------------------------QQTA

YVPQ-----------C--------------------------------------------

-----------------------------ARLCP----WT--------------------

------------------------------------------------------------

------------------------------------------------------------

------------------------------------------------------------

-EPVFLQLTWLAPAFFQLEVA---------------------------------------

------------------------------------------------------------

------------------------------------------------------------

-------------------AGHF-------------------------------------

------------------------------------------------------------

ELKARLLQQTE-----------------------------------------------LR

SPQGPLRLGLE-------------------------------------------------

----------------------------QQL-----------------------------

------------------------------------------------------------

------------------------------------------------------------

------------------------------------------------------------

-----------------------------------------------------LAAALTC

KAA------RPPGAVDSG-------------------GSWWHWQ----------------

--------------------RL---------------EFLGDAVLGFV------AAAFCF

L-------------SNFGGEAEGPLT------------QQK-------------------

--SSF----------------------------------------VSNCHLH--------

SCARGLGLAGYMLSRP---------------FIPAAALA---------------------

GLRE----QVLSPKMLA-------------------------------------------

---------------------------------DAMEAITAAFYLSNR------------

--------------------------GLLGGPRGPSEAQQGPM-----------------

------------------------------------------------------------

-----------------------------------------------------QAAPLG-

------------------------------------------------------------

---GPPQRSQGPPG----------------------------------------------

SFRG----------LLAAAAFI--------------GQFVLQGGPPAP------------

------------------------------------------------------------

------------------------------------------------------------

------------GKGPQAHTGAGES--------QEPPERG--------------------

-----------------------------------------LKAKQL-PPVVNVLSAAAA

VSAAICCGPPL-------------------------------------------------

------------------------------------------------------------

------------------------------------------------------------

------------------------------------------------------------

------------------------------------------------------------

------------------------------------------------------------

------------------------------------------------------------

------------------------------------------------------------

-----------------------SPQGTSRDPKDTFEQAEEEDFLCTLLLPAGVPH----

-------------------------------------TPLHPD-RRVAVLLQQLLR----

-GPREGSSKPQAAA----------IPASC-------------------------------

------------------------------------------------------------

------------------------------------------------------CSSSGA

GSS-------------------------------ISEQTVSDL-----------------

-------------------------------------------------WLAPAR-----

------------------------------------------------DLLNCCYSQEAL

DCKTAAAFWK-------------E--------------RGLCPRL--VALARSA---EVP

GVPQGSPQGGP-------------------------------------------------

--------QEN-------------------Y-----------------------------

-------------------------------------QL--LEFLGDSALSLLVSEWLF-

----WR--FPEAR-----------------------------------------------

------------------------------------------------------------

------------------------EGPLTMARSILVSNAFFAAKMIRR------------

--------------------------CPAAAGKVLLVWLFKGIQKKQSCMQ---------

------------------------------------------------------------

-------------------QECPWTLSSSRAGKG------PPSSNCVSAWGP--PGMGPV

GR------------GPPPEAAFWRACGSRQKQQ--------AAG-CGLGAPAAAAAAA--

--------------------AAAPRRSMLCSGS-------------------SNCPLKR-

-------TSRGKQQN---------------------------------------------

-

>Dicerrelated_RNase_III_protein_Dcr2p_Tetrahymena_thermophila

--YGFNGIPNSYAFKINDESSDEDDEEIFKDQVSPKINEQGKNTIY-EIRNYQKELFKNS

YSKNSIIYLETGAGKT----MIATMLIN---YFFQK--QKKA--PQKKKIIFLA----NT

VQLVKQQYAEIKNNLQRISEMMENDKEISQILKFQEGINYRRIFEKENHIVKCHGSYKQK

DNLQ--IETF-TKQKWDYLLE----------N----------------------------

------------------------------------------------------------

--YEIFVMIGQVLLNGLRRGYIRIEDIEVIIYDECHH--T--R-QDHPYCLIQYEFYHFH

KRRNEQVQ--LPKIYGLTASPVLDIEVKSDS--NIMKKKISELCTNLDAN-FVKY-NIEE

S-----------------------------------------------------------

----KK---YIKEATICVEEY-GEEKNIQER----ANQF-LQEQI-----------IGEK

WISD-----------IEDKQNEEMRYTEILNFYIKFQQF-KNSYFSNLSPMNKQILLDIE

KIICLYSM---------------------QVDAQ----LGKYSVSLFL------------

------------------------------------------------------------

--KDVNDELQKLGQFQHNNEE------MQEL----------KKLFKAQTEKFIES-----

----------------------------------------------------VDKSEKNI

SKKFMFLYNKLKQTMIHDKEE----FE-CYKVKNKYYEQEFHQHEKITKVQKYIK-----

------------------------------------------------------------

------------------------DNQSMILVFVEEKIIIK-----YLQKLLQIWMDDED

RDKDEDEIIFRY-KKIVCLTGQSNFQDRKKKNLNKNQYQNSEFTQNDNFEFAEEEYRQQQ

MKQESLISSQINKEEDIDSLKEKINDHQKSAQRCVLVNLDFEVSKQSENIEAFR------

KKELQIILSTS--------VTEEGFDIPSCNLVISFNKISNLKSFV-------QIKGRAR

KENSKFIIMAK-------------------------------------------------

----------------------------DKY-----------------------------

--------------------------------------QKQDYETDIESYKDLIQQIKEI

APK------------VEKQEIEIRPNYKYKIVKETGA---ILNTNWSVNLFHTY------

------ANQLLYS------YD-SNIKGP----------------FYYYIEITNVG-----

-----FICQIIVPSQSGLKTFC--S-----NPQAQKEDAKK------------DACFQAV

IIL------YRQYKIFDQYLR-NISQKIQNSQSEKSESLFEDEK-----------DEKLT

KI------LQKYCKSYVDVKQK--CKMYYFQSVMG--ECFKQSFFKSILDIQQ-QKSYTF

Q----LYL------IKFNDQGKKLPS------------SGF-------------------

--IKN----------QDSE----LGI-IHST------QIPI----KDTIKYY--------

LQMHENEVYANMIYQQE--------------IQISQQQY---------------------

DKLMLIN-RFLNSVALNKDIEFYAEYTGEVVNGKGKLFKDHG-KNASKNLQY--------

-------------------------------KFDNSEGFSALLTVIRP-SNNNTNLYEIN

FDHIEILEKYINY------MSGVYKVRNKTKLNEEEKAARFQSRKLKQDDPFADYK----

---------------------VK-----------------PYYDCVNFFDCTKQ------

--------------------------------------LDPNI-----YQDSRKKLVLQ-

------------SIMSGRHLNRFIF----YGCKS--------------------------

YEEIQKEYQQVPTG-------------K------------D-------------------

PFKQ----------IKQLKAQV---ELEEVSFTSNENHMVLQVQLFTK-HLR--------

----------------------SQTNLYL------------------K--------RVGV

L---------------TENFK---------------------------------------

------------GDYK-----------------YSPHQF---TIF---------------

---------------------------PLN-------VSLLMKLNILHKTFLYQIRDTFG

VHHFQQIRLPKFFDKAYQRIKNNN----KTSCSYNLDQ---IEQPHFL--VENYQGVLDV

MSKSEKYNQFCKKYNNHQ------------------------------------------

--LMEGYNQISIRKFDQKYIKHNPIF----------------------------------

------------------------------------------------------------

----------------------------ESYQVYKKNIFELQISE---------------

------------------------------------------------------------

------------------------------------------------------------

------------------------------------------------------------

-----------LMKSLTSLEYAVNNKESSLERYEFLGDTVLKCLSST-QIYFEHPK----

----------------------------------SMEDHLHVH-RTIIIQNRNLAQ----

IAVKKKIFKYILSSKIE---TLS-PIGIK-------------------------------

------------------------------------------------------------

YEIAVDDDEQNQFQKEKEPDEYDKAD----------QRNKNKKGNEE-------DGDAEN

EEI----------EEQQQQNDIPNLLRLEPTYRHIKGKTLADTLESIIGVFFQQ----KK

-------------------------DLNLCQ------------------YLLFSL-----

---------------------------------------------QVLNKPDLTFNHSYD

RSLQQSQLQN----PIYVQLKEIE--------------SILGYTF--QNINLLF---QAF

TLPS-IESLCIQIPSLKNTNLNKLHQINNAIQKKENQGTFEKIDQISKENQNKIYQKDSN

EWENLKNYNLC-------------------N-----------------------------

-------------------------------------QR--LEFLGDAVLDLIIVEYLF-

----NK--FPNSD-----------------------------------------------

------------------------------------------------------------

------------------------PGELTQMKTSLVQNKTLCLINLMK-----------G

FYKFIIG------------------SIQHNKEEIELLLNNVEMFMGDTSLMYDTTYN---

------------------------------------------------------------

-------------------QSLIKTLGDVIESLVGAIFI-DNNLDYEITKKIVMETLFAE

IL---QYFTGQE--HRLKNLQLYIEKVLKKEFG--------KVS-------LIEDETD--

--------------------IKEGKKRFFIQSE-----------DNNKEV--SFEVYAY-

------DQIDAIDQI-----------YIEINQKKRQKK----------------------

-

>Endoribonuclease_Dicerlike_protein_1_Auxenochlorella_protothecoides

-----------------MPPHRAERSAFTVAVQAGAAQI--------KPRAYQVELLEMA

KTNNVIAFLDTGAGKT----FIAVMLLQH--RLTAS--LSTH--QATARAVFLV----PR

VPLVFQQAEVLAAHLP-----------------CAVGRYV--------------------

GEMG--VDLW-DSRRWGREWE----------R----------------------------

------------------------------------------------------------

--HGVLVMTPQILLNLLTHAIIPISRVHTIVFDECHH--T--Q-KKHPYNLIMKEFYHR-

--VTGSTP--RPHILGLTAAPANVRREQASW--R-LSKNIVTLEGNLDAHVVTVL-DRED

V-----------------------------------------------------------

----EA---AAPPPTLRICYYSCGQGHPPEL-----RAL-LRAIS-----------HARL

RLAA-----------AGDLVSQVWTHELEQKMGSAKKSS-GRVYFDPVISGFSTSRFNQM

LSGVQHVT---------------------ETLGM----WCGAQAARSIFKTLITEVAPTY

MAGMGANKKGEGFEYQTFNPSAARLAAMMGRDTVVMGETMADEEGSNSDMSDEEVPTFPR

QEIEQEQTATNLSALEVVVAF------AQALLPTGAPQAAKCEDMICMLRRRVEGGQLIK

DLPVKTMEELLGAVTPYELQAEVDALGVVEEAAGPPASAVPGADLRVTAVLAGGAAHVVP

MTQEPRRLGLVTQKVVVLVEQ----LL-QYRGTATQNETD--------------------

------------------------------------------------------------

------------------------GSCWSGIVFVTRRLACW-----AVHMLLSTL----P

C--------TRAFLRTAAVTGYQ------QRTGEAVGVR---------------------

--------------------------------------------HQEETLRGFR------

QGTLNLLVATA--------VVEEGIDVRRCQLVVRFDLPPTAQSYI-------QSRGRAR

MQNSVLILMVQ-------------------------------------------------

----------------------------TEL-----------------------------

----------------------------------------PEELEMINHMIKFEADLRQE

VLSNIHK------MKARRRDGDEELRYMVAS---TGA---RVSAGNVLRLLHTY------

------ISKLPTD------RQAYSILRP----------------TYRTEALLD-------

---GMYLTHAFLPSNCPLRETV--G-----MPQSTRRKSIA------------SAALNAI

RLL------HESGSLNDNLLP-AA-------------FELAAER-----------RHAS-

-----------------GVQSG--G---FHPPAPS--GEHGEVLLHP----------YRW

L----VVD------DRSRTLAASSSG------------PAK-------------------

--AMR----------D-------LVC-LLLE------PLPE---GLPPCRLP--------

GNDGASNQLAF-LPGPA--------------VRVSGDQL---------------------

RQLKACH-VALAQLMLARGPPRHAAG---------------QRRKAEVAVAV--------

---------------------------------AAEAADGEGGMVPEP-DEIVAGATGGT

APA--------PP------HADIGRAGVAHEAAPTPHDDRPED-----------------

-------------------------------------------------KGAGL------

--------------------------------------FVRAL-----VRRIIDRVIIL-

------------QDGHLPKSRRGKA----SRAAG--------------------------

VSPEALSFLLAPAA-------------PSGDG----SIDWR-------------------

LVAEVCGDAGSTSVLALLRRGV---DLRDRIIWTTYNRKVHAFRGVRH-DLN--------

----------------------LQSTFKT------------------S--------EDR-

----------------GSAPR---------------------------------------

------------GKRRAAGAAPAAVSAAGSPADEAPPTTTRTSYA---------------

---------------------------AYY-------AERWGLPNLD-PG-----QPLIE

ASAWKQGAGGRL------------------------------------------------

------------------------------------------------------------

----DAAAEPEEGEASVALVPELTLR----------------------------------

------------------------------------------------------------

------------------------------------------------------------

------------------------------------------------------------

------------------------------------------------------------

------------------------------------------------------------

-----RPSLALLRTALTLRS---AGEGSDYETLETLGDTFMKYAVCA-ELFFENRT----

----------------------------------YHEGQLTRC-KDDVVSNLSLAQ----

RAQALGLHSALQVLPFSVG-NWT-APPIV-------------------------------

------------------------------------------------------------

------------------------------------------------------------

---------------------------------KVGGKVLADGMEALVGAFVVG-SGEEA

-------------------------TRVFMR------------------VLGLLK-----

-----------------------------------------------ALQPSSKLEPAPG

GDADVAHQEV--------DVDAIQ--------------RVLGYTF--ARPGLVC---EAL

THCSWSGRNGS-------------------------------------------------

----------C-------------------Y-----------------------------

-------------------------------------QR--LEFLGDALLDLVVVQHCF-

----DS--FTDVE-----------------------------------------------

------------------------------------------------------------

------------------------PSDLHDMRSVAVNAERLAFVAVHH-----------G

LQRHIRH------------------ASPKLLSHLTDFVSMWRDEETLQTFSGALPEYKL-

------------------------------------------------------------

-------------------VAWVQERAHVQVSCMDVLLG-ESKGGRNA------------

-----RSSGRLAAQDALDRWRELEPK----------------------------------

--------------------------------------------LREALV--LRAAAKK-

------DGLASMK------------------TPKLVRA----------------------

-

>Dicer_Schizosaccharomyces_pombe

----------------------MDISSFLLPQ---------------LLRKYQQDVYNIA

SKQNTLLVMRTGAGKT----LLAVKLIKQKLEEQIL--IQESNLEHKKISVFLV----NK

VPLVFQQAEYIRSQLP-----------------AKVGMFY--------------------

GELS--IEM--SEQLLTNIIL----------K----------------------------

------------------------------------------------------------

--YNVIVITADLFYLFLARGFLSINDLNLIIFDECHH--A--I-GNDAYARIMNDFYHRA

KAVLSKKHFTLPRIFGMTASPFTGK----KG--N-LYHRLYQWEQLFDSKAHVVS-ENEL

A-----------------------------------------------------------

----D----YFCLPEESYVMYSNKLVVPP------SDSI-IKKCE-----------ETLQ

GC----------------------------------------------------KLISRA

VKTALAET---------------------IDMGL----WFGEQVWLYL------------

------------------------------------------------------------

--VDFVETKRLKK--KALGKQ------LSDD----------EELAIDRLKIFVEDWK---

----------------------------------------------------NNKYSDNG

PRIPVFDSTDVTDKVFKLLEL----LKATYRK----------------------------

------------------------------------------------------------

------------------------SDSVRTVIFVERKATAF-----TLSLFMKTL-----

N--------LPN-IRAHSFIGHG----PSDQGEFSMTFR---------------------

--------------------------------------------RQKDTLHKFK------

TGKYNVLIATA--------VAEEGIDVPSCNLVIRFNICRTVTQYV-------QSRGRAR

AMASKFLIFLN-------------------------------------------------

----------------------------TEE-----------------------------

---------------------------------------LLIHERILHE----EKNLKFA

LSELSNSNIFDSLVCEERERVTDDIVYEVGE---TGA---LLTGLYAVSLLYNF------

------CNTLSRD------V--YTRYYP----------------TFTAQPCLS-------

---GWYCFEVELPKACKVPAAQ--G-----SPAKSIRKAKQ------------NAAFIMC

LDL------IRMGLIDKHLKP-LDFRR--------KIADLETLE-----------EDEL-

-------------K---DEGYI--E---TYERYVP--KSWMKVPEDI-------TRCFVS

L----LYT------D--ANEGDNHIF------------HP--------------------

-----------------------LVF-VQAH------SFPK----IDSFILN--------

STVGPRVKIVLETIEDS--------------FKIDSHLL---------------------

ELLKKST-RYLLQFGLS-------------------------------------------

---------------------------------TSLEQQIPTPYWLAP-LNLS------C

TDY--------RF------LENLIDVDTIQNFFKLPEPVQNVT-----------------

------------------------------------------------------------

------------------------------------------D-----LQ---SDTVLL-

------------V-----------------------------------------------

-----NPQSIYEQY-------------A--------------------------------

-FEG----------FVNSEFMIPAKKKDKA------PSALCKKLPLRL-NYS--------

----------------------LWGNRA--------------------------------

----------------KSIPK---------------------------------------

------------SQ-------QVRS--------FYINDL---YIL---------------

---------------------------PVS-------RHLKNSALLI-PSILYHIENLLV

ASSFIEHFRLDC------------------------------------------------

------------------------------------------------------------

--------------------KIDTAC----------------------------------

------------------------------------------------------------

------------------------------------------------------------

------------------------------------------------------------

------------------------------------------------------------

------------------------------------------------------------

-------------QALTSAE---SQLNFDYDRLEFYGDCFLKLGASI-TVFLKFPD----

----------------------------------TQEYQLHFN-RKKIISNCNLYK----

VAIDCELPKYALSTPLEIR-HWC-PYGFQ-------------------------------

------------------------------------------------------------

--------------------------------------------------------KSTS

DKC------------------------RYAVLQKLSVKRIADMVEASIGACLLD-SGLDS

-------------------------ALKICK------------------SLSVGL-----

-----------------------------------------LDISNWDEWNNYFDLNTYA

DSLRNVQFPY---------SSYIE--------------ETIGYSF--KNKKLLH---LAF

IHPSMMSQQGI-------------------------------------------------

--------YEN-------------------Y-----------------------------

-------------------------------------QQ--LEFLGDAVLDYIIVQYLY-

----KK--YPNAT-----------------------------------------------

------------------------------------------------------------

------------------------SGELTDYKSFYVCNKSLSYIGFVL-----------N

LHKYIQH------------------ESAAMCDAIFEYQELIEAFRETASENPWF------

------WFE---------------------------------------------------

-------------------IDSPKFISDTLEAMICAIFL-DSGFSLQSL-QFVLPLFLNS

LGDATHTKAKGDIEHKVYQLLKDQGC--EDFGT--------K---CVIEEVKSSHKTL--

--------------------LNTELHLTKYYGFSFFRHGNIVAYGKSRKVANAKYIMKQ-

------RLLKLLEDK----------------SNLLLYSCNCKFSK---------------

-

>Dicer2_isoform_A_Drosophila_melanogaster

-------------------------------MEDVEI----------KPRGYQLRLVDHL

TKSNGIVYLPTGSGKT----FVAILVLK---RFSQD--FDKPIESGGKRALFMC----NT

VELARQQAMAVRRCTN-----------------FKVGFYV--------------------

GEQG--VDDW-TRGMWSDEIK----------K----------------------------

------------------------------------------------------------

--NQVLVGTAQVFLDMVTQTYVALSSLSVVIIDECHH--G--T-GHHPFREFMRLFT---

--IANQTK--LPRVVGLTGVLIKGN---EIT--N-VATKLKELEITYRGNIITVS-DTKE

M-----------------------------------------------------------

----ENVMLYATKPTEVMVSF-PHQEQVLTV----TRLI-SAEIE-----------KFYV

SLDL-----------M--NI-------------GVQPIR-RSKSLQCLRDPSKKSFVKQL

FNDFLYQM---------------------KEYGI----YAASIAIISL------------

-------------------------------------------------IVE--------

--FDIKRRQAETLSVKLMHRT------ALTL----------CEKIRHLLVQKLQDM----

----------------------------------------------------TYDDDDDN

VNTEEVIMNFSTPKVQRFLMS----LKVSFADKD--------------------------

------------------------------------------------------------

------------------------PKDICCLVFVERRYTCK-----CIYGLLLNYIQSTP

E--------LRNVLTPQFMVGRN---NISPDFESVLERK---------------------

--------------------------------------------WQKSAIQQFR------

DGNANLMICSS--------VLEEGIDVQACNHVFILDPVKTFNMYV-------QSKGRAR

TTEAKFVLFTA-------------------------------------------------

----------------------------DKE-----------------------------

---------------------------------------REKTIQQIYQYRKAHNDIAEY

LKDRVLEKTEPELYEIKGHFQDDIDPFTNE----NGA---VLLPNNALAILHRY------

------CQTIPTD------A--FGFVIP----------------WFHVLQEDERDRIFGV

SAKGKHVISINMPVNCMLRDTIY-S-----DPMDNVKTAKI------------SAAFKAC

KVL------YSLGELNERFVP-KTLKE-RVASIADVHFEHWNKY-----------GDSV-

-T------ATVNKA---DKSKD--R---TYKTECP--LEFYDALPRVG------EICYAY

E----IFL----------EPQFESCE------------YTE-------------------

--HMY----------LNLQTPRNYAI-LLRN------KLPR----LAEMPLF--------

SNQGKLHVRVANAPLEV--------------IIQNSEQL---------------------

ELLHQFH-GMVFRDILK-------------------------IWHPFFVLD---------

------------------------------------RRSKENSYLVVP-LILGAGEQK-C

FDW--------EL------MTNFRRLPQSHGSNVQQREQQPAP-----------------

------------------------------------------------------------

---------------------------------------RPED-----FE---GKIVTQ-

------------W---YANYD--KP----MLVTK--------------------------

VHRELTPLSYMEKN-------------QQ----------DK-------------------

TYYE----------FTMSKYGNRIGDVVHK------DKFMIEVRDLTE-QLT--------

----------------------F-------------------------------------

----------------YVHNR---------------------------------------

------------GKFNAKSKAKMKV--------ILIPELC--FNF---------------

---------------------------NFP-------GDLWLKLIFL-PSILNRMYFLLH

AEALRKRFNTYL-NLHLLPFNGTDYMPRPLEIDYSLKR---NVDPLGN--VIPTEDIEEP

KSLLEPMPTKSIEASVAN--------------------------------------LEIT

EFENPWQKYMEPVDLSRNLLSTYPVE----------------------------------

------------------------------------------------------------

--------------------------LDYYYHFSVGNVCEMNEMDFEDKEYWAKNQFHMP

TGNIYGNRTPAKTNANVPALMPSKPTVRGKVKPLLILQKTVSKEHITPAEQGE-------

------------------------------------------------------------

------------------------------------------------------------

-----------FLAAITASS---AADVFDMERLEILGDSFLKLSATL-YLASKYSD----

----------------------------------WNEGTLTEV-KSKLVSNRNLLF----

CLIDADIPKTLNTIQFTPRYTWL-PPGIS-------------------------------

-----------------------------------------LPHNVLALWRENPEFAKII

GPHNLRDLALGDEESLV-KGNCSDIN----------YNRFVEGCRAN-------GQSFYA

GAD-----------------FSSEVNF-CVGLVTIPNKVIADTLEALLGVIVKN-YGLQH

-------------------------AFKMLE------------------YFKICR-----

--------------------------------------------ADIDKPLTQLLNLELG

GKKMRANVNTTEIDGFLINHYYLE--------------KNLGYTF--KDRRYLL---QAL

THPS-YPTNRI-------------------------------------------------

--------TGS-------------------Y-----------------------------

-------------------------------------QE--LEFIGDAILDFLISAYIF-

----EN--NTKMN-----------------------------------------------

------------------------------------------------------------

------------------------PGALTDLRSALVNNTTLACICVRH-----------R

LHFFILA------------------ENAKLSEIISKFVNFQESQGHRVTNYVRILLEEAD

VQPTPLDLDDELDMTELPHANKCISQEAEKGVPPKGEFNMSTN-----------------

-------------------VDVPKALGDVLEALIAAVYL-DCR-DLQRTWEVIFNLFEPE

LQ---EFTRKVPI-NHIRQLVEHKHA--KPVFS--------S---PIVEGETVMVSCQ--

--------------------FTCMEKTIKVYGF-----------GSNKDQ--AKLSAAK-

------HALQQLSKC----------------DA---------------------------

-

>Dicer2_isoform_B_Drosophila_melanogaster

-------------------------------MEDVEI----------KPRGYQLRLVDHL

TKSNGIVYLPTGSGKT----FVAILVLK---RFSQD--FDKPIESGGKRALFMC----NT

VELARQQAMAVRRCTN-----------------FKVGFYV--------------------

GEQG--VDDW-TRGMWSDEIK----------K----------------------------

------------------------------------------------------------

--NQVLVGTAQVFLDMVTQTYVALSSLSVVIIDECHH--G--T-GHHPFREFMRLFT---

--IANQTK--LPRVVGLTGVLIKGN---EIT--N-VATKLKELEITYRGNIITVS-DTKE

M-----------------------------------------------------------

----ENVMLYATKPTEVMVSF-PHQEQVLTV----TRLI-SAEIE-----------KFYV

SLDL-----------M--NI-------------GVQPIR-RSKSLQCLRDPSKKSFVKQL

FNDFLYQM---------------------KEYGI----YAASIAIISL------------

-------------------------------------------------IVE--------

--FDIKRRQAETLSVKLMHRT------ALTL----------CEKIRHLLVQKLQDM----

----------------------------------------------------TYDDDDDN

VNTEEVIMNFSTPKVQRFLMS----LKVSFADKD--------------------------

------------------------------------------------------------

------------------------PKDICCLVFVERRYTCK-----CIYGLLLNYIQSTP

E--------LRNVLTPQFMVGRN---NISPDFESVLERK---------------------

--------------------------------------------WQKSAIQQFR------

DGNANLMICSS--------VLEEGIDVQACNHVFILDPVKTFNMYV-------QSKGRAR

TTEAKFVLFTA-------------------------------------------------

----------------------------DKE-----------------------------

---------------------------------------REKTIQQIYQYRKAHNDIAEY

LKDRVLEKTEPELYEIKGHFQDDIDPFTNE----NGA---VLLPNNALAILHRY------

------CQTIPTD------A--FGFVIP----------------WFHVLQEDERDRIFGV

SAKGKHVISINMPVNCMLRDTIY-S-----DPMDNVKTAKI------------SAAFKAC

KVL------YSLGELNERFVP-KTLKE-RVASIADVHFEHWNKY-----------GD-M-

-T------ATVNKA---DKSKD--R---TYKTECP--LEFYDALPRVG------EICYAY

E----IFL----------EPQFESCE------------YTE-------------------

--HMY----------LNLQTPRNYAI-LLRN------KLPR----LAEMPLF--------

SNQGKLHVRVANAPLEV--------------IIQNSEQL---------------------

ELLHQFH-GMVFRDILK-------------------------IWHPFFVLD---------

------------------------------------RRSKENSYLVVP-LILGAGEQK-C

FDW--------EL------MTNFRRLPQSHGSNVQQREQQPAP-----------------

------------------------------------------------------------

---------------------------------------RPED-----FE---GKIVTQ-

------------W---YANYD--KP----MLVTK--------------------------

VHRELTPLSYMEKN-------------QQ----------DK-------------------

TYYE----------FTMSKYGNRIGDVVHK------DKFMIEVRDLTE-QLT--------

----------------------F-------------------------------------

----------------YVHNR---------------------------------------

------------GKFNAKSKAKMKV--------ILIPELC--FNF---------------

---------------------------NFP-------GDLWLKLIFL-PSILNRMYFLLH

AEALRKRFNTYL-NLHLLPFNGTDYMPRPLEIDYSLKR---NVDPLGN--VIPTEDIEEP

KSLLEPMPTKSIEASVAN--------------------------------------LEIT

EFENPWQKYMEPVDLSRNLLSTYPVE----------------------------------

------------------------------------------------------------

--------------------------LDYYYHFSVGNVCEMNEMDFEDKEYWAKNQFHMP

TGNIYGNRTPAKTNANVPALMPSKPTVRGKVKPLLILQKTVSKEHITPAEQGE-------

------------------------------------------------------------

------------------------------------------------------------

-----------FLAAITASS---AADVFDMERLEILGDSFLKLSATL-YLASKYSD----

----------------------------------WNEGTLTEV-KSKLVSNRNLLF----

CLIDADIPKTLNTIQFTPRYTWL-PPGIS-------------------------------

-----------------------------------------LPHNVLALWRENPEFAKII

GPHNLRDLALGDEESLV-KGNCSDIN----------YNRFVEGCRAN-------GQSFYA

GAD-----------------FSSEVNF-CVGLVTIPNKVIADTLEALLGVIVKN-YGLQH

-------------------------AFKMLE------------------YFKICR-----

--------------------------------------------ADIDKPLTQLLNLELG

GKKMRANVNTTEIDGFLINHYYLE--------------KNLGYTF--KDRRYLL---QAL

THPS-YPTNRI-------------------------------------------------

--------TGS-------------------Y-----------------------------

-------------------------------------QE--LEFIGDAILDFLISAYIF-

----EN--NTKMN-----------------------------------------------

------------------------------------------------------------

------------------------PGALTDLRSALVNNTTLACICVRH-----------R

LHFFILA------------------ENAKLSEIISKFVNFQESQGHRVTNYVRILLEEAD

VQPTPLDLDDELDMTELPHANKCISQEAEKGVPPKGEFNMSTN-----------------

-------------------VDVPKALGDVLEALIAAVYL-DCR-DLQRTWEVIFNLFEPE

LQ---EFTRKVPI-NHIRQLVEHKHA--KPVFS--------S---PIVEGETVMVSCQ--

--------------------FTCMEKTIKVYGF-----------GSNKDQ--AKLSAAK-

------HALQQLSKC----------------DA---------------------------

-

>Deathpromoting_deoxyribonuclease_Caenorhabditis_elegans

--------------------------MVRVRADLQCF----------NPRDYQVELLDKA

TKKNTIVQLGTGSGKT----FIAVLLLK---EYGVQ--LFAPLDQGGKRAFFVV----EK

VNLVEQQAIHIEVHTS-----------------FKVGQVH--------------------

GQTS--SGLWDSKEQCDQFMK----------R----------------------------

------------------------------------------------------------

--HHVVVITAQCLLDLIRHAYLKIEDMCVLIFDECHH--A--LGSQHPYRSIMVDYKL--

--LKKDKP--VPRVLGLTASLIKAK--VAPE--K-LMEQLKKLESAMDSVIETAS-DLVS

L-----------------------------------------------------------

----SK---YGAKPYEVVIIC-KDF-EIGCLGIPNFDTV-IEIFD-----------ETVA

FVNT-----------T-----------------TEFHPD-LDL--------DPRRPIKDS

LKTTRAVF---------------------RQLGP----WAAWRTAQVW------------

------------------------------------------------------------

--EKELGKIIKSQVLPDKTLR------FLNM----------AKTSMITIKRLLEPE----

----------------------------------------------------MKKI----

-KSIEALRPYVPQRVIRLFEI----LE-TFNPEF--------QKERMKLEK---------

------------------------------------------------------------

------------------------AEHLSAIIFVDQRYIAY-----SLLLMMRHIKSWEP

K--------FKF-VNPDYVVGAS-GRNLASSDSQGL-HK---------------------

--------------------------------------------RQTEVLRRFH------

RNEINCLIATS--------VLEEGVDVKQCNLVIKFDRPLDMRSYV-------QSKGRAR

RAGSRYVITVE-------------------------------------------------

----------------------------EKD-----------------------------

---------------------------------------TAACDSDLKDFQQIEKILLSR

HRTVNNPIEDDSDRFEEFDVDSQMEPYVVEK---TGA---TLKMSTAIALINRY------

------CSKLPSD------I--FTRLVP----------------HNQIIPIEE-NGVTK-

-----YCAELLLPINSPIKHAIVLK-----NPMPNKKTAQM------------AVALEAC

RQL------HLEGELDDNLLP-KGRESIAKL------LEHIDEE-----------PDEYA

PG------IAAKVG---SSKRK--Q---LYDKKIA--RALNESFVEAD------KECFIY

A----FEL------ERFREAELTLNP------------KRR-------------------

--KFE----------DPFNYEYCFGF-LSAK------EIPK----IPPFPVF--------

LRQGNMKVRLI-VAPKK--------------TTVTAAQL---------------------

QEIQLFH-NYLFTQVLQ--------------------------MCKTGNLEF--------

----------------------------------DGTSNAPLNTLIVP-LNKRKDDMSYT

INM--------KY------VSEVVAN-MENMPRIPKDEVRRQY-----------------

-----------------------------------------------------K------

--------------------------------------FNAED-----YK---DAIVMP-

------------W---YRNLEQPVF----YYVAE--------------------------

ILPEWRPSSKFPDT-------------H-----------FE-------------------

TFNE----------YFIKKYKL---EIYDQ------NQSLLDVDFTST-RLN--------

----------------------LLQPRIQ------------------N--------QPRR

SRTVSNSSTSNIPQASASDSK---------------------------------------

------------ESNTSVPHSSQRQ--------ILVPELM--DIH---------------

---------------------------PIS-------ATLWNVIAAL-PSIFYRVNQLLL

TDELRETILVKAFGKEKTKL-DDNVEWNSLAYATEYEE-----KQTII--VKKIQQLRDL

NQKSIEDQERETRENDKIDDGEELFNIGVWDPEEAVRIGVEISSRDDRMDGEDQDTVGLT

QGLHDGN-----ISDEDDELPFVMHD--YTARLTSNRNGIGAWSGSESIVPSG--WGD--

--------------------------------------------WDGPEPDNSPMPFQIL

GGPGGLNVQALMADVGRVFDPSTA--SSSLSQTVQESTVSPPKQLTKEEEQFKKLQNDL-

-----LKQAKERLEALEMSEDMEKPRRL---------EDTVNLEDYGDDQENQEDENTPT

NFPKTIDEEIEELSIGARKKQEIDDNAAKTDVLERENCEVLPVAINEKSRSFSFEKESKA

INGRLIRQRSEEYVSHIDSDI---------------------------------------

---GLGVSPCLLLTALTTSN---AADGMSLERFETIGDSFLKFATTD-YLYHTLLD----

----------------------------------QHEGKLSFA-RSKEVSNCNLYR----

LGKKLGIPQLIVANKFDAHDSWL-PPCYI-------------------------------

--------PTCDFKAPNT-----DDAEEKDNEIERILDGQVIEEK-------------PE

NKTGWDI-----------GGDVSKST----------TDGIETITFPK-------QARVGN

DDI-------------------SPLPYNLLTQQHISDKSIADAVEALIGVHLLT-LGPNP

-------------------------TLKVMN------------------WMGLKV-----

-----------------------------------------IQKDQKSDVPSPLLRFIDT

PTNPNASLNFLNNLWQQFQFTQLE--------------EKIGYRF--KERAYLV---QAF

THAS-YINNRV-------------------------------------------------

--------TGC-------------------Y-----------------------------

-------------------------------------QR--LEFLGDAVLDYMITRYLF-

----ED--SRQYS-----------------------------------------------

------------------------------------------------------------

------------------------PGVLTDLRSALVNNTIFASLAVKF-----------E

FQKHFIA------------------MCPGLYHMIEKFVKLCSERNFDTNFNAEMYMVTTE

EEIDEGQEED--------------------------------------------------

-------------------IEVPKAMGDIFESVAGAIYL-DSGRNLDTTWQVIFHMMRGT

IE---LCCANPPR-SPIRELMEFEQS--KVRFS--------KME-RILESGKVRVTVE--

--------------------VVNNMR---FTGM-----------GRNYRI--AKATAAK-

------RALKYLHQI----------------EQQRRQSPSLTTV----------------

-

>Endoribonuclease_Dicer_Danio_rerio

----------MAGLQLVTPASSPMGPFFGLPWQQEAIHDNIY-----TPRKYQVELLEAA

LEHNTIVCLNTGSGKT----FIAVLLIK---ELSHQ--IRGE---NGKRTVFLV----NA

ASSVAQQASTVRTHSD-----------------LQVGDYM--------------------

SEDM--T-SW-PEEMWNREMI----------E----------------------------

------------------------------------------------------------

--NQVLVMTCHIFLHVLKNGVLPLSKINLLVFDECHL--A--I-TGHPYREIMKICE---

----GCPS--CPRILGLTASILNGK--CDPC--D-LEEKIQNLEKILQSNAETAT-DLVV

L-----------------------------------------------------------

----DR---YASQPREEVLDC-GQYQDQSGL----SERL-LNELD-----------EALN

FLND-----------C--N--------------LSVHRE-DR---------DPTFISKQV

LNDCRAVL---------------------TVLGP----WCADKAAGIM------------

------------------------------------------------------------

--VRELQKYIKHEQ-EELNRK------FLLF----------TDTILRKIHALCEEH----

----------------------------------------------------F-------

-SPASLDLKFVTPKVIRLLEI----LH-EYKPFE--------RQQFESVEWYNNRNQDNY

VSWSDSE-DDDEDEEAEAKE-------------------------------------KTE

ANFPSPF-----------------TNILCGIIFVERRYTAV-----VLNRLIKEAGKQDP

E--------LAY-ISSNFITGHSIGKNQPRNKQMEVEFR---------------------

--------------------------------------------KQEEVLRKFR------

AHETNLLIATS--------IVEEGVDIPKCNLVVRFDLPTEYRSYV-------QSKGRAR

APVSNYIMLAD-------------------------------------------------

----------------------------SER-----------------------------

---------------------------------------TKTFQEDLKTYKAIEKILRNK

CSKSAECNDFELE--PVTDDDNVLPPYVLRSE--DGG--PRVTMNTAIGHVNRY------

------CARLPSD------P--FTHLAP----------------KCKTVEMNT-GG----

-----YRSTLFLPINSPLRVPVT-G-----PVMNCARLAEK------------AVALLCC

EKL------HKIGELDDHLMP-VGKETVKYEEE----LDLHDEE-----------ETSV-

---------PGRPG---STKRR--Q---CSPKAIP--ECLRGCYPVPE------QPCYLY

V----IGM------VLTTPLPDELNF------------RRR-------------------

--KLY----------PPEDTTRCFGI-LTAK------PIPR----IPHFPVY--------

TRSGEVTISIE-LQKSG--------------FSLSAEQL---------------------

ELITRLH-QYIFSHILR--------------------------LEKPA-LEF--------

----------------------------------KPVE-ADSAYCVLP-LNIVEDSNTLD

LDF--------KF------MEDIEKSEARIGIPNTQYTKQNPF-----------------

-----------------------------------------------------I------

--------------------------------------FKLED-----YQ---DAVIIP-

------------R---YRNFDQPHR----FYVAD--------------------------

VYTDLTPLSKFPSP-------------E-----------YE-------------------

TFAE----------YYKTKYNL---DLSNV------NQPLLDVDHTSS-RLN--------

----------------------LLTPRHL------------------N--------QKGK

A----------LPLSSAEKRK---------------------------------------

------------AKWESL---QNKQ--------ILVPELC--AIH---------------

---------------------------PIP-------ASLWRKAVCL-PSILYRLHCLLT

AEELRSQTAIDA-GVGAQTL-PPDFRYPNLDFGW---------KKSID--SKSFISCPSA

---CMEEDDDHCKLGTSS------------DSN---HTAPESC------------SMEVS

QPPEGAPNTPD-EKLETLTLPVTDLNKDC-----F------------PNLPNGTQADS--

--------------------------------------------DDLPH-----------

--------------------------RSDVCQCSQLGPLERDLST---------------

-----QTTTSVSVRPSPAGEPQPWPSD----------ECTGRSSDLCDPHVKKPTSKH-C

PKSETATSTPAPSETSSE------------------------------------------

----DCRSACAGPAWDSPKTL---------------------------------------

-----GPNPGLILQALTLSN---ASDGFNLERLEMLGDSFLKHAITT-YLFCTYPD----

----------------------------------AHEGRLSYM-RSKKVSNCNLYR----

LGKKKGLPSRMVVSIFDPPVNWL-PPGYVVNQDKSSTDKWDSDENKD---LANGKASDDE

DEDDDDEPEEAEVEPSKEDVNVEDD-LEYYYEHIRFIDSMLIGSGAFGKKISLQP---TD

PGYEWKAPKKAHNSHFSPDGGADEFD----------YSSWDAMCYLD-------PSKAGE

EDDFVVGFWNPSEENCGTDIGKQSISYDLHTEQCIADKSIADCVEALLGCYLTS-CGERA

-------------------------AQLFLC------------------SLGLKVLPPEK

QSSGGSA---------------------------------ELQYGWLKIPPRCMFEH-PD

AERTLNHLIS--------GFLNFE--------------SKINYTF--KNKAYLL---QAF

THAS-YHYNTI-------------------------------------------------

--------TDC-------------------Y-----------------------------

-------------------------------------QR--LEFLGDAILDYLITKHLY-

----ED--PRQHS-----------------------------------------------

------------------------------------------------------------

------------------------PGVLTDLRSALVNNTIFASLAVKY-----------D

YHKYFKA------------------VSPELFHVIDDFVQFQLEKNEMQGMDSELRRSEED

EE----KEED--------------------------------------------------

-------------------IEVPKAMGDIFESLAGAIYM-DSGMSLETVWQVYYPMMRPL

IE---KFSANVPR-SPVRELLEMEPE--TAKFS--------PAE-RTYDG-KVRVTVE--

--------------------VVGKGK---FKGV-----------GRSYRI--AKSAAAR-

------RALRSLKAN----------------QPQVQNN----------------------

-

>Endoribonuclease_Dicer_Xenopus_tropicalis

----------MAGLQLMTPASSPMGPFFGLPWQQEAIHDNIY-----TPRKYQVELLEAA

LDHNTIVCLNSGSGKT----FIAVLLSK---ELSYQ--IRGDFSKNTKRTVFLV----NS

EKQVSQQVSAVRTHTD-----------------LKVGEYS--------------------

DQEK--TQCW-AKERWYLEFE----------T----------------------------

------------------------------------------------------------

--HQVLVMTCHIFLNVLKSGNVSLSNINLLVFDECHL--A--I-QDHPYREIMKICE---

----SCQP--CPRILGLTASILNGK--CDPR--D-LEEKIQKLEEILRSNAETAT-DLVV

L-----------------------------------------------------------

----DR---YASQPCEIVLDC-GPYIDKSGL----YQRL-LNELD-----------EALN

FLID-----------C--N--------------ISTHSK-ER---------DSTLISKQI

LSDCQTVL---------------------LVLGP----WCADKVAGMM------------

------------------------------------------------------------

--VRELQKYIKHEQ-EELHRK------FLLF----------TDTILRKIHALCEEH----

----------------------------------------------------F-------

-SPASLDMKFVTPKVIKLLEI----LR-KYKPYE--------RQQFESVEWYNNRNQDNY

VSWSDSEDDDDEDEEIEEKE-------------------------------------KTE

TSFPSPF-----------------TNILCGIIFVERRYTAV-----VLNRLIKEAGKQDP

E--------LAY-ISSNFITGHGIGKNQPRNKQMEVEFR---------------------

--------------------------------------------KQEEVLRKFR------

AHETNLLIATS--------IVEEGVDIPKCNLVVRFDLPSEYRSYV-------QSKGRAR

APISNYIMLAD-------------------------------------------------

----------------------------SDK-----------------------------

---------------------------------------IKAFEEDLKTYKAIEKILRNK

CSKSIDCGNTESE--PIVDDDEIFPPYVLRQD--DGS--PRVTINTAIGHINRY------

------CARLPSD------P--FTHLAP----------------KCKTREFPD-GL----

-----YRSTLYLPINSPLRAPIV-G-----PPMNCGRLADR------------AVALICC

KKL------HEIGELDDHLMP-VGKETVKYEEE----LDLHDEE-----------ETSV-

---------PGRPG---STKRR--Q---CYPKAIP--ECLRNSYPKPG------QPCYLY

V----IGM------VLTTPLPDELNF------------RRR-------------------

--KLY----------PPEDTTRCFGI-LTAK------PIPQ----IPHFPVY--------

TRSGEVTISIE-LKKSG--------------FTLNLEQL---------------------

ELITRLH-QYIFSHILR--------------------------LEKPA-LEF--------

----------------------------------KPTV-ADCAYCVLP-LNVVNDSGTLD

IDF--------KF------VEDIEKSEARTGIPNTQYSAESPF-----------------

-----------------------------------------------------I------

--------------------------------------FKLED-----YQ---DAVIIP-

------------RQVIYRNFDQPHR----FYVAD--------------------------

VYTDLTPLSKFPSP-------------E-----------YE-------------------

TFAE----------YYKTKYNL---DLTNL------NQPLLDVDHTSS-RLN--------

----------------------LLTPRHL------------------N--------QKGK

A----------LPLSSAEKRK---------------------------------------

------------AKWESL---QNKQ--------ILVPELC--AIH---------------

---------------------------PVP-------ASLWRKAVCL-PSILYRLHCLLT

AEELRAQTAIDA-GVGVKSL-PDDFRYPNLDFGW---------KRSID--SKTFISNQSS

---SSVESESDCRLNKTT------------APD---SAASSAANSVIYMQINDQMSVNCT

PPCQKSLSHLQTVCFSDDYKAINGIS--C-----N-------------GLTNG----D--

--------------------------------------------WEAES-----------

--------------------------A-ACFQKDERITCKQEIP----------------

-----EKSTSFHVQNL-PKENQPIL-K----------ECTLSNS---DGNVSKPTSDE-C

PSTCT------------S------------------------------------------

----DMHYDSGLSNRHSSKTL---------------------------------------

-----GPNPGLILQALTLSN---ASDGFNLERLEMLGDSFLKHAITT-YLFCTYPD----

----------------------------------AHEGRLSYM-RSKKVSNCNLYR----

LGKKKGSPSRMVVSIFDPPVNWL-PPGYIVNQDK-NSDKWESNETSGEDVMVNGKIDEDF

--DDEED-EDLMWRNPKEETDFDDDFLEYDQEHIKFIDSMLMGSGAFVKKIPLSSFAPPD

QNYEWRAPKKPPLESSQFPCDFDDFD----------YSSWDAMCYLD-------PSKAVE

EDDFVVGFWNPSEENCGADAGKQSISYDLHTEQCIADKSIADCVEALLGCYLTS-CGERA

-------------------------AQLFLC------------------SLGLKVLPEVR

KLVTNTNVISASSSYQNSTRD---NCTLTARTNTDLSSCKGIDYGYLKIPPRCMFEH-PD

AEKTLDHLIS--------GFENFE--------------KKINYPF--KNKAYLL---QAF

THAS-YHYNTI-------------------------------------------------

--------TDC-------------------Y-----------------------------

-------------------------------------QR--LEFLGDAILDYLITKHLY-

----ED--PRQHS-----------------------------------------------

------------------------------------------------------------

------------------------PGVLTDLRSALVNNTIFASLAVKY-----------D

YHKYFKA------------------ISPELFHVIDDFVQFQLEKNEMQGMDSELRRSEED

EE----KEED--------------------------------------------------

-------------------IEVPKAMGDIFESLAGAIYM-DSGMSLETVWHVYYPMMQPL

IE---KFSANVPR-SPVRELLEMEPE--TAKFS--------PAE-RTYDG-KVRVTVE--

--------------------VVGKGK---FKGV-----------GRSYRI--AKSAAAR-

------RALRSLKAN----------------QSQVPNS----------------------

-

>Endoribonuclease_Dicer_Gallus_gallus

MKSPALQSLSMAGLQLMTPASSPMGPFFGLPWQQEAIHDNIY-----TPRKYQVELLEAA

LDHNTIVCLNTGSGKT----FIAVLLTK---ELSYQ--IRGDFNKNGKRTVFLV----NS

ANQVAQQVSAVRTHSD-----------------LKVGEYS--------------------

SLEV--TESW-TKEKWSQEFS----------K----------------------------

------------------------------------------------------------

--HQVLVMTCHVALTVLRNEYLSLSNINLLVFDECHL--A--I-QDHPYREIMKICE---

----DYPS--CPRILGLTASILNGK--CDPA--E-LEEKIKKLEKILKSNAETAT-DLVV

L-----------------------------------------------------------

----DR---YTSQPCEIVVDC-GPYTDKSGL----YGRL-LRELD-----------EALH

FLND-----------C--N--------------ISVHSK-ER---------DSTLISKQI

LSDCRAVL---------------------VVLGP----WCADKVAGMM------------

------------------------------------------------------------

--VRELQKYIKHEQ-EELHRK------FLLF----------TDTFLRKIHALCEEH----

----------------------------------------------------F-------

-SPASLDLKFVTPKVIKLLEI----LR-KYKPYE--------RQQFESVEWYNNRNQDNY

VSWSDSE-DDDEDEEIEEKE-------------------------------------KPE

TNFPSPF-----------------TNILCGIIFVERRYTAV-----VLNRLIKEAGKQDP

E--------LAY-ISSNFITGHGIGKNQPRNKQMEVEFR---------------------

--------------------------------------------KQEEVLRKFR------

AHETNLLIATS--------IVEEGVDIPKCNLVVRFDLPTEYRSYV-------QSKGRAR

APISNYIMLAD-------------------------------------------------

----------------------------TDK-----------------------------

---------------------------------------IKSFEEDLKTYKAIEKILRNK

CSKSVDTSETETE--PIVDDDDVFPPYVLRTD--ENS--PRVTINTAIGHINRY------

------CARLPSD------P--FTHLAP----------------KCKTRELPD-HT----

-----FYSTLYLPINSPLRASIV-G-----PPMSCARLAER------------VVALICC

EKL------HKIGELDDHLMP-VGKETVKYEEE----LDLHDEE-----------ETSV-

---------PGRPG---STKRR--Q---CYPKAIP--ECLRDSYPKPD------QPCYLY

V----IGM------VLTTPLPDELNF------------RRR-------------------

--KLY----------PPEDTTRCFGI-LTAK------PIPQ----IPHFPVY--------

TRSGEVTISIE-LKKSG--------------FTLSLQML---------------------

ELITRLH-QYIFSHILR--------------------------LEKPA-LEF--------

----------------------------------KPTE-ADSAYCVLP-LNIVDDSSTLD

IDF--------KF------MEDIEKSEARTGIPSTQYTKEMPF-----------------

-----------------------------------------------------I------

--------------------------------------FKLED-----YQ---DAVIIP-

------------R---YRNFDQPHR----FYVAD--------------------------

VYTDLTPLSKFPSP-------------E-----------YE-------------------

TFAE----------YYKTKYNL---DLTNL------NQPLLDVDHTSS-RLN--------

----------------------LLTPRHL------------------N--------QKGK

A----------LPLSSAEKRK---------------------------------------

------------AKWESL---QNKQ--------ILVPELC--AIH---------------

---------------------------PIP-------ASLWRKAVCL-PSILYRLHCLLT

AEELRAQTATDA-GVGVKSL-PADFRYPNLDFGW---------KKSID--SKSFISIPSS

---SLVENENYCKHSTIV------------VPE---NAAHQGANRTSSAEKHDQMSVSYR

TLLDESPSKLQ-IDVSAELAAINGVS--Y-----N------------KNLANG----N--

--------------------------------------------CDLVN-----------

--------------------------R-DFCQGNQLNYCRQEIPV---------------

-----QPTTSYPIQNLYSSENQPKPSN----------ECTLLSNKYLDGNANRSTSDG-C

PKMTVTTSTSTALNLSKD------------------------------------------

----KVDSEKNTSSGYSSKTL---------------------------------------

-----GPNPGLILQALTLSN---ASDGFNLERLEMLGDSFLKHAITT-YLFCTYPD----

----------------------------------AHEGRLSYM-RSKKVSNCNLYR----

LGKKKGLPSRMVVSIFDPPVNWL-PPGYIVNQDKSNTDKWEKEETTKENLLANGKLD--Y

DDDDEED-EDLMWRLPKEETDFEDDFLEYDQEHIKFIDSMLMGSGAFVKKISLSHFSTTD

SNYEWKAPKKSSLGNVPFSSDFDDFD----------YSSWDAMCYLD-------PSKAVE

EDDFVVGFWNPSEENCGVDAGKQSISYDLHTEQCIADKSIADCVEALLGCYLTS-CGERA

-------------------------AQLFLC------------------SLGLKVLPVIK

KTDWESTLCATGENCNSEQKNLSPNSVSACIVNSEPSLYKDLEYGCLKIPPRCMFDH-PD

AEKTLNHLIS--------GFENFE--------------KKINYSF--KNKAYLL---QAF

THAS-YHYNTI-------------------------------------------------

--------TDC-------------------Y-----------------------------

-------------------------------------QR--LEFLGDAILDYLITKHLY-

----ED--PRQHS-----------------------------------------------

------------------------------------------------------------

------------------------PGVLTDLRSALVNNTIFASLAVKY-----------D

YHKYFKA------------------VSPELFHVIDDFVQFQMEKNEMQGMDSELRRSEED

EE----KEED--------------------------------------------------

-------------------IEVPKAMGDIFESLAGAIYM-DSGMSLEMVWQVYYPMMRPL

IE---KFSANVPR-SPVRELLEMEPE--TAKFS--------PAE-RTYDG-KVRVTVE--

--------------------VVGKGK---FKGV-----------GRSYRI--AKSAAAR-

------RALRSLKAN----------------QPQVPNS----------------------

-

>Endoribonuclease_Dicer_Mus_musculus

----------MAGLQLMTPASSPMGPFFGLPWQQEAIHDNIY-----TPRKYQVELLEAA

LDHNTIVCLNTGSGKT----FIAVLLTK---ELAHQ--IRGDLNPHAKRTVFLV----NS

ANQVAQQVSAVRTHSD-----------------LKVGEYS--------------------

DLEV--NASW-TKERWSQEFT----------K----------------------------

------------------------------------------------------------

--HQVLIMTCYVALTVLKNGYLSLSDINLLVFDECHL--A--I-LDHPYREIMKLCE---

----SCPS--CPRILGLTASILNGK--CDPE--E-LEEKIQKLERILRSDAETAT-DLVV

L-----------------------------------------------------------

----DR---YTSQPCEIVVDC-GPFTDRSGL----YERL-LMELE-----------AALD

FIND-----------C--N--------------VAVHSK-ER---------DSTLISKQI

LSDCRAVL---------------------VVLGP----WCADKVAGMM------------

------------------------------------------------------------

--VRELQKYIKHEQ-EELHRK------FLLF----------TDTLLRKIHALCEEY----

----------------------------------------------------F-------

-SPASLDLKYVTPKVMKLLEI----LR-KYKPYE--------RQQFESVEWYNNRNQDNY

VSWSDSE-DDDDDEEIEEKE-------------------------------------KPE

TNFPSPF-----------------TNILCGIIFVERRYTAV-----VLNRLIKEAGKQDP

E--------LAY-ISSNFITGHGIGKNQPRSKQMEAEFR---------------------

--------------------------------------------KQEEVLRKFR------

AHETNLLIATS--------VVEEGVDIPKCNLVVRFDLPTEYRSYV-------QSKGRAR

APISNYVMLAD-------------------------------------------------

----------------------------TDK-----------------------------

---------------------------------------IKSFEEDLKTYKAIEKILRNK

CSKSADGAEADVH--AGVDDEDAFPPYVLRPD--DGG--PRVTINTAIGHINRY------

------CARLPSD------P--FTHLAP----------------KCRTRELPD-GT----

-----FYSTLYLPINSPLRASIV-G-----PPMDSVRLAER------------VVALICC

EKL------HKIGELDEHLMP-VGKETVKYEEE----LDLHDEE-----------ETSV-

---------PGRPG---STKRR--Q---CYPKAIP--ECLRESYPKPD------QPCYLY

V----IGM------VLTTPLPDELNF------------RRR-------------------

--KLY----------PPEDTTRCFGI-LTAK------PIPQ----IPHFPVY--------

TRSGEVTISIE-LKKSG--------------FTLSQQML---------------------

ELITRLH-QYIFSHILR--------------------------LEKPA-LEF--------

----------------------------------KPTG-AESAYCVLP-LNVVNDSGTLD

IDF--------KF------MEDIEKSEARIGIPSTKYSKETPF-----------------

-----------------------------------------------------V------

--------------------------------------FKLED-----YQ---DAVIIP-

------------R---YRNFDQPHR----FYVAD--------------------------

VYTDLTPLSKFPSP-------------E-----------YE-------------------

TFAE----------YYKTKYNL---DLTNL------NQPLLDVDHTSS-RLN--------

----------------------LLTPRHL------------------N--------QKGK

A----------LPLSSAEKRK---------------------------------------

------------AKWESL---QNKQ--------ILVPELC--AIH---------------

---------------------------PIP-------ASLWRKAVCL-PSILYRLHCLLT

AEELRAQTASDA-GVGVRSL-PVDFRYPNLDFGW---------KKSID--SKSFISSCNS

---SLAESDNYCKHSTTV------------VPE---HAAHQGATRPS-LENHDQMSVNCK

RLPAESPAKLQ-SEVSTDLTAINGLS--Y-----N------------KNLANG----S--

--------------------------------------------YDLVN-----------

--------------------------R-DFCQGNQLNYFKQEIPV---------------

-----QPTTSYPIQNLYNYENQPKPSN----------ECPLLSNTYLDGNANTSTSDG-S

PAVSTMPAMMNAVKALKD------------------------------------------

----RMDSEQSPSVGYSSRTL---------------------------------------

-----GPNPGLILQALTLSN---ASDGFNLERLEMLGDSFLKHAITT-YLFCTYPD----

----------------------------------AHEGRLSYM-RSKKVSNCNLYR----

LGKKKGLPSRMVVSIFDPPVNWL-PPGYVVNQDKSNSEKWEKDEMTKDCLLANGKLGEAC

----EEE-EDLTWRAPKEEAEDEDDFLEYDQEHIQFIDSMLMGSGAFVRKISLSPFSASD

SAYEWKMPKKASLGSMPFASGLEDFD----------YSSWDAMCYLD-------PSKAVE

EDDFVVGFWNPSEENCGVDTGKQSISYDLHTEQCIADKSIADCVEALLGCYLTS-CGERA

-------------------------AQLFLC------------------SLGLKVLPVIK

RTSREKALDPAQENGSSQQKSLSGSC--ASPVGPRSSAGKDLEYGCLKIPPRCMFDH-PD

AEKTLNHLIS--------GFETFE--------------KKINYRF--KNKAYLL---QAF

THAS-YHYNTI-------------------------------------------------

--------TDC-------------------Y-----------------------------

-------------------------------------QR--LEFLGDAILDYLITKHLY-

----ED--PRQHS-----------------------------------------------

------------------------------------------------------------

------------------------PGVLTDLRSALVNNTIFASLAVKY-----------D

YHKYFKA------------------VSPELFHVIDDFVKFQLEKNEMQGMDSELRRSEED

EE----KEED--------------------------------------------------

-------------------IEVPKAMGDIFESLAGAIYM-DSGMSLEVVWQVYYPMMQPL

IE---KFSANVPR-SPVRELLEMEPE--TAKFS--------PAE-RTYDG-KVRVTVE--

--------------------VVGKGK---FKGV-----------GRSYRI--AKSAAAR-

------RALRSLKAN----------------QPQVPNS----------------------

-

>Endoribonuclease_Dicer_Bos_taurus

MKSPALQPLSMAGLQLMTPASSPMGPFFGLPWQQEAIHDNIY-----TPRKYQVELLEAA

LDHNTIVCLNTGSGKT----FIAVLLTK---ELSYQ--IRGDFNRNGKRTVFLV----NS

ANQVAQQVSAVRTHSD-----------------LKVGEYS--------------------

NLEV--SASW-TKEKWNQEFT----------K----------------------------

------------------------------------------------------------

--HQVLIMTCYVALNVLKNGYLSLSDINLLVFDECHL--A--I-LDHPYREIMKLCE---

----NCPS--CPRILGLTASILNGK--CDPE--E-LEEKIQKLEKILKSNAETAT-DLVV

L-----------------------------------------------------------

----DR---YTSQPCEIVVDC-GPFTDRSGL----YERL-LMELE-----------EALN

FIND-----------C--N--------------ISVHSK-ER---------DSTLISKQI

LSDCRAVL---------------------VVLGP----WCADKVAGMM------------

------------------------------------------------------------

--VRELQKHIKHEQ-EELHRK------FLLF----------TDTFLRKIHALCEEH----

----------------------------------------------------F-------

-SPASLDLKFVTPKVIKLLEI----LR-KYKPYE--------RQQFESVEWYNNRNQDNY

VSWSDSE-DDEEDEEIEEKE-------------------------------------KPE

TNFPSPF-----------------TNILCGIIFVERRYTAV-----VLNRLIKEAGKQDP

E--------LAY-ISSNFITGHGIGKNQPRNKQMEAEFR---------------------

--------------------------------------------KQEEVLRKFR------

AHETNLLIATS--------IVEEGVDIPKCNLVVRFDLPTEYRSYV-------QSKGRAR

APISNYVMLAD-------------------------------------------------

----------------------------TDK-----------------------------

---------------------------------------IKSFEEDLKTYKAIEKILRNK

CSKSVDTGEADTE--PVVDDDDVFPPYVLRPE--D-G--PRVTINTAIGHVNRY------

------CARLPSD------P--FTHLAP----------------KCRTRELPD-GT----

-----FYSTLYLPINSPLRASIV-G-----PPMSCIRLAER------------VVALICC

EKL------HKIGELDDHLMP-VGKETVKYEEE----LDLHDEE-----------ETSV-

---------PGRPG---STKRR--Q---CYPKAIP--ECLRESYPRPG------QPCYLY

V----IGM------VLTTPLPDELNF------------RRR-------------------

--KLY----------PPEDTTRCFGI-LTAK------PIPQ----IPHFPVY--------

TRSGEVTISIE-LKKSG--------------FTLSLQML---------------------

ELITRLH-QYIFSHILR--------------------------LEKPA-LEF--------

----------------------------------KPTD-ADSAYCVLP-LNVVNDSSTLD

IDF--------KF------MEDIEKSEARIGIPSTKYSKETPF-----------------

-----------------------------------------------------V------

--------------------------------------FKLED-----YQ---DAVIIP-

------------R---YRNFDQPHR----FYVAD--------------------------

VYTDLTPLSKFPSP-------------E-----------YE-------------------

TFAE----------YYKTKYNL---DLTNL------NQPLLDVDHTSS-RLN--------

----------------------LLTPRHL------------------N--------QKGK

A----------LPLSSAEKRK---------------------------------------

------------AKWESL---QNKQ--------ILVPELC--AIH---------------

---------------------------PIP-------ASLWRKAVCL-PSILYRLHCLLT

AEELRAQTASDA-GVGVRSL-PVDFRYPNLDFGW---------KKSID--SKSFISIANS

---SSAENENYCKHSTIV------------VPE---NAAHQGANRTSPLENHDQMSVNCR

TLFSESPGKLQ-IEVSTDLTAINGLS--Y-----N------------KSLANG----S--

--------------------------------------------YDLAN-----------

--------------------------R-DFCQGNHLNYYKQEIPV---------------

-----QPTTSYPIQNLYNYENQPKPSD----------ECTLLSNKYLDGNANTSTSDG-S

PVTAAVPGTTETGEAPPD------------------------------------------

----RTASEQSPSPGYSSRTL---------------------------------------

-----GPNPGLILQALTLSN---ASDGFNLERLEMLGDSFLKHAITTVSLSALILD----

----------------------------------AHEGRLSYM-RSKKVSNCNLYR----

LGKKKGLPSRMVVSIFDPPVNGL-PPGYVVNQDKSNTEKWEKDEMTKDCMLANGKLDDDF

EEEEEEE-EDLMWRAHKEDADDEDDFLEYDQEHIKFIDNMLMGSGAFVKKISLSPFSATD

SAYEWKMPKKSSLGSLPFSSDFEDFD----------YSSWDAMCYLD-------PSKAVE

EDDFVVGFWNPSEENCGVDTGKQSISYDLHTEQCIADKSIADCVEALLGCYLTS-CGERA

-------------------------AQLFLC------------------SLGLKVLPVIK

RTDREKAMCPTRENFTSQQKNLSGSRAAASGAGYRASVLKDLEYGCLKIPPRCMFDH-PE

ADRTLRHLIS--------GFENFE--------------KKINYRF--KNKAYLL---QAF

THAS-YHYNTI-------------------------------------------------

--------TDC-------------------Y-----------------------------

-------------------------------------QR--LEFLGDAILDYLITKHLY-

----ED--PRQHS-----------------------------------------------

------------------------------------------------------------

------------------------PGVLTDLRSALVNNTIFASLAVKY-----------D

YHKYFKA------------------VSPELFHVIDDFVQFQLEKNEMQGMDSELRRSEED

EE----KEED--------------------------------------------------

-------------------IEVPKAMGDIFESLAGAIYM-DSGMSLETVWQVYYPMMRPL

IE---KFSANVPR-SPVRELLEMEPE--ITKFS--------PAE-RTYDG-KVRVTVE--

--------------------VVGKGK---FKGV-----------GRSYRI--AKSAAAR-

------RALRSLKAN----------------QPQVPNS----------------------

-

>Endoribonuclease_Dicer_isoform_2_Homo_sapiens

MKSPALQPLSMAGLQLMTPASSPMGPFFGLPWQQEAIHDNIY-----TPRKYQVELLEAA

LDHNTIVCLNTGSGKT----FIAVLLTK---ELSYQ--IRGDFSRNGKRTVFLV----NS

ANQVAQQVSAVRTHSD-----------------LKVGEYS--------------------

NLEV--NASW-TKERWNQEFT----------K----------------------------

------------------------------------------------------------

--HQVLIMTCYVALNVLKNGYLSLSDINLLVFDECHL--A--I-LDHPYREIMKLCE---

----NCPS--CPRILGLTASILNGK--CDPE--E-LEEKIQKLEKILKSNAETAT-DLVV

L-----------------------------------------------------------

----DR---YTSQPCEIVVDC-GPFTDRSGL----YERL-LMELE-----------EALN

FIND-----------C--N--------------ISVHSK-ER---------DSTLISKQI

LSDCRAVL---------------------VVLGP----WCADKVAGMM------------

------------------------------------------------------------

--VRELQKYIKHEQ-EELHRK------FLLF----------TDTFLRKIHALCEEH----

----------------------------------------------------F-------

-SPASLDLKFVTPKVIKLLEI----LR-KYKPYE--------RQQFESVEWYNNRNQDNY

VSWSDSE-DDDEDEEIEEKE-------------------------------------KPE

TNFPSPF-----------------TNILCGIIFVERRYTAV-----VLNRLIKEAGKQDP

E--------LAY-ISSNFITGHGIGKNQPRNKQMEAEFR---------------------

--------------------------------------------KQEEVLRKFR------

AHETNLLIATS--------IVEEGVDIPKCNLVVRFDLPTEYRSYV-------QSKGRAR

APISNYIMLAD-------------------------------------------------

----------------------------TDK-----------------------------

---------------------------------------IKSFEEDLKTYKAIEKILRNK

CSKSVDTGETDID--PVMDDDDVFPPYVLRPD--DGG--PRVTINTAIGHINRY------

------CARLPSD------P--FTHLAP----------------KCRTRELPD-GT----

-----FYSTLYLPINSPLRASIV-G-----PPMSCVRLAER------------VVALICC

EKL------HKIGELDDHLMP-VGKETVKYEEE----LDLHDEE-----------ETSV-

---------PGRPG---STKRR--Q---CYPKAIP--ECLRDSYPRPD------QPCYLY

V----IGM------VLTTPLPDELNF------------RRR-------------------

--KLY----------PPEDTTRCFGI-LTAK------PIPQ----IPHFPVY--------

TRSGEVTISIE-LKKSG--------------FMLSLQML---------------------

ELITRLH-QYIFSHILR--------------------------LEKPA-LEF--------

----------------------------------KPTD-ADSAYCVLP-LNVVNDSSTLD

IDF--------KF------MEDIEKSEARIGIPSTKYTKETPF-----------------

-----------------------------------------------------V------

--------------------------------------FKLED-----YQ---DAVIIP-

------------R---YRNFDQPHR----FYVAD--------------------------

VYTDLTPLSKFPSP-------------E-----------YE-------------------

TFAE----------YYKTKYNL---DLTNL------NQPLLDVDHTSS-RLN--------

----------------------LLTPRHL------------------N--------QKGK

A----------LPLSSAEKRK---------------------------------------

------------AKWESL---QNKQ--------ILVPELC--AIH---------------

---------------------------PIP-------ASLWRKAVCL-PSILYRLHCLLT

AEELRAQTASDA-GVGVRSL-PADFRYPNLDFGW---------KKSID--SKSFISISNS

---SSAENDNYCKHSTIV-------------PE---NAAHQGANRTSSLENHDQMSVNCR

TLLSESPGKLH-VEVSADLTAINGLS--Y-----N------------QNLANG----S--

--------------------------------------------YDLAN-----------

--------------------------R-DFCQGNQLNYYKQEIPV---------------

-----QPTTSYSIQNLYSYENQPQPSD----------ECTLLSNKYLDGNANKSTSDG-S

PVMAVMPGTTDTIQVLKG------------------------------------------

----RMDSEQSPSIGYSSRTL---------------------------------------

-----GPNPGLILQALTLSN---ASDGFNLERLEMLGDSFLKHAITT-YLFCTYPD----

----------------------------------AHEGRLSYM-RSKKVSNCNLYR----

LGKKKGLPSRMVVSIFDPPVNWL-PPGYVVNQDKSNTDKWEKDEMTKDCMLANGKLDEDY

EEEDEEE-ESLMWRAPKEEADYEDDFLEYDQEHIRFIDNMLMGSGAFVKKISLSPFSTTD

SAYEWKMPKKSSLGSMPFSSDFEDFD----------YSSWDAMCYLD-------PSKAVE

EDDFVVGFWNPSEENCGVDTGKQSISYDLHTEQCIADKSIADCVEALLGCYLTS-CGERA

-------------------------AQLFLC------------------SLGLKVLPVIK

RTDREKALCPTRENFNSQQKNLSVSCAAASVASSRSSVLKDSEYGCLKIPPRCMFDH-PD

ADKTLNHLIS--------GFENFE--------------KKINYRF--KNKAYLL---QAF

THAS-YHYNTI-------------------------------------------------

--------TDC-------------------Y-----------------------------

-------------------------------------QR--LEFLGDAILDYLITKHLY-

----ED--PRQHS-----------------------------------------------

------------------------------------------------------------

------------------------PGVLTDLRSALVNNTIFASLAVKY-----------D

YHKYFKA------------------VSPELFHVIDDFVQFQLEKNEMQGM----------

------------------------------------------------------------

----------------------------------------DSEKSFLQMYPV------PL

CENCLKWNQKLPNLARLRELTT--------------------------------------

--------------------------------------------GRSESL-------WK-

------------------------------------------------------------

-

>Dicer1_Drosophila_melanogaster

---------------------------MAFHWCDNNLHTTVF-----TPRDFQVELLATA

YERNTIICLGHRSSKE----FIALKLLQ---ELSRR--ARR----HGRVSVYLSCEVGTS

TEPCSI-YTMLTHLTD-----------------LRV------------------------

-WQE--QPDM-QIP-FDHCWT----------D----------------------------

------------------------------------------------------------

--YHVSILRPEGFLYLLETRELLLSSVELIVLEDCHD--S--A-VYQRIRPLFENHIM--

--PAPPAD--RPRILGLAGPLHSAG--CELQ--Q-LSAMLATLEQSVLCQIETAS-DIVT

V-----------------------------------------------------------

----LR---YCSRPHEYIVQC-APF-EMDEL----SLVL-ADVLN-----------THKS

FLLD-----------H--RY-----------DPYEIYGT-DQFMDELKDIPDPKVDPLNV

INSLLVVL---------------------HEMGP----WCTQRAAHHF------------

------------------------------------------------------------

--YQCNEKLKVKTP-HERHYL------LYCL----------VSTALIQLYSLCEHA----

----------------------------------------------------FHRHLGSG

SDSRQTIERYSSPKVRRLLQT----LR-CFKPEEVHTQADGLRRMRHQVDQADFNRLSHT

LESKCRMVDQMDQPPTETRALVATLEQILHTTEDRQTNRSAARVTPTPTPAHAKPKPSSG

ANTAQPRTRRRVYTRRHHRDHNDGSDTLCALIYCNQNHTAR-----VLFELLAEISRRDP

D--------LKF-LRCQYTTDRVA-DPTTEPKEAELEHR---------------------

--------------------------------------------RQEEVLKRFR------

MHDCNVLIGTS--------VLEEGIDVPKCNLVVRWDPPTTYRSYV-------QCKGRAR

AAPAYHVILVAPSYKSPTVGSVQLTDRSHRYICATGDTTEADSDSDDSAMPNSSGSDPYT

FGTARGTVKILNPEVFSKQPPTACDIKLQEIQDELPAAAQLDTSNSSDEAVSMSNTSPSE

SSTEQKSRRFQCELSSLTEPEDTSDTTAEIDTAHSLASTTKDLVHQMAQYREIEQMLLSK

CANTEPPEQEQSE--AER-FSACLAAYRPKPHLLTGAS---VDLGSAIALVNKY------

------CARLPSD------T--FTKLTA----------------LWRCTRNER-AGVTL-

-----FQYTLRLPINSPLKHDIV-G-----LPMPTQTLARR------------LAALQAC

VEL------HRIGELDDQLQP-IGKEGFRALEPDWECFELEPED-----------EQIVQ

LS------DEPRPG---TTKRR--Q---YYYKRIA--SEFCDCRPVAG------APCYLY

F----IQL------TLQCPIPEEQNT------------RGR-------------------

--KIY----------PPEDAQQGFGI-LTTK------RIPK----LSAFSIF--------

TRSGEVKVSLE-LAKER--------------VILTSEQI---------------------

VCINGFL-NYTFTNVLR--------------------------LQKFL-MLF--------

----------------------------------DPDS-TENCVFIVP-T-VKAPAGGKH

IDW--------QF------LELIQAN-GNTMPRAVPDEERQAQ-----------------

-----------------------------------------------------P------

--------------------------------------FDPQR-----FQ---DAVVMP-

------------W---YRNQDQPQY----FYVAE--------------------------

ICPHLSPLSCFPGD-------------N-----------YR-------------------

TFKH----------YYLVKYGL---TIQNT------SQPLLDVDHTSA-RLN--------

----------------------FLTPRYV------------------N--------RKGV

A----------LPTSSEETKR---------------------------------------

------------AKRENL---EQKQ--------ILVPELC--TVH---------------

---------------------------PFP-------ASLWRTAVCL-PCILYRINGLLL

ADDIRKQVSADL-GLGRQQIEDEDFEWPMLDFGWSLSE---VLKKSRE--SKQKESLKDD

TINGKDLADVEKKPTSEE------------T-----QLDK-DSKDDKVEKSAIELIIEGE

EKLQEADDFIE-IGTWSNDMADDIAS--F-----NQEDDDEDDAFHLPVLPANVKFCDQQ

TRYGSPTFWDVSNGESGFKGPKSSQNKQGGKGKAKGPAKPTFNYYDSDNSLGSSYDDDDN

AGPLNYMHHNYSSDDDDVADDIDAG-RIAFTSKNEAETIETAQEVEKRQKQLSIIQATNA

NERQYQQTKNLLIGFNFKHEDQKEPATIRYEESIAKLKTEIESGGMLVPHDQQLVLKR-S

DAAEAQVAKVSMMELLKQLLPYVNEDVLAKKLGDR--RELLLSDLVELNADWVARHEQET

YNVMGCGDSFDNYNDHHRLNLDEKQLKLQYERIEIEPPTSTKAITSAILPAGFSFDRQPD

LVGHPGPSPSIILQALTMSN---ANDGINLERLETIGDSFLKYAITT-YLYITYEN----

----------------------------------VHEGKLSHL-RSKQVANLNLYR----

LGRRKRLGEYMIATKFEPHDNWL-PPCYYV------------------------------

---------------PKE------------------LEKALIEAKI--------------

PTHHWKLADLLDIKNLS-SVQICEMV----------REKADALGLEQ-------NGGAQN

GQL------DDSNDSC--NDFSCFIPYNLVSQHSIPDKSIADCVEALIGAYLIE-CGPRG

-------------------------ALLFMA------------------WLGVRVLPITR

QLDGGNQ------------------EQRIPGSTKPNAENVVTVYGAWPTPRSPLLHFAPN

ATEELDQLLS--------GFEEFE--------------ESLGYKF--RDRSYLL---QAM

THAS-YTPNRL-------------------------------------------------

--------TDC-------------------Y-----------------------------

-------------------------------------QR--LEFLGDAVLDYLITRHLY-

----ED--PRQHS-----------------------------------------------

------------------------------------------------------------

------------------------PGALTDLRSALVNNTIFASLAVRH-----------G

FHKFFRH------------------LSPGLNDVIDRFVRIQQENGHCISEEYYL-LSEEE

CD----DAED--------------------------------------------------

-------------------VEVPKALGDVFESIAGAIFL-DSNMSLDVVWHVYSNMMSPE

IE---QFSNSVPK-SPIRELLELEPE--TAKFG--------KPE-KLADGRRVRVTVD--

--------------------VFCKGT---FRGI-----------GRNYRI--AKCTAAK-

------CALRQLKKQ----------------GLIAKKD----------------------

-

>Endoribonuclease_Dicer_1_Apis_mellifera

---------------------------MAFPLNDQIYTKSF------TPREYQVELFYAA

KDKDIIVCLGKNYEQT----FLVIKLIQ---EFATN--NRRLLSQGGKRSLYIL----TD

EDKCTIKASYIQQLTD-----------------LKV------------------------

-LLC--DTCS-TAE-FIEKFE----------I----------------------------

------------------------------------------------------------

--SHVLLATSKTCAQLLADKKILPHQINLVIVDECHK--S--I-NNSQLKFILQTIL---

----SCNN--VPRIIGLAVPLFNLT--QEPG--R-LGLEIEKVENTFQCEVETAS-DILS

I-----------------------------------------------------------

----LR---YSPKPKEYVVEY-AKS-EKGEL----HITI-ENHIL-----------HAIE

FLRD-----------H--RY-----------DPTEIY-N-EEFYEDIQKIPDPTEKPFEM

MQDLLHIL---------------------ETLGP----WCADRAAFAL------------

------------------------------------------------------------

--LILTEKLKIKTP-YERHYL------LLNM----------IASVFTKIRALCDNA----

----------------------------------------------------FEVL----

-SEKEKIYKYTTTKVHRLLQI----LK-TYTPYYT-------KHINNTDNKINNENISRK

FPANNR--DSIKDKFMQSKKLDCNWK-------------------------------SSE

ENCKKPP------VARHMRGITD-PDLLCGVIFVDKAFVAK-----VLFYLLNEISMHDE

E--------LHF-LSPLYTIEKNI-DDISYSKDLEIEHR---------------------

--------------------------------------------KQEEVLKRFR------

IHECNILISTS--------ILEEGIDIPKCNFVMRYDFPKTYQSYV-------QCKSRAR

APDALYVLLVS-------------------------------------------------

----------------------------QEM-----------------------------

---------------------------------------SKEYVWQLAQYHYIEKTLLVK

CSNNEPSEEEENE--ADM-YAAMIPHYKP----LDGGDAPKVTFNSAISLVNRY------

------CAKLPSD------T--FTRLTP----------------EWSIQEMNI-DNILM-

-----YICSLRLPINSPVKYIVS-S-----YPMPNKAMARR------------MAALQLC

IDL------HRKNEIDDNLLP-IGKENFKAKPEDAEVPAL--PD-----------ESKVD

FS-------EARPG---TTKRR--Q---YYYKKTA--EALTDCRPIIG------VPSYLY

H----INL------VLSCPLPEEQNT------------RGR-------------------

--RIY----------PPEESAIGFGI-ITLK------EIPK----LCPFPIY--------

TRSGEVHVKLK-LTKQT--------------IILNEMQV---------------------

EKIATFL-NYTFTNVLR--------------------------LQQYL-MLF--------

----------------------------------DPNA-SENSYMIVP-V-KLDEQSDIA

VDW--------NF------LECIYKN-RNIGPTKIPEEARKNF-----------------

-----------------------------------------------------K------

--------------------------------------FDASK-----YH---DAVIMP-

------------W---YRSQDQPQY----FYVAE--------------------------

ICTNLNPKSSFPGN-------------D-----------YG-------------------

TFEE----------YYLKKYDI---QIQNL------DQPLLDVDHTSA-RLN--------

----------------------FLTPRYV------------------N--------RKGV

A----------LPTSSEETKR---------------------------------------

------------AKRENL---EQKQ--------ILVAELC--AIH---------------

---------------------------PFP-------ASLWRQAVCL-PCILYRINALLL

ANQIRCQVAQMI-NLGQENL-NSDFEWPALDFGWSLAE---VLKKSKE--SEKTKLIRNE

-LNSTNAIDTKCQNISKD------------ITENLYKLDKEDLNESNYEFQLTNDKITEA

EQISNDENELE-IGTWSSDMAMNSLD--F-----G-ANNLKSFPLNVTVLPNEFNWND--

IRYGSPA------CESDFDG------------------------YESDDIYSDGF-----

------------ADTSDESEDENRGLRISYMGENVAEAVEDEKQISKQEVNKRILDLLET

E----KNSDEILWSYAKESEVLITKHKLAHYEFAKIRECEIKQNGSFIPYNNKIVIKR--

--------KTSFSLHSEENLPNYKYKTYVETVVDKFTQEIL--------------NNNET

IQPIKKNSNTPKFTYHFDSNL-------------------------------FSFDFQPK

LENHPGPSPSLILQALTMSN---ANDGINLERLETIGDSFLKYAITT-YLYCTYDN----

----------------------------------IHEGKLSHL-RSKQVSNLNLYR----

LGRQKMLGESMIATKFEPHDNWL-PPCYYV------------------------------

---------------PKE------------------LEQALIESGV--------------

PSTLWNQADIPALQAVN-PFEITQLV----------RETEQKLGVMK-------NELDRN

ETT------LPNN--L--DNMRCFIPYNLITQHSIPDKSIADCVEALIGAYLIA-CGPRG

-------------------------ALLFMA------------------WLGIHVLPTEE

INIIQET--------EPKDRIPGSTPFVKGKNEKGETIWTQIRYGKLEEPQNPLLRYIVD

PEEELKLMLD--------GYEELE--------------KNIGYKF--HDISYLL---QAF

THAS-YQPNRL-------------------------------------------------

--------TDC-------------------Y-----------------------------

-------------------------------------QR--LEFLGDAVLDYLITRHLY-

----ED--TRQHS-----------------------------------------------

------------------------------------------------------------

------------------------PGALTDLRSALVNNTIFASLAVRC-----------G

FHKYFRH------------------LSPGLSIVINRFVRIQEENGHSISEEYYL-IGEEE

CE----EAED--------------------------------------------------

-------------------VEVPKALGDVFESLAGAIYL-DSGMSLDAVWSVYYAIMKNE

IE---QFSTNVPK-SPIRELLELEPE--TAKFG--------KPE-KLADGRRVRVTVD--

--------------------VFGKGS---FKGI-----------GRNYRI--AKCTAAK-

------CALKKLKRM----------------QNYSREKL---------------------

-

>Dicerlike_3_Arabidopsis_thaliana

------MHSSLEPEKMEEGGGSNSLKRKFSEIDGDQNLDSVSSPMMTDSNGYELKVYEVA

KNRNIIAVLGTGIDKS----EITKRLIK----------AMGSSDTDKRLIIFLA----PT

VNLVKQQCCEIRALVN-----------------LKVEEYF--------------------

GAKG--VDKW-TSQRWDEEFS----------K----------------------------

------------------------------------------------------------

--HDVLVMTPQILLDVLRSAFLKLEMVCLLIIDECHH-----TTGNHPYAKLMKEFYH--

------ESTSKPKIFGLTASAVI------------RKAQVSELERLMDSKIFNPE-EREG

V-----------------------------------------------------------

----EK---FATTVKEGPILYNPSPSCSLEL----KEKL-ETSHL-----------KFDA

SLRR-----------L-------------------QELGKDSFLNMDNKFETYQKRLSID

YREILHCL---------------------DNLGL----ICAHLAAEVC------------

------------------------------------------------------------

-----LEKISDTKEESETYKE------CSMV----------CKEFLEDILSTIGVY----

-----------------------------------------------LPQDDKSLVDLQQ

NHLSAVISGHVSPKLKELFHL----LD-SFRG----------------------------

------------------------------------------------------------

------------------------DKQKQCLILVERIITAK-----VIERFVKKEAS---

---------LAY-LNVLYLTENNPSTNVSAQK----------------------------

--------------------------------------------MQIEIPDLFQ------

HGKVNLLFITD--------VVEEGFQVPDCSCMVCFDLPKTMCSYS-------QSQKHAK

QSNSKSIMFLE-------------------------------------------------

----------------------------RGN-----------------------------

----------------------------------------PKQRDHLHDLMRREVLIQD-

---------------PEAPNLKSCPPPVK-----NGH---------GVKEIG--------

------SMVIPDS------N--ITVSEE----------------AASTQTMSD-------

------------------------------PPSRNEQ-------------------LPPC

KKL------RLD---NNLLQS-NGKEKVASSKSKSSSSAAG-------------------

-----------------SKKRK--E---LHGTTCA--NALSGTWGEN-------IDGATF

Q----AYK------FDFCCNISGEVY------------SSF-------------------

--SLL----------LESTLAEDVG--------------------KVEMDLY--------

LVRKLVKASVS--PCGQ--------------IRLSQEEL---------------------

VKAKYFQ-QFFFNGMFG------------------KLFVGSKSQGTKREFLL--------

------------------------------QTDTSSLWHPAFMFLLLP-VETNDLASSAT

IDW--------SAINSCASIVEFLKKNSLLDLRDSDGNQCNTS-----------SG----

---------------------QE-----------------VLLDDKMEETNLIH------

--------------------------------------FANASSDKNSLE---ELVVIA-

------------I---HTGRI--------YSIVE--------------------------

AVSDSSAMSPFEVD-------------A--------SSGYA-------------------

TYAE----------YFNKKYGI---VLAHP------NQPLMKLKQSHH-AHN--------

----------------------LLVDFNE-------------------------------

------------EMVVKTEPK---------------------------------------

------------AGNVRKRKPNIHA--------HLPPELL--ARI---------------

---------------------------DVP-------RAVLKSIYLL-PSVMHRLESLML

ASQLREEIDCSI------------------------------------------------

------------------------------------------------------------

-----------------DNFSISSTS----------------------------------

------------------------------------------------------------

------------------------------------------------------------

------------------------------------------------------------

------------------------------------------------------------

------------------------------------------------------------

-----------ILEAVTTLT---CPESFSMERLELLGDSVLKYVASC-HLFLKYPD----

----------------------------------KDEGQLSRQ-RQSIISNSNLHR----

LTTSRKLQGYIRNGAFEPRR-WT-APGQF-------------------------------

------------------------------------------------------------

-----------SLFPVPCKCGIDTRE-----------VPLDPKFFTE-------NMTIKI

GKS------------------------CDMGHRWVVSKSVSDCAEALIGAYYVS-GGLSA

-------------------------SLHMMK------------------WLGIDV-----

------------------------------------------DFDPNLVVEAINRVSLRC

YIPKEDELIE------------LE--------------RKIQHEF--SAKFLLK---EAI

THSS-LRESYS-------------------------------------------------

------------------------------Y-----------------------------

-------------------------------------ER--LEFLGDSVLDFLITRHLF-

----NT--YEQTG-----------------------------------------------

------------------------------------------------------------

------------------------PGEMTDLRSACVNNENFAQVAVKN-----------N

LHTHLQR------------------CATVLETQINDYLMSFQKPDETGRSIPS-------

------------------------------------------------------------

-------------------IQGPKALGDVVESIAGALLI-DTRLDLDQVWRVFEPLLSPL

VT-----PDKLQL-PPYRELNELCDS--LGYFF--------RVK-CSNDGVKAQATIQ--

--------------------LQLDDVLLTGDGS-----------EQTNKL--ALGKAAS-

------HLLTQLEKR----------------NISRKTSLGDNQ-----------------

-

>Endoribonuclease_Dicer_homolog_3a_Oryza_sativa_Japonica

----------MNPLKRSLESSSQEHEAGKQKLQKRECQDF-------TPRRYQLDVYEVA

MRRNTIAMLDTGAGKT----MIAVMLIK---EFGKI--NRTK--NAGKVIIFLA----PT

VQLVTQQCEVIEIHTD-----------------FEVEQYY--------------------

GAKG--VDQW-TGPRWQEQIS----------K----------------------------

------------------------------------------------------------

--YQVMVMTPQVFLQALRNAFLILDMVSLMIFDECHH-----ATGNHPYTRIMKEFYH--

------KSEHKPSVFGMTASPVIRKGISSHL--D-CEGQFCELENLLDAKIYTVS-DREE

I-----------------------------------------------------------

----EF---CVPSAKEMCRYYDSKPVCFEDL----SEEL-GVLCS-----------KYDA

LITE-----------L-------------------QNKRSDMYKDADDITKESKRRLSKS

IAKICYCL---------------------DDVGL----ICASEATKIC------------

------------------------------------------------------------

--IERGQEKGWLKEVVDATDQQTDANGSRLFAENSALHMKFFEEALHLIDKRLQQG----

---------------------------------------------------IDMLLNSES

GCVEAAKTGYISPKLYELIQI----FH-SFSN----------------------------

------------------------------------------------------------

------------------------SRHARCLIFVDRKITAR-----VIDRMIKKIGH---

---------LAH-FTVSFLTGGRSSVDALTPK----------------------------

--------------------------------------------MQKDTLDSFR------

SGKVNLLFTTD--------VAEEGIHVPECSCVIRFDLPRTTRSYV-------QSRGRAR

QEDSQYILMIE-------------------------------------------------

----------------------------RGN-----------------------------

----------------------------------------VKQNDLISAIVRSETSMVKI

ASSRESGNLS-----PGFVPNEEINEYHVGT---TGA---KVTADSSISIVYRY------

------CEKLPQD------K--CYSPKP----------------TFEFTHHDD-G-----

-----YVCTLALPPSAVLQILVG-------PKARNMHKAKQ------------LVCLDAC

KKL------HELGALDDHLC-----------------LSVEDPV-----------PEIVS

KN------KGTGIG---TTKRK--E---LHGTTRI--HAWSGNWVSK-------KTALKL

Q----SYK------MNFVCDQAGQIY------------SEF-------------------

--VLL----------------------IDAT-------LPD-EVATLEIDLY--------

LHDKMVKTSVS--SCGL--------------LELDAQQM---------------------

EQAKLFQ-GLLFNGLFG------------------KLFTRSKVPNAPREFIL--------

------------------------------NKEDTFVWNTASVYLLLP--TNPSFDSNVC

INWS---------------VIDAAATAVKLMRRIYSENKRELL-----------------

----------------------------------------------------GI------

--------------------------------------FDSDQNVGDLIHLANKSCKAN-

------------S---LKDMVVLAVHTGKIYTAL--------------------------

DITELSGDSAFDGA-------------S-----DKKECKFR-------------------

TFAE----------YFKKKYGI---VLRHP------SQPLLVLKPSHN-PHN--------

----------------------LLSSKFR------------------D--------EGNV

VEN----------------MS---------------------------------------

------------NGTPVVNKTSNRV--------HMPPELL--IPL---------------

---------------------------DLP-------VEILRSFYLF-PALMYRIESLTL

ASQLRSEIGYS-------------------------------------------------

------------------------------------------------------------

------------------DSNISSFL----------------------------------

------------------------------------------------------------

------------------------------------------------------------

------------------------------------------------------------

------------------------------------------------------------

------------------------------------------------------------

-----------ILEAITTLR---CSEDFSMERLELLGDSVLKYAVSC-HLFLKFPN----

----------------------------------KDEGQLSSI-RCHMICNATLYK----

LGIERNVQGYVRDAAFDPRR-WL-APGQL-------------------------------

------------------------------------------------------------

-------------SIRPSPCECPVKS----------EVVTDDIHIID-------DKAIVL

GKA------------------------CDKGHRWMCSKTIADCVEAIIGAYYAG-GGLRA

-------------------------AMAVLK------------------WLGIGA-----

------------------------------------------EIEEDLIVQAILSASVQT

YLPKDNVFEM------------LE--------------AKLGYSF--SVKGLLV---EAL

THPS-QQELGA-------------------------------------------------

--------KYC-------------------Y-----------------------------

-------------------------------------ER--LEFLGDAVLDILLTRYLF-

----NS--HKDTN-----------------------------------------------

------------------------------------------------------------

------------------------EGELTDLRSASVNNENFAQVAVKH-----------N

FHHFLQH------------------SSGLLLDQITEYVNRLEGSSMDKVELLSDGL----

------------------------------------------------------------

-------------------PKGPKVLGDIVESIAGAILL-DTKLDLDVVWGIFEPLLSPI

VT-----PENLEL-PPYRELIEWCGK--HGYFV--------GIN-CRDQGDTVVATLD--

--------------------VQLKEVLLVRQGF-----------SKKRKD--AKAHASS-

------LLLKDLEEK----------------GLIIPKNASKTEQFEKHCGSTN-------

-

>Dicerlike_4_Arabidopsis_thaliana

DVSSDLHLTSSSSVSSFSSSSSSLFSAAGTDDPSPKMEKDPRK----IARRYQVELCKKA

TEENVIVYLGTGCGKT----HIAVMLIY---ELGHL--VLSP---KKSVCIFLA----PT

VALVEQQAKVIADSVN-----------------FKVAIHC--------------------

GGKR----IVKSHSEWEREIA----------A----------------------------

------------------------------------------------------------

--NEVLVMTPQILLHNLQHCFIKMECISLLIFDECHH---AQQQSNHPYAEIMKVFYKS-

------ESLQRPRIFGMTASPVVGKGSFQSE--N-LSKSINSLENLLNAKVYSVE-SNVQ

L-----------------------------------------------------------

----DG---FVSSPLVKVYYYRSALSDASQSTIRYENML-EDIKQ-----------RCLA

SLKL-----------L-----------------IDTHQT--------QTLLSMKRLLKRS

HDNLIYTL---------------------LNLGL----WGAIQAAK--------------

------------------------------------------------------------

--IQLNSDHNVQDEPVGKNPK-------SKI----------CDTYLSMAAEALSSG----

--------------------------------------------------VAKDENASDL

LSLAALKEPLFSRKLVQLIKI----LS-VFRL----------------------------

------------------------------------------------------------

------------------------EPHMKCIIFVNRIVTAR-----TLSCILNNLEL---

---------LRS-WKSDFLVGLSSGLKSMSRR----------------------------

--------------------------------------------SMETILKRFQ------

SKELNLLVATK--------VGEEGLDIQTCCLVIRYDLPETVTSFI-------QSRGRAR

MPQSEYAFLVD-------------------------------------------------

----------------------------SGN-----------------------------

----------------------------------------EKEMDLIENFKVNEDRMNLE

ITYRSSEET------CPRLDEELYKVHETGACISGGS---------SISLLYKY------

------CSRLPHD------E--FFQPKP----------------EFQFKPVDEFG-----

----GTICRITLPANAPISEIES-------SLLPSTEAAKK------------DACLKAV

HEL------HNLGVLNDFLLP-DSKDEIEDELSDDE-FDFDNIK-----------GE---

-----------------GCSRG--D---LYEMRVP--VLFKQKWDPS-------TSCVNL

H----SYY------IMFVPHPADRIY------------KKF-------------------

--GFF----------------------MKS-------PLPV-EAETMDIDLH--------

-LAHQRSVSVKIFPSGV--------------TEFDNDEI---------------------

RLAELFQ-EIALKVLFE----------------------RGELIPDFVPLEL--------

--------------------------------QDSSRTSKSTFYLLLP-LCLHDGESVIS

VDWVT--------------IRNCLSSPIFKTPSVLVEDIFPPS-----------------

--------------------------------------------GSHLKLANGC------

--------------------------------------WNIDD-----VK---NSLVFT-

------------T---YSKQF--------YFVAD--------------------------

ICHGRNGFSPVKES-------------S-----------TK-------------------

SHVE----------SIYKLYGV---ELKHP------AQPLLRVKPLCH-VRN--------

----------------------LLHNRMQ-------------------------------

------------------------------------------------------------

------------TNLEPQELDEYFI--------EIPPELSHLKIK---------------

---------------------------GLS-------KDIGSSLSLL-PSIMHRMENLLV

AIELKHVLSASI------------------------------------------------

------------------------------------------------------------

----------------PEIAEVSGHR----------------------------------

------------------------------------------------------------

------------------------------------------------------------

------------------------------------------------------------

------------------------------------------------------------

------------------------------------------------------------

-----------VLEALTTEK---CHERLSLERLEVLGDAFLKFAVSR-HLFLHHDS----

----------------------------------LDEGELTRR-RSNVVNNSNLCR----

LAIKKNLQVYIRDQALDPTQFFA-------------------------------------

------------------------------------------------------------

-------------FGHPCRVTCDEVA----------SKEVHSLNRDL-------GILESN

TGE----------------------IRCSKGHHWLYKKTIADVVEALVGAFLVD-SGFKG

-------------------------AVKFLK------------------WIGVNV-----

------------------------------------------DFESLQVQDACIASRRYL

PLTTRNNLET------------LE--------------NQLDYKF--LHKGLLV---QAF

IHPS-YNRHGG-------------------------------------------------

---------GC-------------------Y-----------------------------

-------------------------------------QR--LEFLGDAVLDYLMTSYFF-

----TV--FPKLK-----------------------------------------------

------------------------------------------------------------

------------------------PGQLTDLRSLSVNNEALANVAVSF-----------S

LKRFLFC------------------ESIYLHEVIEDYTNFLASSPLASGQSEG-------

------------------------------------------------------------

-------------------PRCPKVLGDLVESCLGALFL-DCGFNLNHVWTMMLSFLDPV

KN-----LSNLQI-SPIKELIELCQS--YKWDR--------EIS-ATKKDGAFTVELK--

--------------------VTKNGCCLTVSAT-----------GRNKRE--GTKKAAQ-

------LMITNLKAH----------------ENITTSHPLEDVLKNGI------------

-

>Endoribonuclease_Dicer_homolog4_isoform_X1_Oryza_sativa_Japonica

--------------MGDAAAAAPAAAAAGPSSTRGEPKDPRT-----IARKYQLDLCKRA

VEENIIVYLGTGCGKT----HIAVLLIY---ELGHL--IRKP---SREVCIFLA----PT

IPLVRQQAVVIASSTD-----------------FKVQCYY--------------------

GNGK----NSRDHQEWENDMR----------E----------------------------

------------------------------------------------------------

--FEVLVMTPQILLQSLRHCFIKMNSIALLILDECHH--AQPQ-KRHPYAQIMKEFYN--

--SNSVEK--FPRVFGMTASPIIGKGGSNKL--N-YTKCINSLEELLHAKVCSV--DNEE

L-----------------------------------------------------------

----ES---VVASPDMEVYFY-GPVN---------HSNL-TTICI-----------KELD

SLK------------------------------LQSERMLRASLCDFKDSQKKLKSLWRL

HENIIFCL---------------------QELGS----FGALQAARTF------------

------------------------------------------------------------

--LSFDGDKLDRRE-VDLNGS------TSSF----------AHHYLNGATSILSRNK---

----------------------------------------------------TDGSHAGS

FDLEKLEEPFFSNKFSVLINV----LS-RYGL----------------------------

------------------------------------------------------------

------------------------QENMKCIVFVKRITVAR-----AISNILQNLKC---

---------LEF-WKCEFLVGCH-------SGSKNMSRN---------------------

--------------------------------------------KMDAIVQRFS------

SGEVNLLVATS--------VGEEGLDIQTCCLVVRFDLPETVASFI-------QSRGRAR

MTKSKYVVLLE-------------------------------------------------

----------------------------REN-----------------------------

----------------------------------------QSHEKLLNGYIAGESIMNEE

IDSRTSNDMFD----CLEEN-----IYQVDN---TGA---SISTACSVSLLHCY------

------CDNLPRD------M--FFTPSP----------------VFFYIDGIEG------

-----IICRLILPPNAAFRQAD--G-----QPCLSKDEAKR------------DACLKAC

VKL------HKLGALTDFLLP--GPGSRKNKVSVTNNSSNNKVE-----------DDSL-

--------------------RE--E---LHEMLIP--AVLKPSGLKL-------DSLSNL

H----FYY------VKFIPIPEDRRY------------QM--------------------

-----------------------FGL-FVIN------PLPVE-AETLQVDLH--------

LARGRIVKAGI-KHLGK--------------IAFEKEKM---------------------

MLAHKFQ-EMCLKILLDRS----------------------EFTSPHVKLGN--------

---------------------------------DVTLEINSTFYLLLP-IKQKCYGDRFM

IDW--------PA------VERCLSSPIFKDPIDVSVHASYSS-----------------

--------------------------------------------NESLRLLDGI------

--------------------------------------FSKTD-----VV---GSVVFS-

------------P---HNN----IF----FFVDG--------------------------

ILDEINAWSEHSG---------------------------A-------------------

TYAE----------HFKERFRI---ELSHP------EQPLLKAKQIFN-LRN--------

----------------------LLHNRLP-------------------------------

-------------------ET---------------------------------------

------------TESEGRELLEHFV--------ELPPELCSLKVI---------------

---------------------------GFS-------KDMGSSLSLL-PSLMYRLENLLV

AIELKDVMLSSF------------------------------------------------

------------------------------------------------------------

----------------PEASQISASG----------------------------------

------------------------------------------------------------

------------------------------------------------------------

------------------------------------------------------------

------------------------------------------------------------

------------------------------------------------------------

-----------ILEALTTEK---CLERISLERFEVLGDAFLKYVVGR-HKFITYEG----

----------------------------------LDEGQLTRR-RSDVVNNSHLYE----

LSIRKKLQVYIRDQQFEPTQ-FF-APGRP-------------------------------

------------------------------------------------------------

------------------CKVVCNTD--------VEVRLHQMDIHPD-------NRENCN

LRC-------------------------TRSHHWLHRKVIADVVESLIGAFLVE-GGFKA

-------------------------AFAFLH------------------WIGIDV-----

------------------------------------------DFNNPALYRVLDSSSINL

SLMDYTDIAG------------LE--------------ELIGYKF--KHKGLLL---QAF

VHPS-FSQHSG-------------------------------------------------

---------GC-------------------Y-----------------------------

-------------------------------------QR--LEFLGDAVLEYVITSYLY-

----ST--YPDIK-----------------------------------------------

------------------------------------------------------------

------------------------PGQITDLRSLAVGNDSLAYAAVEK-----------S

IHKHLIK------------------DSNHLTSAISKFEMYVKLSNSEKDLLEE-------

------------------------------------------------------------

-------------------PACPKALGDIVESCIGAVLL-DSGFNLNYVWKVMLMLLKPV

LT-----FANMHT-NPMRELRELCQC--HGFEL--------GLPKPMKADGEYHVKVE--

--------------------VNIKSKIIICTAA-----------NRNSKA--ARKFAAQ-

------ETLSKLKNY----------------GYKHRNKSLEEILIVAR------------

-

>Dicerlike_1_Arabidopsis_thaliana

-SGTWEADHERDVKKVSGGNRECDVKAEENKSKPEERKEKVVEE---QARRYQLDVLEQA

KAKNTIAFLETGAGKT----LIAILLIK---SVHKD--LMSQ--NRKMLSVFLV----PK

VPLVYQQAEVIRNQTC-----------------FQVGHYC--------------------

GEMG--QDFW-DSRRWQREFE----------S----------------------------

------------------------------------------------------------

--KQVLVMTAQILLNILRHSIIRMETIDLLILDECHH-----AVKKHPYSLVMSEFYH--

----TTPKDKRPAIFGMTASPVNLKGVSSQV--D-CAIKIRNLETKLDSTVCTIK-DRKE

L-----------------------------------------------------------

----EK---HVPMPSEIVVEY-----DKAATMWSLHETI-KQMIA-----------AVEE

AAQA-----------S--SRKSKWQFMGARDAGAKDELR-QVYGVSERTESDGAANLIHK

LRAINYTL---------------------AELGQ----WCAYKVGQSF------------

------------------------------------------------------------

--LSALQSDERVNFQVDVKFQ------ESYL----------SEVVSLLQCELLEGA----

------------------A--AEKVAAEVGKPENGNAHDEMEEGELPDDPVVSGGEHVDE

VIGAAVADGKVTPKVQSLIKL----LL-KYQH----------------------------

------------------------------------------------------------

------------------------TADFRAIVFVERVVAAL-----VLPKVFAELPS---

---------LSF-IRCASMIGHNNSQEMKSS-----------------------------

--------------------------------------------QMQDTISKFR------

DGHVTLLVATS--------VAEEGLDIRQCNVVMRFDLAKTVLAYI-------QSRGRAR

KPGSDYILMVE-------------------------------------------------

----------------------------RGN-----------------------------

----------------------------------------VSHAAFLRNARNSEETLRKE

AIERTDLSHLKDTSRLISIDAVPGTVYKVEA---TGA---MVSLNSAVGLVHFY------

------CSQLPGD------R--YAILRP----------------EFSMEKHEKPG-----

-GHTEYSCRLQLPCNAPFEILEG-------PVCSSMRLAQQ------------AVCLAAC

KKL------HEMGAFTDMLLP--------------DKGSGQDAE-----------KADQD

DE------GEPVPG---TARHR--E---FYPEGVA--DVLKGEWVSSGKEVC--ESSKLF

H----LYM------YNVRCVDFGSSK------------DPF-------------------

--LSE----------VSE-----FAI-LFGN------ELDA-EVLSMSMDLY--------

VARAMITKASL-AFKGS--------------LDITENQL---------------------

SSLKKFH-VRLMSIVLDVDVE---------------------------------------

--------------------------------PSTTPWDPAKAYLFVPVTDNTSMEPIKG

INW--------ELVEKITKTTAWDNPLQRARPDVYLGTNERTLGGDRREYGFGKLR----

---------------------HN-----------------IVFGQKSHPTYGIR------

--------------------------------------GAVAS-----FDVVRASGLLP-

------------VRDAFEKEVEEDLSKGKLMMADGCMVAEDLIGKIVTAAHSGKRFYVDS

ICYDMSAETSFPRK-------------E----GYLGPLEYN-------------------

TYAD----------YYKQKYGV---DLNCK------QQPLIKGRGVSY-CKN--------

----------------------LLSPRFE------------------Q--------S---

------------------------------------------------------------

------------GESETVLDKTYYV--------FLPPELC--VVH---------------

---------------------------PLS-------GSLIRGAQRL-PSIMRRVESMLL

AVQLKNLI----------------------------------------------------

------------------------------------------------------------

------------------SYPIPTSK----------------------------------

------------------------------------------------------------

------------------------------------------------------------

------------------------------------------------------------

------------------------------------------------------------

------------------------------------------------------------

-----------ILEALTAAS---CQETFCYERAELLGDAYLKWVVSR-FLFLKYPQ----

----------------------------------KHEGQLTRM-RQQMVSNMVLYQ----

FALVKGLQSYIQADRFAPSR-WS-APGVP-------------------------------

----------------------------------------------------------PV

FDEDTKDGGSSFFDEEQK--PVSEEN----------SDVFEDGEMED-------GELEGD

LSS----------------------------YRVLSSKTLADVVEALIGVYYVE-GGKIA

-------------------------ANHLMK------------------WIGIHV-----

-----------------------------------------------EDDPDEVDGTLKN

VNVPESVLKS-------IDFVGLE--------------RALKYEF--KEKGLLV---EAI

THAS-RPSSGV-------------------------------------------------

---------SC-------------------Y-----------------------------

-------------------------------------QR--LEFVGDAVLDHLITRHLF-

----FT--YTSLP-----------------------------------------------

------------------------------------------------------------

------------------------PGRLTDLRAAAVNNENFARVAVKH-----------K

LHLYLRH------------------GSSALEKQIREFVKEVQTESSKPGFNSF-------

------GLGD--------------------------------------------------

-------------------CKAPKVLGDIVESIAGAIFL-DSGKDTTAAWKVFQPLLQPM

VT-----PETLPM-HPVRELQERCQQ--QAEGL--------EYK-ASRSGNTATVEVF--

--------------------IDGVQV---GVAQ-----------NPQKKM--AQKLAAR-

------NALAALKEK-----------EIAESKEKHINNGNAGE-----------------

-

>Endoribonuclease_Dicer_homolog_1_Oryza_sativa_Japonica

-SGTWEQESDREAKRARTQDGGSMEKKAEADRMGAAQREKPVAEE--RARQYQLEVLEQA

KSRNTIAFLETGAGKT----LIAVLLIK---SVCDK--MLKE--NKKMLAVFLV----PK

VPLVYQQAEVIRDRTG-----------------YRVGHYC--------------------

GEMG--QDFW-DARKWQREFE----------S----------------------------

------------------------------------------------------------

--KQVLVMTAQILLNILRHSIIKMDAIHLLILDECHH-----AVKKHPYSLVMSEFYH--

----TTPKEKRPAVFGMTASPVNLKGVTSQE--D-CAIKIRNLESKLDSVVCTIK-DRKE

L-----------------------------------------------------------

----EK---HVPMPLEVVVQY-----DKAATLWSLHEQI-KQMES-----------TVEE

AALS-----------S--SKRTKWQFMGARDAGSRDELR-LVYGVSERTESDGAANLIQK

LRAINYAL---------------------GELGQ----WCAYKVAQSF------------

------------------------------------------------------------

--LTALQNDERANYQVDVKFQ------ESYL----------KKVVDLLHCQLTEGA----

------------------AMKSETSDVEMQNTEKHNTND-LEEGELPDSHAVSVGEHVDE

VIGAAVADGKVTPRVQALIKI----LL-KYQH----------------------------

------------------------------------------------------------

------------------------TEDFRAIIFVERVVTAL-----VLPKVLAELPS---

---------LSF-IRCASLIGHNNNQEMRAC-----------------------------

--------------------------------------------QMQDTISKFR------

DGRVTLLVATS--------VAEEGLDIRQCNVVIRFDLAKTVLAYI-------QSRGRAR

KPGSDYILMLE-------------------------------------------------

----------------------------RGN-----------------------------

----------------------------------------ISHETFLRNARNSEETLRKE

AMERTDLSHLDGTSVLSPVDTSPGSMYQVES---TGA---VVSLNSAVGLIHFY------

------CSQLPSD------R--YSILHP----------------EFIMQKYEKPG-----

-GSVEYSCKLQLPCNAPFEKLEG-------PICSSIRLAQQ------------AVCLAAC

KKL------HEMGAFTDTLLP--------------DRGSG-EGE-----------KTEQN

DE------GEPLPG---TARHR--E---FYPEGVA--DILRGEWILSGRDGY--QNSQFI

K----LYM------YSVNCVNVGTSK------------DPF-------------------

--VTQ----------LSN-----FAI-IFGN------ELDA-EVLSTTMDLF--------

VARTMITKASL-VFRGR--------------IEITESQL---------------------

VLLKSFH-VRLMSIVLDVDVD---------------------------------------

--------------------------------PSTTPWDPAKAYLFVPVGAEKCTDPLRE

IDW--------TLVNNIVNTDAWNNPLQRARPDVYLGTNERTLGGDRREYGFGKLR----

---------------------HG-----------------TAFGQKAHPTYGIR------

--------------------------------------GAIAE-----FDIVKASGLVP-

------------ARD--RGHFSDYQNQGKLFMADSCWNAKDLAGMVVTAAHSGKRFYVDC

ICYNMNAENSFPRK-------------E----GYLGPLEYS-------------------

SYAD----------YYKQKYGV---ELIYR------KQPLIRARGVSY-CKN--------

----------------------LLSPRFE------------------H--------SDA-

-------------------RE---------------------------------------

------------GDFSENLDKTYYV--------YLPPELC--LVH---------------

---------------------------PLP-------GSLVRGAQRL-PSIMRRVESMLL

AVQLKDII----------------------------------------------------

------------------------------------------------------------

------------------DYPVPATK----------------------------------

------------------------------------------------------------

------------------------------------------------------------

------------------------------------------------------------

------------------------------------------------------------

------------------------------------------------------------

-----------ILEALTAAS---CQETLCYERAELLGDAYLKWVVSR-FLFLKYPQ----

----------------------------------KHEGQLTRM-RQQMVSNMVLYQ----

YALNKTLQSYIQADRFAPSR-WA-APGVL-------------------------------

----------------------------------------------------------PV

FDEESREYEPSIFDEESTGCELQKES----------YDDYADNMQED-------GEIEGD

SSC----------------------------YRVLSSKTLADVVEALIGVYYVA-GGKIA

-------------------------ANHLMK------------------WIGIHA-----

------------------------------------------ELDPEEIPPPKPYDI-PE

SIMRSINFDT------------LK--------------GVLGIEF--QNKGLLV---EAI

THAS-RPSSGV-------------------------------------------------

---------SC-------------------Y-----------------------------

-------------------------------------QR--LEFVGDAVLDHLITRHLF-

----FT--YTDLP-----------------------------------------------

------------------------------------------------------------

------------------------PGRLTDLRAAAVNNENFARVAVKH-----------K

LHVHLRH------------------GSSALETQIREFVKDVQEELLKPGFNSF-------

------GLGD--------------------------------------------------

-------------------CKAPKVLGDIVESIAGAIFL-DSGYDTSVVWKVFQPLLHPM

VT-----PETLPM-HPVRELQERCQQ--QAEGL--------EYK-ASRAGNIATVEVF--

--------------------VDGVQI---GVAQ-----------NPQKKM--AQKLAAR-

------NALVVLKEK----------------ETATKKE----------------------

-

>Dicerlike_2_Arabidopsis_thaliana

--------------------MTMDADAMETETTDQVSASPLH-----FARSYQVEALEKA

IKQNTIVFLETGSGKT----LIAIMLLR---SYAYL--FRKP---SPCFCVFLV----PQ

VVLVTQQAEALKMHTD-----------------LKVGMYW--------------------

GDMG--VDFW-DSSTWKQEVD----------K----------------------------

------------------------------------------------------------

--YEVLVMTPAILLDALRHSFLSLSMIKVLIVDECHH-----AGGKHPYACIMREFYHKE

LNSGTSN---VPRIFGMTASLVKTKGENLDS----YWKKIHELETLMNSKVYTCE-NESV

L-----------------------------------------------------------

----AG---FVPFSTPSFKYY-QHIKIPSPK----RASL-VEKLE-----------RLT-

---------------------------------IKHRLSLGTLDLNSSTVDSVEKRLLRI

SSTLTYCL---------------------DDLGI----LLAQKAAQSL------------

------------------------------------------------------------

-SASQNDSFLWGELNMFSVAL------VKKF----------CSDASQEFLAEIPQG----

------------------------------------------------------LNWSVA

NINGNAEAGLLTLKTVCLIET----LL-GYSS----------------------------

------------------------------------------------------------

------------------------LENIRCIIFVDRVITAI-----VLESLLAEILPNCN

N------------WKTKYVAGNN-------SGLQNQTRK---------------------

--------------------------------------------KQNEIVEDFR------

RGLVNIIVATS--------ILEEGLDVQSCNLVIRFDPASNICSFI-------QSRGRAR

MQNSDYLMMVE-------------------------------------------------

----------------------------SGD-----------------------------

----------------------------------------LLTQSRLMKYLSGGKRMREE

SLDHSLVPC------PPLPDDSDEPLFRVES---TGA---TVTLSSSVSLIYHY------

------CSRLPSD------E--YFKPAP----------------RFDVNKDQG-------

------SCTLYLPKSCPVKEV---------KAEANNKVLKQ------------AVCLKAC

IQL------HKVGALSDHLVP------------DMVVAETVSQK-----------LEKIQ

Y----------------NTEQP--C---YFPPELV--SQFSA------------QPETTY

H----FYL------IRMKPNSP--------------------------------------

--RNF----------HLNDVLLGTRV-VLED------DIGN-----TSFRLE--------

DHRGTIAVTLS--YVGA--------------FHLTQEEV---------------------

LFCRRFQ-ITLFRVLLDHSVE--------------------NLMEALNGLHL--------

------------------------------------RDGVALDYLLVP---STHSHETSL

IDW--------EV------IRSVNLTSHEVLEKHENCSTNGAS-----------------

---------------------------------------------RILHTKDGL------

--------------------------------------FCTCV-----VQ---NALVYT-

------------P---HNGYV--------YCTKG--------------------------

VLNNLNGNSLLTKR-------------NSGDQ----------------------------

TYIE----------YYEERHGI---QLNFV------DEPLLNGRHIFT-LHS--------

----------------------YLH-----------------------------------

-------------------MA---------------------------------------

------------KKKKEKEHDREFV--------ELPPELCHVILS---------------

---------------------------PIS-------VDMIYSYTFI-PSVMQRIESLLI

AYNLKKSI----------------------------------------------------

------------------------------------------------------------

-----------------PKVNIPTIK----------------------------------

------------------------------------------------------------

------------------------------------------------------------

------------------------------------------------------------

------------------------------------------------------------

------------------------------------------------------------

-----------VLEAITTKK---CEDQFHLESLETLGDSFLKYAVCQ-QLFQHCHT----

----------------------------------HHEGLLSTK-KDGMISNVMLCQ----

FGCQQKLQGFIRDECFEPKG-WM-VPGQS-------------------------------

------------------------------------------------------------

---------------------------------------SAAYSLVN-------DTLPES

RNI------------------------YVASRRNLKRKSVADVVESLIGAYLSE-GGELA

-------------------------ALMFMN------------------WVGIKV-----

-----------------------------------------------DFTTTKIQRDSPI

QAEKLVNVGY------------ME--------------SLLNYSF--EDKSLLV---EAL

THGS-YMMPEI-------------------------------------------------

--------PRC-------------------Y-----------------------------

-------------------------------------QR--LEFLGDSVLDYLITKHLY-

----DK--YPCLS-----------------------------------------------

------------------------------------------------------------

------------------------PGLLTDMRSASVNNECYALVAVKA-----------N

LHKHILY------------------ASHHLHKHISRTVSEFEQSSLQSTFGWESD-----

------------------------------------------------------------

-------------------ISFPKVLGDVIESLAGAIFV-DSGYNKEVVFASIKPLLGCM

IT-----PETVKL-HPVRELTELCQ---KWQFE------------LSKAKDFDSFTVE--

--------------------VKAKEMSFAHTAK-----------ASDKKM--AKKLAYK-

------EVLNLLKNS--------------------LDY----------------------

-

>Endoribonuclease_Dicer_homolog_2a_isoform_X2_Oryza_sativa_Japonica

----------MGGPLTAAGGRGDGGAKAVEPLRPPPPPDPKT-----MARWYQLEALERA

VRGNTLAFLETGSGKT----LIAVMLLR---AYAHR--VRRP--DSRRFAVFLV----PT

VVLVGQQARVVEQHTD-----------------LVVKQFC--------------------

GEMG--VDFW-DAATWRSQLE----------D----------------------------

------------------------------------------------------------

--GEVLVMTPQILLDNLRHSFFRLQDIALLIFDECHH-----ARGNTPYACIFKEFYHPQ

LNSSASDP--LPRIFGMSASLIYSKDLNPHN----YSKQISEIENLMNSKVYTVD-SESA

L-----------------------------------------------------------

----SE---YIPFASTKIVDF-DDSNISSEL----HANI-LSCLN-----------RLNK

KAAE-----------V------------------------------------------QS

YEENSLSF------------------------------WGET------------------

------------------------------------------------------------

--LDKNVEGFIRNYSEEVHRE------LSCF-----------------------------

----------------------------------------------------LKNGHIGE

KFPADSQDGILTPKVHCLIRT----LL-QYRH----------------------------

------------------------------------------------------------

------------------------MQDLRCIVFVERVITSI-----VLEHLLSSIHQMSG

-------------WNVKHMAGSR-------PGLLSQSRK---------------------

--------------------------------------------NHTEIVESFR------

KGKVHIIIATQ--------ILEEGLDVPSCNLVIRFDPSATVCSFI-------QSRGRAR

MENSDYLLLVG-------------------------------------------------

----------------------------RGD-----------------------------

----------------------------------------VEAQTNAEKFLASGQIMREE

SLRLGSISCQPLENTLCEDT-----YYRVES---TRA---IVTLNSSVPLIHFF------

------CSKLPSD------E--YFNPLP----------------RFDIDKASG-------

------TCTLHLPKSSPVQTVNVEG---------EGSILKE------------TVCLKAC

QEL------HAIGALTDSLLP--------------ELDVPCDEE-----------PDIVV

EN------KIEQPS--------------YFPEEFV--DNWRSF-----------SRLGIY

Y----CYK------ISLEGCPKTASP------------TDI-------------------

--LLA----------LKCDLGSDFTS-SSF-------KLPG-------------------

---GQDNASVTMKYVGI--------------IHLNQEQV---------------------

IIARRFQ-TTILSFLIGDDH-----------------------LEVSNGIKY--------

--------------------------------FHEMQVPIGVVYLLLP-------LVSGR

IDWC---------------SMKFSSSPIYEANNKHMTHCHSCK-----------------

-------------------------------------------DIDLLQTKDGP------

--------------------------------------FCRCI-----LK---NSIVCT-

------------P---HNNI---------FYVIS--------------------------

GFLDLDANSCLPQH-------------D---------GTVV-------------------

TYKD----------YFKTRHGL---TLTFE------NQPLLAGSKHVK-VRN--------

----------------------FLHNCY--------------------------------

------------------------------------------------------------

------------SKKEKEPGDRYSV--------ELPPELCRIIMS---------------

---------------------------PVS-------ANNLHIFSYV-PSIMFRIQCMLL

SVKLKVQLGPTV------------------------------------------------

------------------------------------------------------------

-----------------QQFDVPVLK----------------------------------

------------------------------------------------------------

------------------------------------------------------------

------------------------------------------------------------

------------------------------------------------------------

------------------------------------------------------------

-----------ILEALTTKK---CQEEFSQESLETLGDSFLKYVTTR-HLFSEYRL----

----------------------------------QHEGILTKM-KKNLISNAALCQ----

LACSSNLVGYIHAEEFNPRD-WI-IPCLD-------------------------------

------------------------------------------------------------

---------------------YDERD-------------NKKISFLA-------PNGMYS

QRK-----------------------------MSIKSKRIADSVEALIGAYLST-AGEKA

-------------------------AFLLMK------------------SLGMNI-----

----------------------------------------------EFHTEIPVERKISM

KAEEFINVRS------------LE--------------GMLGYKF--NDSLLLL---EAL

THGS-YQTSGP-------------------------------------------------

--------TSC-------------------Y-----------------------------

-------------------------------------QR--LEFLGDAILDHLFTEYYY-

----SK--YPDCT-----------------------------------------------

------------------------------------------------------------

------------------------PELLTDLRSASVNNNCYAHAAVKS-----------G

LNKHILH------------------SSSELHRKMSYYLEEFGQSFTGPSYGWEAG-----

------------------------------------------------------------

-------------------IGLPKVLGDVIESIAGAIYL-DSKCDKEVVWRSMKRLLEPL

AT-----PETIEP-DPVKGLQEFCDR--RSFKI--------TYE-KNHVDGVSSVIAR--

--------------------VKAGETTYSATKS-----------GPCKLV--AKKLASK-

------AVLKDLIAG----------------HKDTEAAAV--------------------

-
